# Proceedings of the Nineteenth International Society of Sports Nutrition (ISSN) Conference and Expo

**DOI:** 10.1080/15502783.2023.2187955

**Published:** 2023-04-05

**Authors:** Trisha VanDusseldorp, Chad M. Kerksick, Erik Bustillo, Douglas Kalman, Jose Antonio

**Affiliations:** a Bonafide Health, LLC p/b JDS Therapeutics, Harrison, New York, USA; b Department of Health and Exercise Sciences, Jacksonville University, Jacksonville, Florida, USA; c Exercise and Performance Nutrition Laboratory, School of Health Sciences, Lindenwood University, St. Charles, Missouri, USA; d Train 8Nine, Miami, Florida, USA; e Dr. Kiran C. Patel College of Osteopathic Medicine, Nova Southeastern University, Davie, Florida, USA; f Department of Health and Human Performance, Nova Southeastern University, Davie, Florida, USA

**Diets of Female DII Collegiate Volleyball Players as Viewed Through the Lens of ISSN Position stands**

Holli Bragg^a^, Greg Popovich^a^, Kristy Henson^b^

^a^School of Exercise Science & Athletic Training, West Virginia Wesleyan College, Buckhannon, WV, USA; ^b^School of Science & Technology, Fairmont State University, Fairmont, WV, USA

Corresponding author: bragg.hr.2019@wvwc.edu

**Background:** This study provides insight into the dietary habits of in-season DII female collegiate volleyball players through comparison to ISSN position stands. Tracking dietary intake of the macronutrients is important for athletes to perform and recover optimally.

**Methods:** Participants (n = 10) were current DII female volleyball players over the age of 18. Participants tracked their normal daily food intake for three days using the USDA’s MyPlate meal tracking application. Participants then downloaded and submitted their data for anonymization and analysis. Findings were compared to the ISSN’s relevant position stands on protein, creatine, caffeine, meal frequency, nutrient timing, and energy drinks.

**Results:** The average caloric intake was 1,792.5 kilocalories per day (SD 496 kcal). The daily macronutrient intake averages were as follows: fat 71.4 g (SD 18.2 g), carbohydrate 181.5 g (2.62 g/kg body weight/day) (SD 65.9 g), and protein 70.8 g (1.0 g/kg body weight/day) (SD 25.1 g). When comparing the average intake of protein and carbohydrates to the ISSN position stands, the athletes were significantly below the recommended g/kg/day (p = 1.59E-05). When using the mean of the carbohydrate and protein range recommendations, average carbohydrate intake differed by −64.24% (SD 10.1) and protein consumption differed by −39.71% (SD 18.8). All athletes consumed less protein and carbohydrate than that recommended for highly-trained individuals. No athletes reported using creatine during the study. Fifty percent of participants consumed caffeinated beverages but none consumed energy drinks during the observation period.

**Conclusions:** The average daily caloric intake for these athletes was found to be markedly lower than the recommended ranges. This was attributed to significantly lower than recommended protein and carbohydrate consumption. Per the ISSN, the minimum recommended intake of calories for athletes is 2,500 kcal/day. The recommended intake ranges are 5-12 g/kg/per day and 1.4-2.0 g/kg/day for carbohydrates and protein, respectively; the players did not routinely reach even the lower end of these ranges. The recommended fat intake is 30% of total calories per day and the players’ fat intake was appropriate. No athlete reported using supplemental creatine during this observation; in contrast, the ISSN identifies volleyball as a sport that ‘may be enhanced’ by creatine supplementation. The results of this study suggest that this sample of DII volleyball players were substantially undernourished.

**Nutrition knowledge in New Zealand rugby union athletes**

Charlie Roberts^a^, Martyn Beaven^a^, Logan Posthumus^a,b,d^, Nicholas Gill^a,b,c^, Stacy Sims^a,c^

^a^Te Huataki Waiora School of Health, University of Waikato, Hamilton, New Zealand; ^b^New Zealand Rugby Union, Wellington, New Zealand; ^c^Sports Performance Research Institute New Zealand (SPRINZ), Auckland University of Technology, Auckland, New Zealand; ^d^Faculty of Health, Education and Environment, Toi Ohomai Institute of Technology, Tauranga, New Zealand

Corresponding author: charlie.jon.roberts@gmail.com

**Background:** Nutrition education and knowledge are known factors that can influence dietary intake in athletes. Sport nutrition practitioners can emphasize the importance of certain nutrition practices but the uptake by athletes is variable, depending on the environment in which the information is given. In New Zealand, there are unique developmental rugby academies, the goal of which is to provide young players with coaching support, professional level programming and access to facilities to prepare individuals for professional level play. Despite the inclusivity of the programmes, nutrition support is minimal. Due to the unusual demands of the developmental athlete’s environment, as the athletes are managing training and playing at the local club level, and also work and study commitments, nutrition guidance for health, performance and recovery is paramount. Academy rugby athletes will require specific guidance due to the high training volumes, specificity around body composition, and on-pitch demands. Furthermore, food availability as well as financial and social constraints are likely to influence nutritional timing and food choices.

**Methods:** Fourteen provincial academy rugby athletes (age: 20.3 ± 1.5 y; body mass: 104.2 ± 17.2 kg; height: 186.3 ± 9.0 cm) from a single academy engaged in a 4-week nutrition support programme; a certified nutritionist led the programme to provide the nutrition support. The programme was part of a larger project aimed at improving basic dietary practices in the target population and consisted of a single 60-minute education group seminar, individual 15-minute consultations, regular cellular and in-person contact and provision of educational pamphlets detailing how to structure and cook appropriate meals. Nutrition knowledge was measured pre- and post-intervention using the validated Abridged-Sports Nutrition Knowledge Questionnaire (ASNKQ) (Trakman et al, 2018).

**Results:** Individual and mean nutrition knowledge scores are displayed in Table 1. No difference was observed between pre-intervention (38.9 ± 14.4%) and post-intervention (42.0 ± 12.6%) ASNKQ scores (p = 0.26). Pre- and post-intervention scores were classified as ‘poor’.

**Table 1.** Individual and average abridged-sports nutrition knowledge questionnaire scores (%).

Participant	Pre-Intervention (%)	Post-Intervention (%)
1	47.1	58.8
2	61.8	52.9
3	38.2	47.1
4	32.4	35.3
5	35.3	32.4
6	55.9	55.9
7	26.5	44.1
8	47.1	55.9
9	55.9	41.2
10	26.5	47.1
11	14.7	14.7
12	20.6	29.4
13	32.4	29.4
14	50.0	44.1
Average ± SD	38.9 ± 14.4	42.0 ± 12.6

**Conclusions:** Nutrition knowledge uptake did not improve in response to the nutrition support programme. Additionally, nutrition knowledge scores of the cohort before and after the programme were classified as ‘poor’ however large inter-individual variation was apparent in the athletes. Particularly at developmental levels, nutrition knowledge may be inadequate. Many factors can negatively influence dietary choices for health and performance and this research highlights the necessity of providing consistent and individualized nutritional support and education to athletes.

**Exercise and Dietary Factors on the Microbiome of Teenage Cross-Country Runners**

Carla Duenas^a^, Luciana Perasso^a^, and Joshua Costin^a^

^a^Nova Southeastern University, Fort Lauderdale, FL, USA

Corresponding author: jcostin@nova.edu

**Background:** A variety of factors can impact the gut microbiome, including diet and exercise. Among elite adult runners, exercise alone can induce changes in distinct gut bacteria such as *Veillonella atypica*. Fiber intake has also been positively correlated to levels of Veillonella in the gut. The purpose of this study was to observe if changes in *V. atypica* occur in a group of non-elite male cross-country runners, as well as to determine the influence of fiber intake on the Veillonella composition in their gut.

**Methods:** This is an IRB-approved mixed methods study with both survey and interventional components. A total of 28 male runners were recruited and asked to provide 3 stool samples, submit daily food logs as well as complete surveys throughout a 10-day training period. Thryve Inc. provided the shotgun metagenomic sequencing in which bacterial diversity and richness was analyzed from the stool samples.

**Results:** Student athletes were highly active, running daily at a competitive pace. Despite this level of activity, only one student athlete experienced exercise associated increases in *V. atypica* in his gut microbiome at levels consistent with elite adult marathon runners. After analyzing the fiber intake of the study population, it was observed that students were remarkably consistent in their daily fiber intake across the course of the study. Those athletes that consumed over the USRDA recommended daily intake of 25 g per day had statistically significant increases in the level of Veillonella in their gut.

**Conclusions:** Most teenage cross-country runners were not observed to have any significant levels of *V. atypica*, however one subject did have a marked increase in *V. atypica* following a week of intensive running, similar to the increase observed in elite marathon runners. It is unclear if this runner represents a more mature gut microbiome, more intensive training than his peers, or represents some combination of the two that enriches for V. typica. More research will need to be performed to distinguish between these possibilities. Runners that consumed the USRDA of at least 25 g of fiber per day were associated with significant increases in counts of Veillonella throughout the study period. SCFAs produced by Veillonella bacteria produce effects in the body that may increase athletic performance. This research suggests potential areas of research and product development for next generation probiotics, prebiotics and/or synbiotics that can possibly offer performance-enhancing effects.

**Acknowledgments:** This research was sponsored by Nova Southeastern University’s HPD Educational Research Grant.

**The Effect of Consuming Water and a Protein Shake on Body Composition Measures on an InBody270**

Samantha Alles^a^, Jose Antonio^a^, Lia Jiannine^a^

^a^Department of Health and Human Performance, Fight Science Laboratory, Nova Southeastern University, Davie, FL, USA

Corresponding author: jose.antonio@nova.edu

**Background:** The purpose of this study was to examine the effects of consuming either 20oz of water or 20oz of isovolumic protein shake (160 kcal, 3 g fat, 4 g carbohydrate, 30 g protein) on indices of body composition. Body Impedance Analysis (BIA) is an inexpensive, noninvasive, and readily adaptable test. The InBody270 is an electric circuit in hand‐to‐foot BIA extends from wrist to ankle, measuring the resistance through arm, trunk and leg allowing for the assessment of the gut, trunk, upper and lower body. There have been previous studies on the influence of food and hydration on the hand-to-hand and foot-to-foot method however, there has been a dearth of research that consider the effect of immediate fluid consumption on measurements with hand‐to‐foot BIA. Therefore, the aim of the present study was to assess if water and protein alone can affect hydration levels, potentially skewing the results of biological impedance analysis.

**Methods:** Forty-two recreationally active men (n = 13) and women (n = 29) (mean±SD – 168 ±10 cm, 22 ±5 yr, 69.8 ±11.2 kg) voided their bladder, completed a baseline BIA, and then consumed 20oz of water or a protein shake in a randomized, crossover study. Body composition was assessed via multi-frequency bioelectrical impedance (InBody270) at baseline, immediately post-consumption (0 minutes), 30 minutes post-consumption, and 60 minutes post-consumption.

**Results:** There were no significant differences in body mass, lean body mass, and fat mass body mass between the water and protein groups. In both water and protein groups, percent body fat was significantly greater (p <0.0001) at time points 0, 30 and 60 minutes compared to baseline.

**Conclusions:** The acute consumption of either water or an isovolumic protein shake resulted in a measurable increase in percent body fat immediately post-consumption as well as 30 and 60 minutes thereafter.

**Cardiovascular health risk of collegiate Division 1 American style football athletes**

Ashley Ring^a^, Sophie Pomrehn^b^, Nadine Mikati^a^

^a^Nova Southeastern University, Davie, FL USA; ^b^University of Wisconsin Madison, Madison, WI USA

Corresponding author: ar3041@mynsu.nova.edu

**Background:** Cardiovascular disease (CVD) is considered the leading cause of death around the world, with risk factors including obesity and hypertension.1,2 In regards to cardiovascular health, American-style football (ASF) athletes have deliberate weight gain, psychosocial stress, large amounts of static hemodynamic stress, low aerobic conditioning, and routine nonsteroidal anti-inflammatory drug use.3 Collegiate ASF players make up a unique subset of the population that could be at an increased risk of CVD.4 As early as high school, ASF athletes are showing increased CVD risk factors such as dyslipidemia.5 It has also been shown that former athletes who played with a BMI over 30 have a 50% increase in cardiovascular related deaths when compared to healthy controls.3,6 This cross-sectional study aimed to evaluate the presence of cardiovascular risk factors in a Division 1 ASF team.

**Methods:** The researchers analyzed the prevalence of CVD risk factors of a Collegiate Division12020Varsity Football Team through measuring body mass index (BMI), waist circumference (WC), systolic blood pressure (SBP), diastolic blood pressure (DBP), and body fat percentage (BF%). The measurements of the team as a whole (n = 38) were taken and then compared the data to validated cutoff measurements for obesity and hypertension (Table 1). Additionally, the participants were split into linemen (n = 14) and non-linemen (n = 24) groups and compared using an independent t-test.

**Results:** The overall team analysis showed all the mean measurements fell below the validated cutoffs of 102 cm, 25%, 30 kg/m2, 130 mm Hg, and 80 mm Hg respectively (Table 1). In the linemen group, WC, BF%, SBP and BMI either meet or exceed the validated cutoffs, while DBP was below its cutoff (Table 2). In the non-linemen group, all measurements fell below the validated cutoffs (Table 2). When comparing the two groups, WC, BF%, and BMI measurements of the linemen were significantly higher than the non-linemen (p <0.001 for each) (Figures 1-3), while there was no significant difference between the linemen and non-linemen measurements (p = 0.754 and p = 0.927 respectively) (Figures 4-5).

**Table 1.** Average measurements of all the study participants (n = 38) for waist circumference (WC), body fat percentage, systolic blood pressure (SBP), diastolic blood pressure (DBP), and body mass index (BMI) along with their validated cutoff measurements for obesity and hypertension.

Measurement	Mean	Validated Cutoffs
WC (cm)	95.49 ± 9.86	≥102
Body Fat (%)	21.72 ± 6.03	> 25
SBP (mm Hg)	129.4 ± 9.32	≥ 130
DBP (mm Hg)	75.08 ± 6.86	≥ 80
BMI (kg/m2)	24.66 ± 4.50	≥ 30

**Figure 1.**
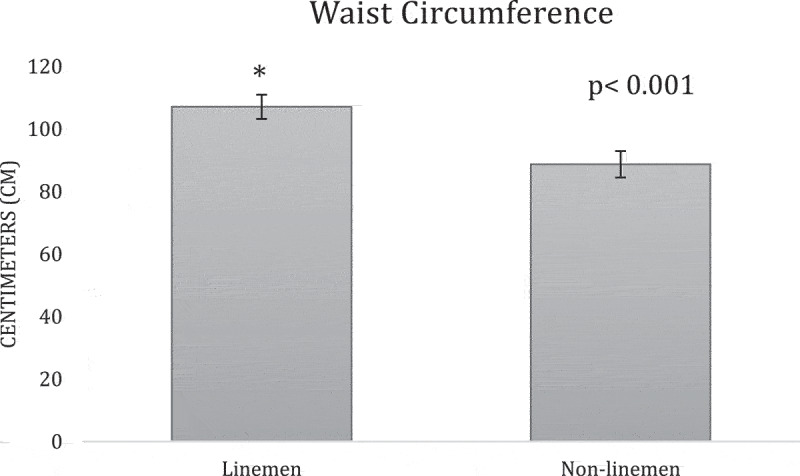
Waist circumference (WC) results from the independent t-test comparing the means of the linemen and non-linemen groups. The linemen group had significantly higher WC than the non-linemen group (p <0.001*; * = significant difference). Significance is defined as p <0.05. The error bars represent standard deviation.

**Figure 2.**
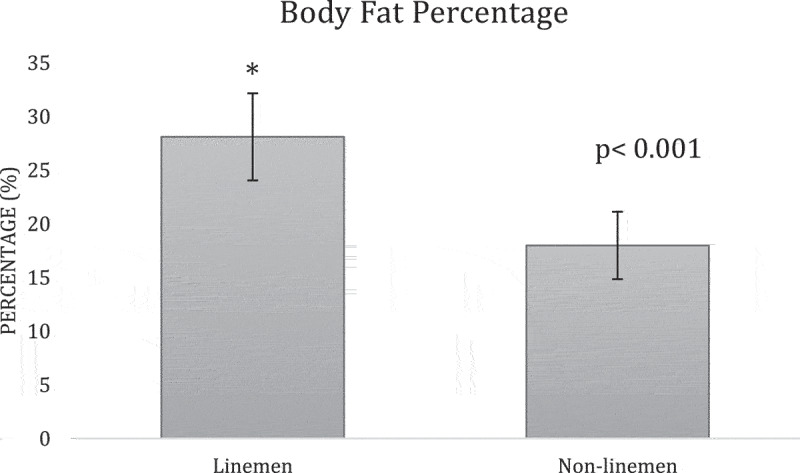
Body fat percentage results from the independent t-test comparing the means of the linemen and non-linemen groups. The linemen group had a significantly higher body fat percentage than the non-linemen group (p <0.001*; * = significant difference) Significance is defined as p <0.05. The error bars represent standard deviation.

**Figure 3.**
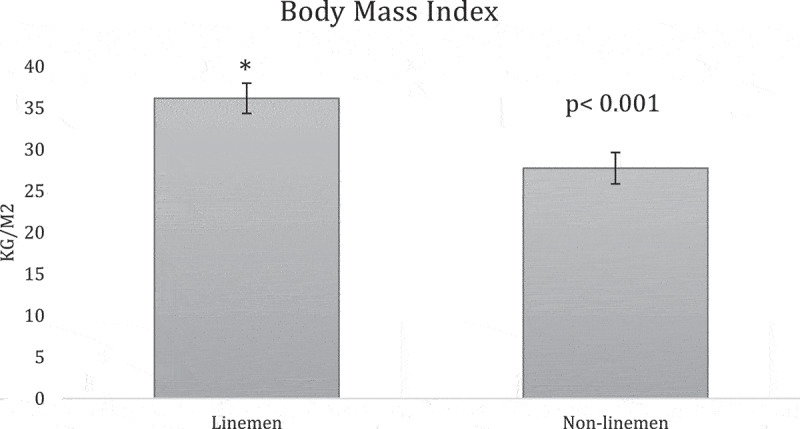
Body mass index (BMI) results from the independent t-test comparing the means of the linemen and non-linemen groups. The linemen group had significantly higher BMI than the non-linemen group (p <0.001*; * = significant difference). Significance is defined as p <0.05. The error bars represent standard deviation.

**Figure 4.**
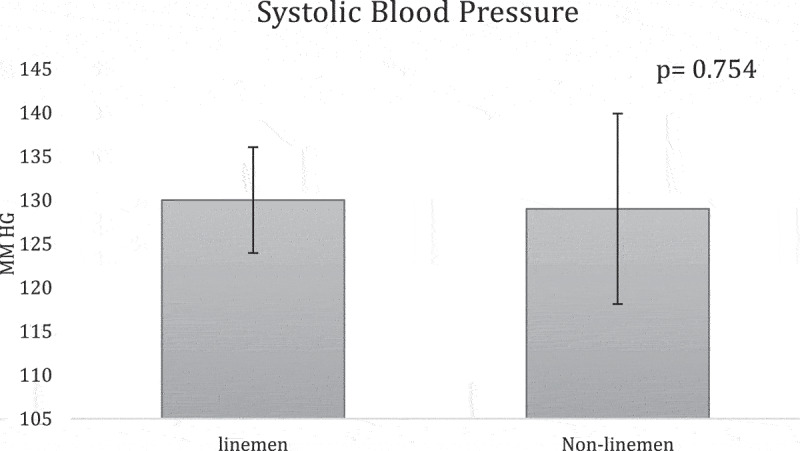
Systolic blood pressure (SBP) results from the independent t-test comparing the means of the linemen and non-linemen groups. There was no significant difference in SBP between the two groups (p = 0.754). Significance is defined as p <0.05. The error bars represent standard deviation.

**Figure 5.**
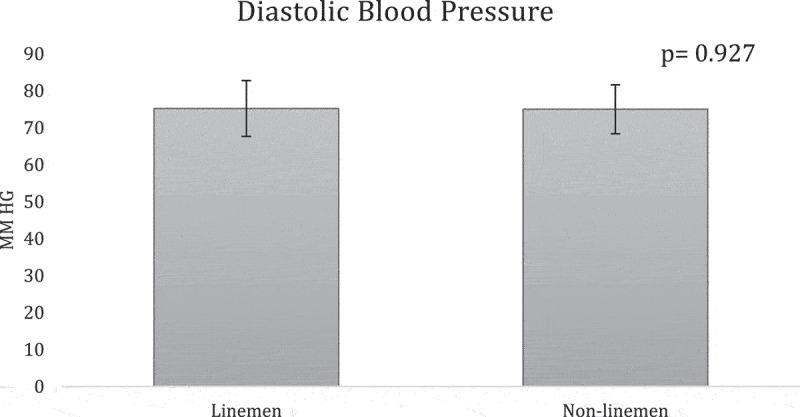
Diastolic blood pressure (DBP) results from the independent t-test comparing the means of the linemen and non-linemen groups. There was no significant difference in DBP between the two groups (p = 0.927). Significance is defined as p <0.05. The error bars represent standard deviation.

**Figure 1.**
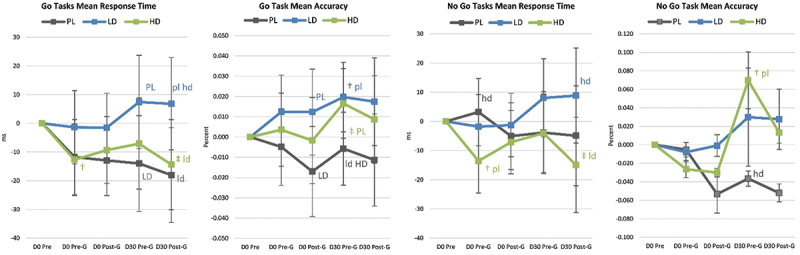
Changer in Go/NoGo test results. Data are means and 95% confidence intervals. Changes from baseline are shown as † (p<0.05 change from baseline) and ‡ (p<0.05 to p<0.10 trends from baseline). Small case letters indicate p<0.05 differences from placebo (pl), low dose (ld), or high dose (hl) whileupper-case letters (PL, LD, HD) indicate trends (p<0.0-5 to p<01.10).

**Table 2.** Average measurements collected for waist circumference (WC), body fat percentage, systolic blood pressure (SBP), diastolic blood pressure (DBP), and body mass index (BMI) of the linemen and non-linemen groups were compared to the validated cutoff measurements for obesity and hypertension. Measurements were compared using an independent t-test. The * indicates a significant difference between the linemen and non-linemen groups. Significance is defined as p <0.05.

Measurement	Linemen	Non-linemen	Two-Tailed P-Value	Validated Cutoffs
WC (cm)	107.1 ± 3.89	88.72 ± 4.22	<0.001*	≥102
Body Fat (%)	28.11 ±4.06	17.99 ± 3.14	<0.001*	> 25
SBP (mm Hg)	130.0 ± 6.05	129.0 ± 10.89	0.754	≥ 130
DBP (mm Hg)	75.21 ± 7.53	75.00 ± 6.60	0.927	≥ 80
BMI (kg/m2)	36.15 ± 1.82	27.74 ± 1.89	<0.001*	≥ 30

**Conclusions:** The study found that while the team as whole did not currently present an increased risk for CVD, the linemen present with more CVD risk factors than the non-linemen players. This information can be used to improve the pre-participation physical and health monitoring of the athletes as well as identify individuals who may benefit from education on CVD and lifestyle changes to help to prevent its development.

**Acknowledgments:** Dr. Doug Kalman, Stephanie Petrosky, Nova Southeastern University Department of Nutrition. The authors would like acknowledge the Sports Medicine staff at the University of Wisconsin-Madison Department of Intercollegiate Athletics for their commitment to the welfare of the student-athletes and contributions to the Badger Athletic Performance program.

**References:** (1) Cardiovascular diseases (CVDs). World Health Organization. https://www.who.int/news-room/fact-sheets/detail/cardiovascular-diseases-(cvds). Accessed 1 June 2020.
(2) Francula-Zaninovic S, Nola IA. Management of Measurable Variable Cardiovascular Disease’ Risk Factors. Curr Cardiol Rev. 2018;14(3):153-163. doi:10.2174/1573403X14666180222102312.
(3) Kim J.H., Zafonte R., Pascuale-Leon A., Nadler L.M., Weisskopf M., Speizer F.E., Taylor H.A., Baggish A.L. American-Style Football and Cardiovascular Health. Journal of the American Heart Association. 2018; 7(8). doi: 10.1161/JAHA.118.008620
(4) Dixit S, Hecht S, Concoff A. Cardiovascular Risk Factors in Football Players. Current Sports Medicine Reports. 2011; 10(6): 378-382. DOI: 10.1249/JSR.0b13e31823a362e.
(5) Oliver JM, Jouber DP, Caldwell A, Martin SE, Crouse SF. A longitudinal study examining the effects of a season of American football on lipids and lipoproteins. Lipids in Health and Disease. 2015; 14:35. DOI:10.1186/s12944-15-0021-6.
(6) Anding R, Oliver JM. Football Player Body Composition: Importance of Monitoring for Performance and Health. https://www.gssiweb.org/sports-science-exchange/article/sse-145-football-player-body-composition-importance-of-monitoring-for-performance-and-health. Published 2015. Accessed 19 June 2020.

**Efficacy of a Microalgae Extract Combined with Natural Guarana on Cognitive Performance of Gamers III: Go/No-Go Test**

Broderick Dickerson^a^, Megan Leonard^a^, Jonathan Maury^b^, Drew Gonzalez^a^, Jacob Kendra^a^, Tori Jenkins^a^, Kay Nottingham^a^, Choongsung Yoo^a^, Dante Xing^a^, Joungbo Ko^a^, Rémi Pradelles^b^, Ryan Sowinski^a^, Christopher J. Rasmussen^a^, Richard B. Kreider, FISSN^a*^

^a^Exercise & Sport Nutrition Laboratory, Human Clinical Research Facility, Texas A&M University, College Station, TX, USA; ^b^Microphyt, Research & Development Department, Baillargues, Mudaison, FR

Corresponding author: rbkreider@tamu.edu

**Background:** Competitive gaming requires visual selective attention, short-term memory or task switching, and an ability to sustain a high level of energy over time. Fucoxanthin is a major carotenoid, found in specific microalgae varieties like Phaeodactylum tricornutum that has been reported to possess neuroprotective and nootropic effects through its anti-inflammatory and antioxidant activities on different signaling pathways like Nrf2-ARE. The purpose of this study was to evaluate whether acute and 30-day supplementation of a microalgae extract from Phaeodactylum tricornutum with Guarana would affect cognitive function of gamers.

**Methods:** In a double-blind, placebo-controlled manner, 51 male and 10 female experienced gamers (21.7 ±4 years, 73.0 ±13 kg, 24.2 ±3.6 kg/m2) were randomly assigned to ingest a placebo (PL); low-dose (LD) of GamePhyt™ (MicroPhyt, Baillargues, FR) containing 440 mg/day of Phaeodactylum tricornutum extract including 1% Fucoxanthin + 440 mg/day of guarana, or high-dose (HD) of GamePhyt™ containing 2 × 440 mg/day of Phaeodactylum tricornutum extract including 1% Fucoxanthin + 440 mg/day of guarana for 30-days. Participants refrained from consuming atypical amounts of stimulants, food, and supplements that may affect cognition during the study. Acute (single dose) cognitive function tests were administered on Day 0 prior to supplementation, 15-min post-supplementation, and after the participants played their most competitive video game for 60-minutes. Participants continued supplementation for 30-days and then repeated pre-supplementation and post-gaming cognitive function tests. The battery of cognitive function tests included the Go/No-Go (GNG) which assesses sustained attention and response control through reaction time and accuracy of responding to visual stimuli (i.e. seeing P or R) by either pressing a key representing ‘Go’ or inhibiting a response by not pressing the key representing ‘No-Go’. Data were analyzed by General Linear Model (GLM) univariate analyses with repeated measures using weight as a covariate and mean and percent changes from baseline with 95% confidence intervals.

**Results:** Figure 1 presents selected analyses performed on Go/No-Go data. Results revealed evidence that acute LD and/or HD ingestion significantly reduced pre-game (Pre-G) No-Go Tasks Round 2 Condition P and Mean response times compared to PL. Post-game No-Go Tasks Round 2 Condition P responses were also lower than baseline in the HD group. Post-game (Post-G) Go Task Mean Accuracy and Round 1 Condition P response time were also faster in the HD group compared to PL. After 30-days of supplementation, Pre-game Go Tasks Mean Accuracy and No-Go Task Round 1 Condition R Accuracy were higher with LD and/or HD. The HD group appeared to have more favorable response times than the LD group.

**Figure 1.**
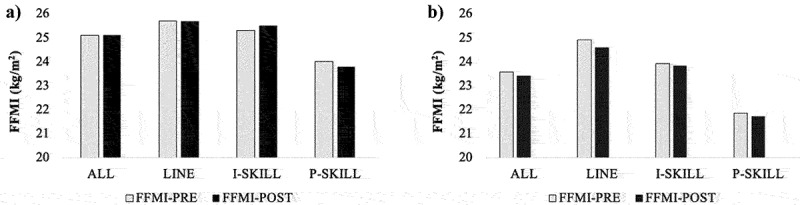
Positional and seasonal changes in A) Division II and B) Division III football athletes.

**Conclusions:** Results provide some evidence that acute and chronic supplementation with a microalgae extract from Phaeodactylum tricornutum with Guarana can affect sustained attention and response control through reaction time and accuracy of responding to visual stimuli. Responses were generally improved to a greater degree in the HD group.

**Acknowledgments:** This study was funded by MicroPhyt (Baillargues, FR) as a fee-for-service project to the Human Clinical Research Facility at Texas A&M University and conducted by the Exercise & Sport Nutrition Lab.

**Effects of Carbohydrate + Caffeine Beverage on Game Performance, Blood Glucose, and Perceived Effort in Collegiate Soccer Players**

Andrew R. Jagim^a,b,e^, Abby Ambrosius^b^, Makenna Carpenter^b^, Chinguun Khurelbaatar^b^, Jacob Pyykkonen^b^, Mia Khalil^b^, Joesi Krieger^c^, Lochlan Charley^c^, Chad M. Kerksick^c,a^, Jennifer B Fields^d,e^ and Margaret T Jones^e,f^

^a^Sports Medicine, Mayo Clinic Health System, Onalaska, WI, USA; ^b^Exercise & Sport Science, University of Wisconsin – La Crosse, La Crosse, WI, USA; ^c^Exercise & Performance Nutrition Laboratory, Lindenwood University, St. Louis, MO, USA; ^d^Exercise Science and Athletic Training, Springfield College, Springfield, MA, USA; ^e^Frank Pettrone Center for Sports Performance, George Mason University, Fairfax, VA, USA, ^f^Sport, Recreation, and Tourism Management, George Mason University, Fairfax, VA, USA

Corresponding author: jagim.andrew@mayo.edu

**Background:** Carbohydrate availability and hydration status have been shown to influence exercise and sports performance. Caffeine ingestion has also been shown to elicit improvements in performance. However, less is known relative to the combined effects of these nutrients, particularly during sport-specific activities. The purpose of the study was to examine the effect of carbohydrate + caffeine beverage on game performance, blood glucose, and perceived effort in collegiate soccer players.

**Methods:** Forty-three collegiate women soccer athletes (Age: 20.3 ± 1.0 yrs.; Height: 167.5 ± 6.3 cm; Weight: 65.4 ± 8.6 kg; Bodyfat %: 22.3 ± 6.4 %) were recruited to participate in a study consisting of a single day of scrimmage play, in which four separate teams played once. Athletes consumed either a carbohydrate + caffeine (CARB) beverage or a control (PLA) beverage (flavored water) in a double-blind, randomized control trial design. Beverage ingestion occurred during half-time of each scrimmage. Prior to and after each game, body weight, ratings of perceived exertion (RPE), and blood glucose levels were measured. Athletes were equipped with a GPS monitoring system with an integrated accelerometer and heart rate, to assess heart rate, training impulse (TRIMP), total distance covered, high speed distance, and velocity.

**Results:** Blood glucose levels after the scrimmage were positively associated with total distance (r = 0.434; p = 0.01), distance per minute (r = 0.439; p <0.01), average velocity (r = 0.438; p = 0.01); TRIMP (r = 0.404; p = 0.018), and average heart rate (r = 0.428; p = 0.12) during the second half. There was a significant main effect for half regarding blood glucose (p <0.001), total distance (p <0.001), high speed distance (p <0.001), and TRIMP (p = 0.046). There was a significant half x condition effect for blood glucose (p = 0.05). Pairwise comparisons indicated the CARB condition resulted in 27 mg/DL difference compared to PLA following the 2nd Half. There were no significant differences between conditions for any of the remaining parameters (Table 1).

**Table 1.** Changes in soccer performance across each half, grouped by condition.

Measure	Condition	1st Half	2nd Half	ES
Distance (m)	PLA CARB	2248 ± 161 2315 ± 166	1878 ± 145 1952 ± 150	2.4 2.3
HSD (m)	PLA CARB	326 ± 35 307 ± 36	229 ± 27 207 ± 28	3.1 3.1
Velocity (km/hr)	PLA CARB	4.7 ± 0.3 4.9 ± 0.3	3.8 ± 0.3 4.0 ± 0.3	3.0 3.0
Avg. HR (bpm)	PLA CARB	160 ± 4.9 159 ± 5.1	153 ± 4.8 154 ± 4.9	1.4 0.9
TRIMP (AU)	PLA CARB	73 ± 6.3 71 ± 6.5	65 ± 5.7 64 ± 5.9	1.3 1.1

PLA = Placebo condition; CARB = Carbohydrate + caffeine condition; HSD = high speed distance; TRIMP = training impulse; km – kilometers; bpm = beats per minute; ES = effect size. *Denotes statistical significance.

**Conclusions:** Consumption of a carbohydrate + caffeine beverage during half-time resulted in higher blood glucose levels post-game compared to placebo. However, the experimental beverage did not influence total distance covered, average velocities, average heart rate or training impulse during the second half of play. Higher blood glucose values post game were associated with greater total distance covered, greater distances covered per minute, average velocity, training impulse and average heart rate during the second half.

**Acknowledgments:** This study was supported by an internal research grant on behalf of the College of Science and Health at the University of Wisconsin – La Crosse. A special recognition to Suppz (La Crosse, WI) for providing shaker cups to all study participants.

**The Efficacy of Oligonol® for Middle-aged Marathon Athlete’s Performance**

Takuma Shio^a^, Yukiko Fukuchi^a^, Ken Ishii^b^, Jun Takanari^a^

^a^Amino Up Co., Ltd., 363-32 Shin-ei, Kiyota-ku, Sapporo 004-0839, Japan, Asia; ^b^General Incorporated Association Sapporo EXCEL Athlete Club, 5jo-3-12-18-508, Kitago, Shiroishi-ku, Sapporo 003-0835, Japan, Asia

Corresponding author: yumi.masaki@maypro.com

**Background:** As it is known that regular physical activity is associated with an increase of life expectancy, people are trying to keep physical exercise, globally. Whereas WHO recommends at least 150-300 minutes of moderate-intensity aerobic physical activity weekly, it is crucial for people that the fatigue is not accumulated, performance improvement and physical change are tangible to keep a motivation to continue exercise. Oligonol®, a standardized, oligomerized polyphenol from Litchi chinensis fruit extract (Amino Up Co., Ltd., Japan) has been reported to be beneficial for our health in various applications, including cardiovascular health, athletics, skin beauty from within, and body composition improvement. Especially, athletic benefits have been clinically validated in terms of stress, inflammation, and fatigue, caused by exercise load. In this study, we evaluated the effects of Oligonol on exercise functions of middle-aged long-distance runners.

**Methods:** Thirty-five middle-aged long-distance runners were recruited in this study, and they took Oligonol (200 mg/day) or Placebo for 60 days. Body compositions, physical performance in a 12-minute incremental exercise test, and subjective exercise strength (Rate of Perceived Exertion: RPE) were measured and evaluated before and after intake of Oligonol or Placebo.

**Results:** In the evaluation of body composition, body fat percentage was significantly increased in Placebo group and remained unchanged in Oligonol group. On the other hand, there was no difference in body weight or BMI between the two groups. In the incremental exercise test, Oligonol group showed a significant increase in running distance after the intake compared to before. The RPE-score was significantly decreased in Oligonol group compared to Placebo group.

**Conclusions:** It was suggested that intake of Oligonol in middle-aged long-distance runners suppressed subjective suffering and fatigue immediately after high-intensity exercise and contributed to the improvement of exercise performance.

**Body Composition and Performance Changes in Elite College Football Players Preparing for the NFL Combine / Draft at The House of Athlete**

Chris Horn^a^, Veronica Mekhail^b^, Cassandra Evans^b,c^, Flavia Rusterholz^b^, Ian McQuate^a^, Troy Wells^a^, Elena Rodriguez^a^, Brandon Marshall^a^, Jose Antonio PhD^b^

^a^The House of Athlete, Weston, FL, USA; ^b^Exercise and Sport Science, Fight Science Laboratory, Nova Southeastern University Florida, Davie, FL, USA; ^c^Health Sciences, Rocky Mountain University of Health Professions, Provo, UT, USA

Corresponding author: jose.antonio@nova.edu

**Background:** This study was designed to examine body composition and performance changes in elite college football players participating in The House of Athlete’s NFL Combine/2022 draft preparation program. The NFL Combine is an opportunity for collegiate American Football players to showcase their skills for the NFL draft. These metrics are analyzed by NFL scouts to determine an athlete’s draft value and predicted success in the NFL.

**Methods:** Twenty healthy male Division 1 (D1) college football players aged 22 ± 1 y participated in a 7-week training program that included six days per week of weight training, field session position work, and recovery treatment. In addition to all meals and supplements were provided on site. The pre- and posttesting period was approximately from January to March (i.e. at the NFL Combine). Seven of the twenty football players completed a pre and post body composition assessment via the DEXA. The physical performance measures included the vertical jump, broad jump, and bench press. All 20 players completed the vertical jump, 17 completed the broad jump, and 11 completed the bench press. A pair t-test was used to determine if pre- vs. posttest differences existed. Imperial units are used in the NFL and thus this how our data will be presented.

**Results:** Athletes that participated in the HOA training program on average showed significant improvements in all metrics from vertical jump, broad jump, bench press and body composition. Significant improvements included (mean±SD): vertical jump – pre 30.3, post 33 inches (p = 0.004), broad jump – pre 111.9, post 119.6 inches (p = 0.0001), bench press – pre 10.0 reps, post 17.7 reps (p = 0.0001), fat free mass – pre 158.4, post 164.13 lb (p = 0.0026) and fat mass -pre 35.14, post 33.44 lb (p = 0.0188).

**Conclusions:** There were significant improvements in body composition and physical performance after a 7-week training program in this group of D1 college football players.

**Seasonal Changes by Position in Fat-free Mass Index in Collegiate American Football Players**

Jennifer B. Fields^a,b^, Maddy Dunne^a^, Adam Feit^a^, Kyle Beyer^c^, Meghan K. Magee^b,d^, Margaret T. Jones^b,e^, Andrew R. Jagim^b,f^

^a^Exercise Science and Athletic Training, Springfield College, Springfield, MA, USA; ^b^Patriot Performance Laboratory, Frank Pettrone Center for Sports Performance, George Mason University, Fairfax, VA, USA; ^c^Exercise Science, Bloomsburg University, Bloomsburg, PA, USA; ^d^Kinesiology, George Mason University, Manassas, VA, USA; ^e^Sport, Recreation, and Tourism Management, George Mason University, Fairfax, VA, USA; ^f^Sports Medicine Department, Mayo Clinic Health System, La Crosse, WI, USA

Corresponding author: jfields2@springfieldcollege.edu

**Background:** Optimal health is a precondition for top sport performance, and body composition plays a critical role in athlete health. Generally, high levels of fat-free mass (FFM) are favorable for athletes and have been related to vertical jump performance, sprint time, relative power, and maximal strength. However, fat free mass index (FFMI), which includes adjustments for height, might offer a better representation. As FFMI is understudied relative to sport, the purpose of this study was to (1) provide descriptive FFMI data by position in collegiate American football athletes; and (2) examine seasonal changes in FFMI.

**Methods:** National Collegiate Athletic Association (NCAA) Division (D) II (n = 33; age: 21.3 ± 1.6 yrs; height: 182.5 ± 7.5 cm; body mass: 102 ± 19 kg; body fat %: 16.9 ± 7.9%; FFM: 83.7 ± 9.4 kg) and D-III (n = 111; age: 19.5 ± 1.2 yrs; height: 181.0 ± 5.9 cm; body fat %: 16.6 ± 6.5%; FFM; 77.0 ± 8.2 kg) American football players underwent body composition assessment via bioelectrical impedance analysis during pre-season (week 1) and post-season (week 12). FFMI was calculated from dividing FFM by height squared, and was also adjusted to height via linear regression. The slope of a regression line was used to adjust raw FFMI values based on the average heights in the sample of D-II (183 cm) and D-III (181 cm) athletes and the following height-adjusted FFMI values were calculated as follows: FFMI + [slope x (average height – subject height)]. A paired samples t-test indicated no difference between FFMIraw and FFMIadj (p = 0.89, p = 0.67, respectively); therefore, FFMIraw was used in subsequent analysis. Separate one-way analysis of variance with Bonferroni post-hoc tests were conducted to evaluate differences in FFMI by sport-position (lineman (LINE: offensive and defensive line), interior skill (I-SKILL: running back, linebacker, tight end, quarterback, full back) and perimeter skill (P-SKILL: wide receiver, defensive back)). A paired samples t-test was used to determine changes in FFMI from pre- to post-season season (p <0.05).

**Results:** FFMI values for D-II and D-III football players averaged 25.1 ± 1.9 kg/m2 (range: 20.1 – 30.1 kg/m2) and 23.5 ± 2.0 kg/m2 kg/m2 (range: 18.0 – 27.8 kg/m2), respectively. No differences in average FFMI were observed among position groups in D-II players (LINE: 25.7 ± 1.8 kg/m2; I-SKILL: 25.5 ± 1.9 kg/m2; P-SKILL: 23.9 ± 1.5 kg/m2, p = 0.061). However, positional differences were apparent in D-III players (p <0.001), with highest values observed in LINE (24.8 ± 1.5 kg/m2) and lowest values in P-SKILL (21.8 ± 1.0 kg/m2, p <0.001). FFMI remained unchanged for all positions from pre- to post-season in D-II (p = 0.891) and D-III (p = 0.285) players (Figure 1). Of interest, 57% of D-II and 26% of D-III athletes surpassed previously published research suggesting a natural upper limit of 25.0 kg/m2 in male athletes.

**Conclusions:** The current results suggest that 25 kg/m2 underestimates the natural FFMI limit in collegiate American football players. Mean FFMI differed significantly among position groups at the D-III level, which likely reflects unique physical and training demands of each position. FFMI can serve as a noninvasive and valuable tool for assessment of athletic potential, guidance for body composition goals, and development of training and nutritional programs.

**Anthocyanin-rich New Zealand Blackcurrant Extract Reduces Running-induced Gastro-Intestinal Symptoms in the Heat**

Mark ET Willems^a^, Ania M Hiles^a,†^, Tessa R Flood^a^, Lucy EV Wheeler^a^, Rianne Costello^a^, Ella F Walker^a^, Kimberly M Ashdown^a^, Matthew R Kuennen^c^, Ben J Lee^a,d^

^a^University of Chichester, Chichester, UK; ^b^Oxford Brooks University, Oxford, UK; ^c^High Point University, North Carolina, USA; dCoventry University, Coventry, UK

Corresponding author: m.willems@chi.ac.uk

**Background:** Gastrointestinal (GI) distress symptoms are a common running-induced experience for athletes training and competing in hot environmental conditions. GI distress symptoms may compromise exercise performance as well as carbohydrate digestion and absorption. Food components may affect the presence and severity of GI distress symptoms during running in hot environmental conditions. We examined the effect of anthocyanin-rich New Zealand blackcurrant (NZBC) extract on the GI distress symptoms during running in hot environmental conditions.

**Methods:** Recreationally active males (n = 12, age: 28 ±6 yr, BMI: 24.5 ±1.8 kg·m−2, $\dot{V}$ O2max: 56 ±6 mL·kg−1·min−1) volunteered. The study had a placebo-controlled, double blind, randomized, cross-over design. In thermoneutral conditions (18°C and 40% relative humidity), participants completed an incremental exercise test to exhaustion to standardize running intensity (visit 1) and a familiarization (visit 2). Participants dosed with 7-days of NZBC extract (210 mg anthocyanins per day) or placebo. Euhydration was confirmed before the experimental visits of treadmill running for 1 hr at 65% $\dot{V}$ O2max in an environmental chamber (TISS Services UK, Medtead, Hampshire, UK, 34.1 ±0.1 °C, 40.8 ±0.2% relative humidity). At 0, 30 and 60 min during the running and at 60 min following recovery in thermoneutral conditions, GI distress symptoms (i.e. upper, lower and other) were recorded with a modified visual analogue scale (doi: 10.1123/ijsnem.2018-0215). Water was available ad libitum.

**Results:** In the placebo condition, only 2 participants (17%) reported severe symptoms. One participant reported severe dizziness and nausea in the placebo and NZBC extract condition. In the placebo condition, 11 participants (92%) reported total GI symptoms (e.g. belching, heart burn), and this was reduced to 4 participants (25%) with NZBC extract. Only one participant reported belching, nausea and stitch 60 min following recovery in the placebo condition. Upper and lower GI distress symptoms were reduced (upper, placebo: 75%, NZBC: 25% of participants; lower: placebo: 25%, NZBC extract: 17% of participants). Other GI symptoms (i.e. nausea, dizziness and stitch) were also reduced (placebo: 50%, NZBC: 25% of participants).

**Conclusions:** Seven days intake of anthocyanin-rich NZBC extract reduced the incidence of GI distress symptoms during one-hour of treadmill running in hot environmental conditions. For most participants, the severity of GI distress symptoms pre-supplementation was considered very mild. Future research should examine the effects of NZBC extract on running with duration and intensity in conditions for which GI distress symptoms are known to be severe.

**Acknowledgments:** Supplementation was provided by Health Currancy Ltd (United Kingdom) and CurraNZ Ltd (New Zealand). Financial support for conference attendance was obtained from Blackcurrant New Zealand Inc (New Zealand).

**Anthocyanin-rich New Zealand Blackcurrant Extract Enhances Running-induced Fat Oxidation in an Ultra-endurance Amateur Male Runner: A case study**

Mark ET Willems^a^, Andrew R Briggs^a^

^a^Institute of Sport, Nursing and Allied Health, University of Chichester, Chichester, United Kingdom

Corresponding author: m.willems@chi.ac.uk

**Background:** Physical training for ultra-endurance running provides physiological adaptations for exercise-induced substrate oxidation. Previous studies have shown enhanced exercise-induced fat oxidation with intake of New Zealand blackcurrant (NZBC) extract, i.e. in recreationally active males during 30-min walking at 5-MET and endurance trained males during 120-min of cycling at 65% $\dot{V}$ O2max. We examined primarily the effects of anthocyanin-rich NZBC extract on the running-induced metabolic responses in a male amateur ultra-endurance runner.

**Methods:** One amateur male ultra-endurance runner volunteered (age: 40 yr, body mass: 65.9 kg, BMI: 23.1 kg·m−2, body fat: 14.7%, $\dot{V}$ O2max: 55.3 mL·kg−1·min−1, resting heart rate: 45 beats·min−1, running history: 6 years, marathons: 20, ultra-marathons: 28, weekly training distance: ~80 km, weekly running time: ~ 9 hours). Indirect calorimetry (Douglas bags) was used and heart rate recorded at 15-min intervals during 120-min of treadmill running (speed: 10.5 km·hr−1, 58% $\dot{V}$ O2max) in an environmental chamber (temperature: 26°C, relative humidity: ~70%) at baseline and following 7-days intake of NZBC extract (210 mg of anthocyanins per day) with monitoring of core temperature. The male runner had unlimited access to water and consumed a 100-kcal energy gel at 40- and 80-min. Testing was in between participation in 100 mile running events. Substrate oxidation was calculated with corrections for inspiratory oxygen and carbon dioxide fractions.

**Results:** Water intake was 518 and 464 ml in control and NZBC extract conditions. There were no differences (mean of 8, 15-min measurements) for minute ventilation (control: 48.8 ±3.4, NZBC: 49.4 ±3.0 L·min−1), oxygen uptake (control: 2.37 ±0.13, NZBC: 2.37 ±0.11 L·min−1), carbon dioxide production (control: 2.13 ±0.09, NZBC: 2.08 ±0.07 L·min−1) and core temperature (control: 38.2 ±0.2, NZBC: 38.2 ±0.3 °C). With NZBC extract, RER was 0.02 units lower (control: 0.90 ±0.02, NZBC: 0.88 ±0.03, P = 0.006), carbohydrate oxidation was 11% lower (control: 1.94 ±0.12, NZBC: 1.73 ±0.21 g·min−1, P = 0.014), and fat oxidation was 23% higher (control: 0.39 ±0.08, NZBC: 0.48 ±0.12 g·min−1, P = 0.002). Intake of the energy gel did not abolish the NZBC-enhanced fat oxidation.

**Conclusions:** Seven days intake of anthocyanin-rich NZBC extract altered exercise-induced substrate oxidation in a male amateur ultra-endurance runner covering a half-marathon distance in 2 hours. Enhanced running-induced fat oxidation may be beneficial for ultra-endurance running. More observations need to be obtained from male and female ultra-endurance runners to address whether intake of NZBC extract provides a beneficial nutritional ergogenic effect for ultra-endurance athletes to enhance exercise performance.

**Acknowledgments:** Supplementation was provided by Health Currancy Ltd (United Kingdom) and CurraNZ Ltd (New Zealand). Financial support for conference attendance was obtained from Blackcurrant New Zealand Inc (New Zealand).

**No Effect of Anthocyanin-rich New Zealand Blackcurrant Extract on 2000-m Indoor Rowing Performance in Trained Male Rowers**

Mark ET Willems^a^, Stefano Montanari^a^, Tatjana Seymour^a^, Oliver Page^a^

^a^Institute of Sport, Nursing and Allied Health, University of Chichester, Chichester, UK

Corresponding author: m.willems@chi.ac.uk

**Background:** Previous studies have provided observations on performance enhancement by intake of New Zealand blackcurrant (NZBC) extract, e.g. for 16.1 km ergometer cycling and intermittent high-intensity treadmill running. The effects of nutritional ergogenic aids on exercise performance may depend on the recruitment of muscle mass and the exercise-specific metabolic and physiological responses of an exercise modality. We examined the effects of 7-day intake of NZBC extract on 2000-m rowing performance.

**Methods:** Male indoor rowers from University teams (n = 14, age: 21 ±2 years, height: 182 ±8 cm, mass: 81 ±14 kg, BMI: 24.3 ±2.9 kg·m−2, body fat: 14 ±4%, rowing VO2max: 53.7 ±10.2 ml·kg−1·min−1) volunteered. Participants were familiarized with two maximal efforts of 2000-m (drag factor 120, Concept2, Nottingham, United Kingdom). Testing for each participant was at the same time of day (10 were tested in the morning and 4 in the afternoon). Feedback during rowing was distance and stroke rate. Rowing time and stroke rate were recorded every 400-m sector. Heart rate during rowing was measured every 500-m sector in seven participants. Participants consumed capsulated 600 mg of NZBC extract (210 mg of anthocyanin per day) or placebo for 7 days (randomized, cross-over design). The final capsules were taken 2 hours before testing with intake of a slice of toast and water.

**Results:** There was no difference in the rowing time for each of the sectors (e.g. 0-400-m, placebo: 86.0 ±5.7, NZBC: 87.3 ±6.6 s) and total rowing time for the 2000-m between placebo (437.2 ±32.6 s) and NZBC (441.0 ±34.9 s) (P = 0.13). There was no difference for average power output and average heart rate (placebo: 176 ±10, NZBC: 175 ±11 beats·min−1, P = 0.27). With NZBC, the average stroke rate was higher during the 2000-m rowing (placebo: 27 ±2, NZBC extract: 28 ±2 strokes·min−1, P = 0.003). Ten participants (71%) had a higher average stroke rate with intake of NZBC extract.

**Conclusions:** For 2000-m indoor rowing, anthocyanin-rich NZBC extract had no effect on the pacing strategy and the total rowing time. NZBC extract allowed a higher stroke rate. During rowing, stroke rate is linked with breathing frequency. Under the assumption that tidal volume did not change in our study, the increased ventilation with NZBC extract may be beneficial for longer distance rowing events. NZBC extract did not affect 2000 rowing performance in male indoor rowers notwithstanding an increased stroke rate.

**Acknowledgments:** Supplementation was provided by Health Currancy Ltd (United Kingdom) and CurraNZ Ltd (New Zealand). Financial support for conference attendance was obtained from Blackcurrant New Zealand Inc. (New Zealand).

**Anthocyanin-rich New Zealand Blackcurrant Extract Enhances Whole-body Resting Fat Oxidation in Physically Active Males**

Mark ET Willems^a^, Pelin Bilgiç^b^, Stefano Montanari^c,a^, Mehmet A Şahin^a,b^

^a^Institute of Sport, Nursing and Allied Health, University of Chichester, Chichester, United Kingdom; ^b^Department of Nutrition and Dietetics, Hacettepe University, Ankara, Turkey

Corresponding author: m.willems@chi.ac.uk

**Background:** New Zealand blackcurrant extract has been shown to enhance exercise-induced fat oxidation during walking and cycling. We examined the effects of 14-day intake of New Zealand blackcurrant extract on the metabolic and physiological responses during supine rest in males.

**Methods:** Healthy physically active males (n = 16, age: 24 ±6 yr, body mass: 78 ±16 kg, height 178 ±6 cm, BMI: 24.7 ±4.1 kg·m−2 (8 normal weight, 7 overweight, 1 obese), body fat: 15 ±6%) volunteered. Participants were tested at baseline (no supplementation) and after 14-days intake of New Zealand blackcurrant extract in a randomized, crossover design. Two capsules of New Zealand blackcurrant extract (600 mg containing 210 mg of anthocyanins) were consumed every morning with breakfast. The last 2 capsules were taken two hours before the visit with one slice of bread and water 3 hours before the visits. There were no differences for carbohydrate, fat, and protein intake between the visits. Resting expired air was collected for two times for 10 min with Douglas bags and recording of heart rate. Rates of whole-body resting fat and carbohydrate oxidation were calculated. Responses for the 10 min with the lowest minute ventilation were analyzed.

**Results:** During supine rest, there was no effect on heart rate (baseline: 61 ±10, 14-day: 61 ±10 beats·min−1, P = 0.96), minute ventilation (baseline: 8.10 ±1.43, 14-day: 7.82 ±0.98 L·min−1, (P = 0.38), oxygen uptake (baseline: 0.293 ±0.060, 14-day: 0.285 ±0.057 L·min−1, P = 0.43), carbon dioxide production (baseline: 0.245 ±0.051, 14-day: 0.233 ±0.041 L·min−1, P = 0.24) and energy expenditure (baseline: 1.49 ±0.30, 14-day: 1.44 ±0.27 kcal·min−1, P = 0.33). Lower respiratory exchange ratio (baseline: 0.840 ±0.045, 14-day: 0.820 ±0.058, P = 0.03), higher fat oxidation (baseline: 0.078 ±0.031, 14-day: 0.088 ±0.043 g·min−1, P = 0.05), and lower carbohydrate oxidation (baseline: 0.168 ±0.062, 14-day: 0.134 ±0.066 g·min−1, P = 0.03) were observed with 14-day intake of New Zealand blackcurrant extract during supine rest. Twelve participants (75%) had higher fat oxidation during supine rest with for those an increase of 21 ±17%.

**Conclusions:** Whole-body fat oxidation during supine rest was enhanced by 14-day intake of New Zealand blackcurrant extract in males. Enhanced whole-body resting fat oxidation may be due to combined effects of an increase in lipolysis, an increase in blood flow, and increased metabolic handling of fatty acids in the muscle. Our observations on resting substrate oxidation in the present study may indicate that New Zealand blackcurrant extract has application for weight management. However, the dosing strategy to maximize whole-body resting fat oxidation with intake of New Zealand blackcurrant extract is not known.

**Acknowledgments:** Supplementation was provided by Health Currancy Ltd (United Kingdom) and CurraNZ Ltd (New Zealand). Financial support for conference attendance was obtained from Blackcurrant New Zealand Inc (New Zealand).

**Acute Effects of Anthocyanin-rich New Zealand Blackcurrant Extract on Cardiovascular Function During Supine Rest in Healthy Males**

Mark ET Willems^a^, Pelin Bilgiç^b^, Stefano Montanari^a^, Mehmet A Sahin^a,b^

^a^Institute of Sport, Nursing and Allied HealthI, University of Chichester, Chichester, UK; bDepartment of Nutrition and Dietetics, Hacettepe University, Ankara, Turkey

Corresponding author: m.willems@chi.ac.uk

**Background:** Polyphenols in fruits and vegetables provide anti-oxidant, anti-inflammatory and anti-atherosclerotic effects. Reduced risk for cardiovascular disease is likely associated with the effects by polyphenols on blood pressure and arterial stiffness. Studies with 7-day intake of New Zealand blackcurrant extract showed changes during supine rest for cardiovascular parameters. We examined the effects of an acute intake of New Zealand blackcurrant extract on cardiovascular function during supine rest in healthy males.

**Methods:** Healthy physically active males (n = 15, age: 24 ±6 yr, body mass: 78 ±16 kg, height 177 ±7 cm, BMI: 24.7 ±4.3 kg·m−2 (8 normal weight, 6 overweight, 1 obese), body fat: 15 ±5%) volunteered. Participants visited the laboratory for resting measurements at baseline (no supplementation) and 2 hours after intake of two capsules with New Zealand blackcurrant extract (600 mg containing 210 mg of anthocyanins). Capsules were taken one hour after breakfast of one slice of bread and water and 2 hours before testing. After being seated in a chair for 10 min, participants were asked to lie horizontally on a massage table for resting measurements. Whole body cardiovascular measurements were obtained with a beat-to-beat blood pressure monitoring system (Portapres® Model 2, Finapres Medical Systems BV, Enschede, The Netherlands). Expired air was collected for two times for 10 min with Douglas bags and volume measured. Cardiovascular observations during the 10 min with the lowest minute ventilation were analyzed.

**Results:** At supine rest, there was no effect on heart rate, systolic blood pressure, diastolic blood pressure, mean arterial pressure and stroke volume. However, 10 out of 15 participants had lower systolic and diastolic blood pressure values with acute intake of New Zealand blackcurrant extract. There was a trend for cardiac output to be higher by 5% (baseline: 5.68 ±0.71, NZBC: 5.99 ±0.98 L·min−1, P = 0.09, d = 0.36). Total peripheral resistance was reduced by 7% (baseline: 15.67 ±2.85, NZBC: 14.45 ±3.04 mmHg·min·L−1, P <0.05, d = −0.41).

**Conclusions:** In previous work, we observed with 7- and 14-day intake of New Zealand blackcurrant extract larger changes in cardiac output and total peripheral resistance than in the present study. Our observations indicate only a moderate effect on cardiovascular function at rest with acute intake. Future studies need to address whether an acute intake of New Zealand blackcurrant extract is effective in people with hypertension or peripheral arterial disease.

**Acknowledgments:** Supplementation was provided by Health Currancy Ltd (United Kingdom) and CurraNZ Ltd (New Zealand). Financial support for conference attendance was obtained from Blackcurrant New Zealand Inc (New Zealand)

**Effects of Intermittent and Daily Intake of Anthocyanin-rich New Zealand Blackcurrant Extract on Cardiovascular Function During Supine Rest in Healthy Males**

Mark ET Willems^a^, Pelin Bilgiç^b^, Stefano Montanari^a^, Mehmet A Sahin^a,b^

^a^Institute of Sport, Nursing and Allied Health, University of Chichester, Chichester, United Kingdom; ^b^Department of Nutrition and Dietetics, Hacettepe University, Ankara, Turkey

Corresponding author: m.willems@chi.ac.uk

**Background:** Intake of polyphenols results in plasma bioavailability of metabolites that can last for days. Studies have mostly employed dosing protocols that examined observations following acute or daily prolonged intake. We examined the effects of intermittent and daily intake of New Zealand blackcurrant (NZBC) extract over a 14-day period on cardiovascular function during supine rest.

**Methods:** Healthy physically active males (n = 15, age: 24 ±6 yr, body mass: 78 ±16 kg, height 177 ±7 cm, BMI: 24.7 ±4.3 kg·m−2 (8 normal weight, 6 overweight, 1 obese), body fat: 15 ±5%) volunteered. Participants visits included resting measurements at baseline (no supplementation), after 14-day intermittent intake (14-I, i.e. every other day) and 14-day daily intake (14-D) of two NZBC extract capsules (210 mg of anthocyanins for two capsules). Last dose was consumed one hour after breakfast of one slice of bread and water and 2 hours before visiting the laboratory. Cardiovascular measurements were obtained with a beat-to-beat blood pressure monitoring system (Portapres® Model 2, Finapres Medical Systems BV, Enschede, The Netherlands). Expired air was collected for two times for 10 min with Douglas bags and volumes measured. Cardiovascular observations during the 10 min with the lowest minute ventilation were analyzed.

**Results:** During supine rest, there was no effect on heart rate and systolic blood pressure. Lower diastolic blood pressure was recorded and similar for intake conditions [baseline: 70 ±7, 14-I: 64 ±5 (P <0.01, d = −0.99), 14-D: 63 ±9 mmHg (P <0.05, d = −0.87)]. Lower mean arterial pressure was recorded and similar for intake conditions [baseline: 87 ±7, 14-I: 81 ±6 (P <0.01, d = −0.92), 14-D: 81 ±9 mmHg (P = 0.03, d = −0.74)]. Higher stroke volume was recorded only for 14-day daily intake [baseline: 94.9 ±13.4, 14-I: 100.0 ±14.3, 14-D: 103.1 ±18.1 mL (P = 0.01, d = 0.51)]. Cardiac output was higher with a trend for change at 14-day intermittent and a change with 14-day daily intake [baseline: 5.68 ±0.71, 14-I: 6.15 ±0.90 (P = 0.05, d = 0.58), 14-D: 6.14 ±0.88 L·min−1 (P = 0.02, d = 0.58)]. Total peripheral resistance was reduced and similar for intake conditions (baseline: 15.67 ±2.85, 14-I: 13.59 ±2.50 (P <0.01, d = −0.78), 14-D: 13.43 ±2.61 mmHg·min·L−1 (P <0.01, d = −0.82)].

**Conclusions:** Beneficial effects of intake of anthocyanin-rich NZBC extract on resting cardiovascular function can be obtained by intermittent (i.e. every other day) intake of 210 mg of anthocyanins. Future work may want to address the effects of longer intermittent intake than the 2-weeks employed in our study. It would also be of interest to examine plasma bioavailability of anthocyanin-derived metabolites with intermittent intake of NZBC extract.

**Acknowledgments:** Supplementation was provided by Health Currancy Ltd (United Kingdom) and CurraNZ Ltd (New Zealand). Financial support for conference attendance was obtained from Blackcurrant New Zealand Inc (New Zealand).

**Effects of Anthocyanin-rich New Zealand Blackcurrant Extract on Rugby Union Specific Tests**

Paddy Burnett^a^, Mark ET Willems^a^

^a^Institute of Sport, Nursing and Allied Health, University of Chichester, Chichester, UK

Corresponding author: m.willems@chi.ac.uk

**Background:** Rugby union is a contact team sport with athletes requiring multiple performance abilities. New Zealand blackcurrant (NZBC) extract has provided enhanced effects for aerobic and anaerobic exercise tasks for endurance and team sports athletes (doi: 10.1007/s00421-015-3215-8 and doi: 10.1123/ijsnem.2015-0020). The mechanisms for enhanced exercise performance by intake of NZBC extract are still unclear. Previous performance studies on the effects by NZBC extract used mainly a single exercise task. We examined the effects of NZBC extract on the repeated performance in a battery of rugby union specific tests including speed, agility and strength testing.

**Methods:** University males rugby union players (n = 13, age: 21 ±2 years, height: 182 ±6 cm, body mass: 86.9 ±13.3 kg) completed two full familiarizations and two experimental visits in an indoor facility. The study had a double blind, placebo-controlled randomized crossover design. For the experimental visits, participants consumed two capsules a day for seven days of NZBC extract (210 mg/day of anthocyanins) or placebo with a 7-day wash out. Participants were tested for performance in the following order: Running-based anaerobic sprint test, the Illinois agility test, seated medicine ball (3 kg) throw, and hand grip strength. Data were analyzed with two-tailed student t-tests with significance accepted at p ≤0.05 and interpretation of 0.05 >p ≤ 0.1 as a trend.

**Results:** With NZBC extract, there was a strong trend for average sprint time to be higher by 1.7% (placebo: 5.947 ±0.538 s, NZBC extract: 5.846 ±0.571 s, p = 0.06) with 6 participants having changes of more than 3%. In the Illinois agility test, there was also a strong trend for the mean time to be higher by 1.6% (placebo: 18.46 ±1.44 s, NZBC extract: 18.15 ±1.22 s, p = 0.07) with 4 participants having changes of more than 3%. The correlation between the %change in average sprint time and %change in the mean agility time was not significant (pearson R2 = 0.0698, p = 0.383). There were no differences for the seated medicine ball throw (p = 0.106) and hand grip strength (p = 0.709).

**Conclusions:** Intake of anthocyanin-rich NZBC extract in rugby union players seems to improve tasks that require speed and agility but not muscle strength. NZBC blackcurrant extract may be able to enhance exercise performance in team sports that require repeated movements with high intensity and horizontal change of body position without affecting muscle strength.

**Acknowledgments:** Supplementation was provided by Health Currancy Ltd (United Kingdom) and CurraNZ Ltd (New Zealand). Financial support for conference attendance was obtained from Blackcurrant New Zealand Inc (New Zealand).

**Acute Workloads and Chronic Stress Responses to Minimal Equipment Resistance Training with and without Blood Flow Restriction Compared to Traditional Equipment Resistance Training**

Harry P. Cintineo^a^, Alexa J. Chandler^a^, Gianna F. Mastrofini^a^, Blaine S. Lints^a^, Bridget A. McFadden^a^, Shawn M. Arent^a^

^a^Department of Exercise Science, University of South Carolina, Columbia, SC, USA

Corresponding author: sarent@mailbox.sc.edu

**Background:** During periods of limited access to traditional exercise equipment, a minimal equipment resistance training approach can be used to improve or maintain physical performance. Blood flow restriction (BFR) has been proposed as a method of bolstering the effects of this type of training. The internal workloads associated with these types of training and subsequent psychological and physiological responses are unknown. The purpose of this study is to determine stress responses to minimal equipment resistance training with and without BFR compared to traditional resistance training.

**Methods:** ROTC cadets (N = 52; age = 20 ±2 y; 38.5% female) completed a 6-week training intervention after being randomized into one of three groups: traditional equipment (TRAD), minimal equipment (MIN), and minimal equipment with BFR (MIN+BFR). Volume, intensity, and overall workloads were quantified through session duration, rating of perceived exertion (RPE)- and heart rate (HR)-derived workload scores (sRPE and Edward’s TRIMP, respectively), and exercise energy expenditure (EEE). Acute responses to training were quantified through lactate (pre-, mid-, and post-exercise) and daily delayed onset muscle soreness (DOMS) ratings using a 100-mm visual analog scale. Chronic responses (N = 49) were quantified through weekly multicomponent training distress scale (MTDS) scores and pre-to-post basal serum total cortisol concentrations. Linear and generalized linear mixed-effects models with random intercepts for subject ID were used to test for Group effects for session duration, sRPE, TRIMP, EEE, and DOMS as well as Group-by-Time interactions for lactate, MTDS, and cortisol (α = 0.05).

**Results:** No group differences were found for session duration (P = 0.077). Group main effects were found for sRPE (P = 0.002), TRIMP (P <0.001), and EEE (P = 0.003). Post-hoc tests revealed lower sRPE and TRIMP in TRAD compared to MIN and MIN+BFR (P <0.02), with no differences between MIN and MIN+BFR (P >0.90). A Group-by-Time interaction was found for lactate (P <0.001). Post-hoc tests showed lower lactate at mid- and post-exercise in TRAD compared to MIN and MIN+BFR (P <0.001), with no differences between MIN and MIN+BFR (P >0.57). No group differences were found for DOMS (P = 0.52). No Group-by-Time interactions or Group main effects were found for MTDS (P >0.29) or cortisol (P >0.21).

**Conclusions:** Despite similar durations, traditional equipment training results in lower sRPE, TRIMP, EEE, and lactate responses. However, all groups experienced similar DOMS and chronic psychological and physiological responses to training. These findings suggest that although internal workloads are lower in TRAD, DOMS and the subsequent psychological and physiological responses are similar between traditional equipment compared to minimal equipment training with and without BFR.

**Acknowledgments:** Funding provided by the United States Department of Defense.

**Trial Registration:** ClinicalTrials.gov (NCT05003778)

**Consistency is Key: Body Composition and Exercise Energy Expenditure Throughout a National Championship Season**

Alexa J. Chandler^a^, Harry P. Cintineo^a^, Bridget A. McFadden^a^, Molly E. Binetti^b^, Gianna F. Mastrofini^a^, Blaine S. Lints^a^, Shawn M. Arent (FISSN)^a^

^a^Department of Exercise Science, Arnold School of Public Health, University of South Carolina, Columbia, SC, USA^b^Gamecock Sports Science, Department of Athletics, University of South Carolina, Columbia, SC, USA

Corresponding author: sarent@mailbox.sc.edu

**Background:** Basketball is a sport consisting of repeated high-intensity bouts, and previous research has shown athletes spend the majority of game time at >85% heart rate maximum. When these high workloads aren’t matched with adequate energy or macronutrient intakes, downturns in body composition can occur. As such, practitioners need to understand the energy demands of different sports. Exercise energy expenditure (EEE), determined through wearable technologies, is a useful metric that can be used to develop nutritional recovery strategies aimed to maintain muscle mass. The purpose of this observational study was to assess body composition in female college basketball players throughout a competitive season that ended in a national championship and to quantify EEE during different parts of the season.

**Methods:** Division I female basketball players (N = 16; age = 20 ± 1 y) were observed over the 2021-2022 season. Body composition was assessed via air displacement plethysmography prior to preseason and immediately following the final game of the postseason. Body composition variables assessed were body mass (BM), body fat percentage (BF%), fat mass (FM), and FFM (kg). EEE was monitored during all practices and games using a team-based heart rate monitoring system (Polar Team Pro). Each body composition variable was analyzed using a paired-samples t-test with an alpha level of 0.05. Session EEE was averaged for different periods of the season (preseason, in-season, post-season), and descriptive statistics are displayed as mean ± standard deviation.

**Results:** Paired-samples T-tests revealed no significant changes in BM, BF%, FM, or FFM from pre- to postseason (P >0.05). Average session EEE during different blocks of the season included: preseason = 1116 ± 329 kcal, in-season = 1020 ± 405 kcal, postseason = 1058 ± 489 kcal.

**Conclusions:** The primary finding of this analysis is a lack of change in all body composition variables coupled with high, yet stable, session EEE across different parts of the season in this highly competitive Division I team. This shows that the athletes were able to match the workloads with adequate energy intake. Further, though not assessed, proper strength and conditioning training and macronutrient, especially protein, intake during the season likely contributed to maintenance of FFM. Overall, this team demonstrated proper athlete management and recovery strategies, and this study contributes to the understanding of EEE during practices and games. These findings can help coaches and practitioners determine nutritional strategies to ensure FFM maintenance and optimal performance.

**Road to the NCAA Championship: Internal and External Load Metrics in Women’s Division I Basketball Athletes**

Bridget A. McFadden^a^, Harry P. Cintineo^a^, Alexa J. Chandler^a^, Gianna F. Mastrofini^a^, Blaine Lints^a^, Molly Binetti^a^, Shawn M. Arent^a^

^a^University of South Carolina, Columbia, SC, USA

Corresponding author: BM39@mailbox.sc.edu

**Background:** National Collegiate Athletic Association (NCAA) women’s basketball consists of a demanding ~6-month season with the best teams advancing to regional and national conference tournament play. Workload changes from regular-season to tournament games are largely unknown, along with differences between starters and nonstarters. The purpose of this observational study was to assess differences in internal and external workload between regular-season and conference tournament games over the course of a women’s basketball national championship season.

**Methods:** Fifteen Division I women’s basketball players were monitored throughout the competitive season. Players wore an accelerometer and heart rate monitor (Polar Team Pro) during all games (N = 36) to determine training load (TL), exercise energy expenditure (EEE), distance covered (DIS), and time spent in HR zones 4 (HRZ4 = 80-80%HR max) and 5 (HRZ5 = 90-100% HR max) combined (HRZ4+5). A 2 × 2 mixed effects model was used to assess workload differences between regular-season (n = 27) and tournament (n = 9) games as well as ‘starting status’ with 4 starters (S) and 11 nonstarters (NS). One S was not included in analysis due to a lost HR monitor during tournament play. Significance was set at P <0.05.

**Results:** A Time-by-Starting Status interaction was found for HRZ4+5 (P = 0.006). Post-hoc tests showed NS was lower than S during both regular-season (P <0.001) and tournament (P <0.001), and NS decreased from regular-season to tournament (P = 0.034) while S maintained (P = 0.062). No other interactions were observed, but main effects for Starting Status were found for all workload variables (P <0.001) indicating higher values in S compared to NS for all metrics.

**Conclusions:** S showed the greatest workloads compared to NS during regular-season and tournament games. TL, DIS, and EEE were consistent for both S and NS from regular season to tournament games. However, there was a trend for increased time spent in HRZ4+5 for S, coupled with significant declines for NS during tournament play. This may be a result of an increased intensity of tournament play. It may also be a result of substitutions strategies which limit the playing time of S during the regular season in an effort to optimize athlete readiness for tournament play. During tournaments, the ‘win or go home’ nature of play may preclude the necessity of these substitutions in favor of keeping the best players on the court. Team success in a national championship run may depend on the ability to maintain workload consistency coupled with proper recovery throughout the entirety of the season.

**Acknowledgments:** Thank you to the University of South Carolina Women’s Basketball National Champions

**Inflammatory, Muscle Damage, and Immune Changes After Supplementation with Inactivated and Activated *Bacillus coagulans* GBI-30, 6086**

Kevin Holley^a^, Petey W. Mumford^a^, Athena Viers^a^, Jessica M. Moon^b^, Julia C. Blumkaitis^a^, Anthony Hagele^a^, Kayla M. Ratliff^a^, Johnathan Boring^a^, Connor Gaige^a^, Logan Orr^a^, Kylie Walden^a^, Richard A. Stecker^a^, Kyle L. Sunderland^a^, Ralf Jäger FISSN^c^, Chad M. Kerksick FISSN^a^

^a^Exercise and Performance Nutrition Laboratory, College of Science, Technology, and Health, Lindenwood University, St. Charles, MO, USA; ^b^School of Kinesiology, University of Central Florida, Orlando, FL, USA; cIncrenovo LLC, Milwaukee, WI, USA

Corresponding author: ckerksick@lindenwood.edu

**Background:** Exercise has been shown to elicit acute physiological responses within the body that can cause transitory alterations within the immune system ranging from increased proinflammatory cytokines to reductions in various immune cell activity. Attempts to supplement or modify the diet have been proposed to aid the immune system’s response to these alterations. The purpose of this study was to identify the impact of supplementing with inactive and active cultures of *Bacillus coagulans* GBI-30, 6086 on immune markers following a muscle damaging high-volume dose of resistance exercise.

**Methods:** 76 healthy, resistance trained men (29 ± 9 years, 178.8 ± 6.9 cm, 87.6 ± 11.8 kg) were randomly assigned in a double-blind, parallel-group design to supplement for 14 days with 1 billion CFU/day of either an inactivated culture of B. Coagulans GBI-30, 6086 (INBC30, Kerry), an active culture of B. coagulans GBI-30, 6086 (BC30, Kerry), or a maltodextrin placebo (PLA). Participants completed a muscle damaging high-volume dose of resistance exercise, where blood was collected pre-exercise and 0-, 0.5-, 1-, 2-, 5-, 24-, 48-, and 72-hr post-exercise to assess markers of inflammation, muscle damage, and the immune response.

**Results:** A significant interaction occurred (p = 0.02) for changes in IL-6 between PLA and INBC30 where PLA had higher IL-6 concentrations than INBC30 at 0.5-hr and 24-hr post exercise. However, at 2-hr, PLA had lower IL-6 concentrations compared to INBC30. A significant interaction occurred (p = 0.05) for changes in IL-6 between PLA and BC30. PLA had significantly greater increases in IL-6 concentration at 5-hr (p < 0.001) and 72-hr (p < 0.01) after exercise. A significant group x time interaction occurred (p = 0.005) for changes in IL-10 between PLA and INBC30 whereby IL-10 values in PLA were higher than INBC30 at 24-hr post-exercise (p < 0.01). A significant interaction occurred (p = 0.001) for changes in IL-10 between PLA and BC30 where PLA was higher than BC30 at 2-, 5-, 24-, and 48-hr following exercise. No differences were observed for all other inflammation, immune, or muscle damage markers.

**Conclusions:** Supplementation with INBC30 or BC30 lowers specific inflammatory markers that may help participants reduce systemic inflammation following a muscle damaging high-volume, muscle-damaging dose of resistance exercise in healthy resistance trained men.

**Acknowledgments:** This research was funded by an unrestricted grant from Kerry.

***Bacillus coagulans* GBI-30, 6086 improves amino acid absorption from plant protein in older women**

Kristen N. Gross^a^, Kylie E. Walden^a^, Anthony M. Hagele^a^, Logan S. Orr^a^, Joesi M. Krieger^a^, Martin Purpura^b^, Ralf Jäger, FISSN^b^, Chad M. Kerksick, FISSN^a^

^a^Exercise and Performance Nutrition Laboratory, School of Health Sciences, Lindenwood University, St. Charles, MO, USA; ^b^Increnovo LLC, Milwaukee, WI, USA

Corresponding author: ckerksick@lindenwood.edu

**Background:** *Bacillus coagulans* GBI-30, 6086 (BC30, Kerry) has previously been shown to increase protein digestion in an in vitro model of the stomach and small intestine and amino acid appearance in healthy men and women after ingestion of milk protein concentrate. The impact of ingesting BC30 with other protein sources, and BC30 alone in other demographics, is largely unknown. The purpose of this study was to examine the impact of adding BC30 to a 20-gram dose of a blend of rice and pea protein on post-prandial changes in blood amino acids concentrations in healthy, older women.

**Methods:** Healthy, older females (n = 30, 58.5 ±5.2 years, 165.4 ±6.8 cm, 65.6 ±8.8 kg, 23.7 ±3.2 kg/m2) completed two separate 14-day supplementation protocols separated by a 3-week washout period. Participants were instructed to ingest a 20-gram daily dose of a blend of rice and pea protein (ProDiem Complete PR, Kerry) with (PPCBC30) or without (PPC) the addition of 1 × 109 CFU *Bacillus coagulans* GBI-30, 6086. Body composition and demographics were assessed upon arrival to the laboratory. Upon ingestion of their final assigned supplemental dose, blood samples were taken at 0 (baseline), 30-, 60-, 90-, 120-, 180-, and 240-minutes post-consumption and analyzed for amino acid concentrations.

**Results:** Alanine (p = 0.02), tryptophan (p = 0.003), cysteine (p = 0.04), essential amino acids (p = 0.05), and total amino acids (p = 0.04) all exhibited significantly greater AUC with PPCBC30 when compared to PPC. In addition, tryptophan (p = 0.003), cysteine (p = 0.02), essential amino acids (p = 0.05) and total amino acids (p = 0.04) displayed significantly greater concentration maximum (CMax) values in PPCBC30 when compared to PPC. Finally, time to reach CMax (TMax) was similar between conditions with 80% of all measured amino acids and amino acid combinations achieving CMax at a similar time (~60-minutes). Following qualitative (non-inferential) assessment, 88% of all measured outcomes achieved a higher AUC with PPCBC30 and 100% of all outcomes achieved a higher CMax with PPCBC30.

**Conclusions:** In concert with previous findings in a younger mixed gender cohort with milk protein, the addition of BC30 to a daily 20-gram dose of plant protein concentrate in healthy older women improved AUC and CMax values in several individual amino acids and amino acid combinations. Follow-up research should further examine the impact of aging on amino acid absorption in addition to whether BC30 and protein co-ingestion can improve other health-related outcomes.

**Acknowledgments:** This research was funded by Kerry

**Health Status, but not Commercial Coffee Intake Differentially Affects Glycation Markers in Middle-Aged Men and Women**

J. Knicely, J. Sturgill, K. Scanlon

University of Mount Union, Alliance, OH 44601

**Background:** The purpose of this observational study was to investigate the presence of a diagnosed metabolic condition and the relationship between habitual coffee consumption on glycated hemoglobin (HbA1c) and advanced glycation end products (AGE) with a concentration on sex-disaggregated data analysis. An estimated 17.5% of adults between 45-64y living in the United States have type 2 diabetes mellitus and another 41.7% have prediabetes with a greater probability of those being male (2018 US Census Bureau Data). Previous research suggests women with type 2 diabetes that consume a minimum of four cups of black coffee per day, either caffeinated or non-caffeinated varieties, were at lower risk for developing cardiovascular diseases and all-cause mortality (Zhang, et al 2019). In prediabetic individuals, Lee et al. observed the progression of type 2 diabetes was slowest in individuals who drank black coffee at least three times per day. Preliminary data suggest that coffee-habituated college students exhibit an inverse relationship between cups per day and HbA1c regardless of physical activity. Chlorogenic acid found in coffee may affect this interaction leading to altered sugar metabolism in those coffee habituated versus coffee naïve (Lowery, 2020). We hypothesized that free-living participants 45-64y would exhibit an inverse relationship between self-reported coffee intake and HbA1c and AGE scores, with an overall higher AGE and HbA1c exhibited in males when compared to female participants.

**Materials and Methods:** To measure HbA1c, mixed capillary blood (5 ul) was obtained and analyzed via a PT Diagnostics HbA1c analyzer (Indianapolis, IN). Participants (N = 32) extended their forearm for ultraviolet light emittance and tissue fluorescence via AGE reader (AGE Reader, Diagnostics, Inc. Groningen, Netherlands). Lastly, using visual aids, participants estimated how many ounces of coffee they regularly consume per day and whether they had a diagnosed metabolic condition.

**Results:** A 2 × 2 factorial ANOVA indicated a significant difference in glycated hemoglobin (p = 0.0002), but not AGE (p = 0.07) in the presence of a diagnosed metabolic condition between males and females with more males reporting a diagnosed metabolic disease. No significant difference was indicated between sex and glycated hemoglobin or AGE by coffee consumption (p &gt; 0.05).

**Conclusion:** Those diagnosed with a metabolic condition were more likely to have higher HbA1c percentages and increased AGE. However, sex nor coffee consumption was a driving factor in these results based on our sample population. Future investigators should seek equal representation of sex and routine quantity of daily coffee consumption when exploring these questions.

**The acute Effects of Adenosine 5_’-Triphosphate Disodium (PeakATP) Supplementation Vs. Placebo on Measures of Reaction Time Following All-Out High-intensity Exercise**

Jessica M Moon^a^, Trevor J. Dufner^a^, Adam J Wells^a^

^a^University of Central Florida, Orlando, FL, USA

Corresponding author: jessica.moon@ucf.edu

**Background:** Adenosine triphosphate (ATP) supplementation has previously demonstrated beneficial effects including improved strength, power, and body composition, reduced fatigue, and enhanced recovery. However, the effect of ATP supplementation on measures of cognition such as processing speed has not been investigated. The purpose of this study was to examine the effects of two-weeks ATP supplementation (PeakATP) versus placebo (PLA) on visuomotor reaction time (RT) following all-out high-intensity exercise.

**Methods:** Twenty recreationally active adults (22.3 ±4.4 yrs, 169.9 ±9.5 cm,78.7 ±14.6 kg,27.0 ±9.5%fat) were randomly assigned to 14-days supplementation with either 400 mg PeakATP or PLA in a double-blind, counter balanced crossover design. Participants completed two experimental trials separated by a 14-day washout period. During each trial participants ingested an acute dose of their assigned supplement 30-minutes before completing pre-exercise (PRE) RT assessments consisting of Mode A (proactive) and Mode B (reactive) visuomotor RT tests performed on the Dynavision D2. The number of hits (hits) and average RT per hit (avgRT) in Mode A and B, and the number of misses in Mode B were assessed. Participants then completed a standardized warm-up followed by a three-minute all-out effort on a cycle ergometer. RT tests were repeated immediately-post (IP) and 60-min post-exercise (60P).

**Results:** Significant time x treatment interactions were observed for number of hits and avgRT in Mode A (p_’s_ = .006). In both cases, significant time effects were noted for PLA (p = .002 and p = .004, respectively), but not PeakATP (p = .187 and p = .211, respectively). In PLA, avgRT was significantly slower and the number of hits significantly lower at IP (avgRT p = .027; Hits p = .019) and 60P (avgRT p = .002; Hits p <.001) compared to PRE. avgRT was significantly faster (p = 0.015), and the number of hits was significantly greater (p = 0.28) in PeakATP at 60P compared to PLA. A significant time x treatment interaction was also observed for avgRT in Mode B (p = .039). A significant time effect was noted for Peak ATP (p = .002), but not PLA (p = .925). In PeakATP, avgRT was significantly faster at IP (p = .015) and 60P (p = .001) compared to PRE but was not significantly different than PLA at any time point. No significant interaction or main effects were noted for number of hits in Mode B (p_’s_>.05). A significant treatment effect was noted for number of misses (p = .005), with misses being significantly lower in PeakATP overall compared to PLA.

**Conclusions:** PeakATP supplementation attenuated the decline in proactive visuomotor RT, enhanced reactive visuomotor RT and reduced the number of misses during the reactive visuomotor task.

**Acknowledgments:** This study was funded by TSI Group Limited.

**The Effects of Increasing Dietary Protein Intakes on Body Fat in Non-resistance Trained Females**

Kara Phillips^a^, Gianna Mastrofini^a^, Jacob Broeckel^a^, Alex Brooks^a^, Alexis Belcher^a^, Rashed Daher^a^, Malena Sellen^a^, Benjamin Berluti^a^, Brooke Morrisseau^a^, Karina Noboa^a^, Alexa Rukstela^a^, Emily Pribula^a^, Bill I. Campbell^a^

^a^Performance and Physique Enhancement Laboratory, University of South Florida, Tampa, Florida, USA

Corresponding author: bcampbell@usf.edu

**Background:** Previous research has shown that high protein diets (>1.6 g protein/kg bodyweight) decrease fat mass when combined with resistance training in trained populations. One purpose of this study was to determine if these findings hold true in an untrained female population beginning a training program.

**Methods:** Forty untrained women participated in this study. Subjects were matched according to pre-study protein intakes and then randomly assigned to a high protein (HP; n = 23; age 19.9 ±1.1 years) or control group (CON; n = 17; age 19.7 ±1.2 years). Following baseline measurements, subjects were randomly sorted into groups based on baseline responses to a Protein Food Frequency Questionnaire (PFFQ). The HP group was instructed to track all calories and consume a minimum of 2.2 g protein/kg bodyweight per day. The CON group was instructed to maintain normal dietary habits. During the study, both groups had unlimited access to personal nutrition coaches to ensure maximum adherence. Both groups participated in the same supervised resistance-training program for 8 weeks. Body composition was assessed at baseline (within one week prior to workouts commencing) and post-study (within one week of workout cessation) via InBody® 570 Body Composition Analyzer (Biospace, Inc. Seoul, Korea). Data were analyzed via a 2 × 2 repeated measures ANOVA.

**Results:** There were no baseline differences between the groups for protein intake (assessed via PFFQ), bodyweight, fat mass, or body fat percentage. At post-study, there was a significant difference between group PFFQ scores, with the HP group having a greater score indicative of a higher protein intake (p <0.001). While there was a significant time effect for decrease in body fat percentage (p = 0.004), there were no differences between groups for body weight (HP: ▲+1.1 kgs; 60.7 ±8.6 to 61.8.6 ±8.3 kgs; CON: ▲+0.6 kgs; 59 ±9.7 to 59.6 ±9.5 kgs; p = 0.319), body fat mass (HP: ▲-0.4 kgs; 19 ±6.2 to 18.6 ±6.1 kgs; CON: ▲-0.1 kgs; 17.6 ±7.0 to 17.5 ±6.8 kgs; p = 0.627), or body fat percentage (HP: ▲-1.1%; 30.6 ±6.7 to 29.5 ±6.7 %; CON: ▲-0.5%; 29 ±7 to 28.5 ±6.9 %; p = 0.269).

**Conclusions:** While increased protein intake has been shown to decrease fat mass when combined with resistance training in trained populations, this does not appear to be the case for an untrained population. In untrained individuals, the novelty of the resistance training stimulus is likely large enough to obscure any additional benefits an increased protein intake could confer. Future research should aim to investigate at what point in the training lifespan an increased protein intake begins to advantage the trainee.

**The Effects of Macronutrient Tracking Prioritizing Protein Intake on Hunger and Eating Behaviors in Untrained Females**

Gianna Mastrofini^a^, Kara Phillips^a^, Traci Smith^a^, Sebastian Ehmann^a^, Joshua Rogers^a^, Yasamian Alsayed^a^, Katie Haff^a^, Denise Shoucair^a^, Zachary Warhul^a^, Samantha Skinnider^a^, Philip Carvalho^a^, Bill I. Campbell^a^

^a^Performance and Physique Enhancement Laboratory, University of South Florida, Tampa, Florida, USA

Corresponding author: bcampbell@usf.edu

**Background:** Previous research has reported high protein diets increases fat-free mass when combined with resistance training. A common method to verify increased protein intake is via the use of a macronutrient tracking application. However, conflicting research suggests the use of macronutrient trackers are associated with eating disorder symptomology. One purpose of this study was to increase protein intake, document the increase with a macronutrient tracking application, and evaluate if there were any effects on the eating behaviors of previously untrained females initiating a resistance training program.

**Methods:** Forty untrained women participated in this 8-week study. They were matched according to pre-study protein intake (via responses to a Protein Food Frequency Questionnaire [PFFQ] and then randomly assigned to a tracking; [TRACK; n = 23; age 19.9 ±1.1 years] or control group [CON; n = 17; age 19.7 ±1.2 years]). Participants in the TRACK group were instructed to track daily macronutrient intakes with the requirement to consume 2.2 g protein/kg bodyweight/day. The CON group were instructed to not change their dietary habits. During the final seven days of the intervention, CON participants completed a food recall form where they recorded all food and drink that were reflective of their macronutrient intakes during the previous seven weeks of the intervention. Participants engaged in supervised resistance training three times/week. Baseline and post-intervention hunger, restraint, and disinhibition were measured with the 51-item Three-Factor Eating Questionnaire (TFEQ). Additionally, at the same timepoints a three-item Likert scale was used to measure the prior week’s feelings of fullness, desire to eat, and ease of following their diet. Data were analyzed via a 2 × 2 repeated measures ANOVA.

**Results:** Protein intakes were significantly higher in TRACK as compared to CON (p <0.001). There were no significant differences between the two groups for TFEQ measures of hunger (p = 0.327) or disinhibition (p = 0.899). Dietary restraint values favored higher protein intake (TRACK: 8.8 ± 4.6 to 9.6 ± 4.6; CON: 8.2 ± 4.4 to 7.6 ± 4), and while these values trended in opposing directions, the results did not reach the level of statistical significance (p = 0.057). Likewise, there were no significant differences between the groups for the prior week’s feelings of fullness (p = 0.258), desire to eat (p = 0.391), and ease of following their diet (p = 0.465).

**Conclusions:** Increasing protein intake and documenting the increase via a macronutrient tracking application did not appear to have any deleterious effects on psychometric data as compared to a non-tracking control group in previously untrained females.

**Comparison of Macronutrient-based Tracking vs. Intuitive Eating for Increasing Protein Intakes in Females Initiating a Resistance Training Program**

Jacob Broeckel^a^, Alex Brooks^a^, Kara Phillips^a^, Gianna Mastrofini^a^, James Gegenheimer^a^, Arielle Parks^a^, Kworweinski Lafontant^a^, Sandra Korte^a^, Adrianna Gonzalez^a^, Savannah Ericksen^a^, Samantha Gutierrez^a^, Sydney Monahan^a^, Ainne Cortes^a^, Bill I. Campbell^a^

^a^Performance and Physique Enhancement Laboratory, University of South Florida, Tampa, Florida, 33,620, USA

Corresponding author: bcampbell@usf.edu

**Background:** Increasing protein intakes has been shown to improve training adaptions to resistance exercise. Typical protein intake recommendations to maximize resistance training adaptations range from 1.6-2.2 g/kg bodyweight. Methods for increasing protein intakes vary by individual, with some preferring an intuitive approach in which they consciously consume higher protein foods without quantifying meal-by-meal and total daily protein intakes. Others prefer a quantitative approach and embrace macronutrient tracking such that all protein intakes are tracked in order to reach a specific daily protein intake goal. A purpose of this investigation was to determine if an intuitive approach is as effective as macro tracking for increasing total daily protein intakes in untrained females.

**Methods:** Forty-six untrained females participated in the study. Participants were matched according to baseline protein intake (via a Protein Food Frequency Questionnaire [PFFQ]) and then randomized to either a Macro Tracking (TR; n = 23; age: 19.9 ±1.1 years; weight 60.7 ±8.6 kgs) or Non-Tracking (NT; n = 23; age: 20.1 ±1.1 years; weight 64.2 ±8.9 kgs) group for an 8-week period in which all subjects participated in a supervised, resistance training program. TR were instructed to track all macronutrients via a mobile app (MyFitnessPal, San Francisco, CA). TR was given a goal to reach a daily protein intake of 2.2 g/kg/bodyweight, without manipulation of carbohydrates or fats. NT were instructed to avoid any form of food/macronutrient tracking during the study. Rather, NT were instructed to double daily protein intake intuitively via increasing servings of protein containing foods. During the final 7-days of the intervention, NT participants completed a food recall form in which they recorded all foods and drinks that was reflective of their macronutrient intakes during the previous 7-weeks of the intervention. Changes in total daily protein intake (via PFFQ) for each group was analyzed via a paired samples t-test. Between group comparisons for baseline protein intake data (via PFFQ) and end of study daily protein intakes were analyzed via an independent samples t-test.

**Results:** Daily protein intakes increased in both TR (p <0.001) and NT (p = 0.016) during the 8-week study. Absolute (TR = 125 g/day; NT = 90 g/day) and relative protein intake (TR = 2.0 g/kg; NT = 1.4 g/kg) was significantly greater in TR (p <0.001). No differences between groups were observed for the other macronutrient intakes.

**Conclusions:** Setting a daily protein intake goal and subsequently tracking daily protein intake is superior to intuitively increase protein intakes in terms of ingesting optimal amounts of daily protein intakes to maximize resistance training adaptations.

**Effects of a Ready-to-drink Thermogenic Beverage on Resting Energy Expenditure and Hemodynamic Variables**

Christian Rodriguez^a^, Matthew T. Stratton^a^, Madelin R. Siedler^a^, Patrick S. Harty^a^, Jake R. Boykin^a^, Jacob J. Green^a^, Dale S. Keith^a^, Sarah J. White^a^, Brielle Dehaven^a^, Ethan Tinoco^a^, Alexandra Brojanac^a^, Lem W. Taylor^b^, Grant M. Tinsley^a^

^a^Department of Kinesiology & Sport Management, Energy Balance and Body Composition Laboratory, Texas Tech University, Lubbock, TX, USA; ^b^Human Performance Laboratory, School of Exercise and Sport Science, University of Mary Hardin-Baylor, Belton, TX, USA

Corresponding author: grant.tinsley@ttu.edu

**Background:** Thermogenic supplements are often consumed by individuals seeking to improve energy levels and reduce body fat. These supplements are sold in powdered or ready-to-drink (RTD) forms and consist of a blend of ingredients such as caffeine, green tea extract, and other herbal compounds. While there is evidence that thermogenic supplements can positively affect resting energy expenditure (REE), the effect varies based on the combination of active ingredients. Additionally, there is some concern that thermogenic supplements may cause unwanted side effects on hemodynamic variables, like heart rate (HR) and blood pressure (BP). Therefore, further investigation into the efficacy and safety of commercially available products is warranted

**Methods:** Twenty-eight individuals (14 F, 14 M; age: 23.3 ±3.9 yrs; height: 169.4 ±8.6 cm; body mass: 73.3 ±13.1 kg) completed two visits in a randomized, double-blind, crossover fashion. Each visit began with baseline REE, HR, and BP assessments, which were followed by ingestion of an active RTD thermogenic beverage (RTD; OxyShred Ultra Energy) or placebo (P). Assessments were repeated at ~35-50 and ~85-100 minutes post-ingestion. Repeated-measures analysis of variance was performed with condition and time specified as within-subjects factors. Follow up for significant effects was performed using pairwise comparisons with Tukey adjustment, and statistical significance was accepted at p <0.05.

**Results:** A significant condition X time interaction was observed for REE (p <0.0001). Pairwise comparisons indicated no difference in baseline REE (p = 0.99; difference [mean±SE]: −0.6 ±1.3%) but higher REE values at 35-50 min (p = 0.005; difference: 5.1 ±1.3%) and 85-100 min (p = 0.007; difference: 5.3 ±1.3%) after RTD ingestion as compared to P. No significant condition X time interactions were observed for respiratory quotient, HR, or BP (p = 0.12 to p = 0.32). Condition main effects indicated lower HR with RTD than PL (−3.0 ±0.9 bpm; p = 0.002), but higher BP (systolic: 3.5 ±1.1 mmHg; p = 0.003; diastolic: 3.5 ±0.9 mmHg; p = 0.0006), although values remained within normal ranges for all hemodynamic variables.

**Conclusions:** The results of this analysis suggest that acute ingestion of a novel thermogenic RTD beverage significantly increases REE, and this elevated caloric expenditure is sustained for at least 100 minutes following ingestion. While minor differences in hemodynamic variables were observed between conditions, no effect of the RTD beverage was confirmed due to lack of statistical interactions. As such, individuals aiming to increase energy expenditure may benefit from acute ingestion of a RTD thermogenic supplement.

**Acknowledgments:** This study was funded by EHPLabs. The authors declare no conflicts of interest.

**Trial Registration:** ClinicalTrials.gov Identifier: NCT05194475

**Beneficial Impact of a Thermogenic Energy Drink on Measures of Perceived Energy, Focus, Concentration, Alertness, and Mood**

Patrick S. Harty^a^, Christian Rodriguez^a^, Matthew T. Stratton^a^, Madelin R. Siedler^a^, Jake R. Boykin^a^, Dale S. Keith^a^, Jacob J. Green^a^, Brielle Dehaven^a^, Ethan Tinoco^a^, Sarah J. White^a^, Alexandra Brojanac^a^, Lem W. Taylor^b^, Grant M. Tinsley^a^

^a^Department of Kinesiology & Sport Management, Energy Balance & Body Composition Laboratory, Texas Tech University, Lubbock, TX, USA; ^b^Human Performance Laboratory, School of Exercise & Sport Science, University of Mary Hardin-Baylor, Belton, TX, USA

Corresponding author: grant.tinsley@ttu.edu

**Background:** Thermogenic energy drinks are commonly consumed by active individuals seeking to increase energy levels and accelerate fat loss. However, less is known regarding the influence of these products on subjective outcomes. Thus, the purpose of this investigation was to examine the effects of a ready-to drink thermogenic beverage (RTD) on measures of self-reported energy, focus, concentration, alertness, and mood.

**Methods:** Healthy males and females participated in this study (n = 28; 23.3 ±3.9 years; 169.3 ±8.6 cm; 73.3 ±13.1 kg; average caffeine consumption: 302 ±118 mg·day−1). After 50 minutes of supine rest, participants were provided with RTD (OxyShred Ultra Energy, EHPlabs) or placebo in a randomized, double-blind, placebo-controlled, crossover fashion. Measures of energy, focus, concentration, alertness, and mood were collected via visual analog scale throughout each visit: upon arrival (Pre1), 50 minutes following arrival (Pre2), immediately following beverage consumption (Post1), 50 minutes following consumption (Post2), and 100 minutes following consumption (Post3). Repeated-measures analysis of variance tests were performed with condition and time specified as within-subjects factors. Follow up tests were performed using pairwise comparisons with Benjamini & Hochberg adjustments. Statistical significance was accepted at p <0.05.

**Results:** Significant condition X time interactions were present for all VAS variables (p <0.0001 to p = 0.02). Ratings of energy, focus, concentration, and alertness were higher in RTD than placebo (PL) at the Post2 and Post3 time points (p = 0.007 to p = 0.03). Mood ratings were higher in RTD than PL at Post3 (p = 0.047). In RTD, energy was lower at Pre2 and higher at Post1, Post2, and Post3 compared to Pre1 (p = 0.002 to p = 0.02). Focus, concentration, and alertness in the RTD condition were lower at Pre2 and higher at Post2 compared to Pre1 (p = 0.003 to p = 0.04). In RTD, mood ratings were higher at Post3 compared to Pre1 (p = 0.047). In PL, energy, focus, concentration, and mood did not increase from Pre1 at any subsequent timepoints; alertness was lower at Pre2 and higher only at the Post1 time point compared to Pre1 (p = 0.003 to p = 0.03).

**Conclusions:** A thermogenic RTD product positively influences subjective energy, focus, concentration, alertness, and mood in active individuals. Additional long-term studies are needed to examine whether these changes could result in increased adherence to exercise regimens and greater enjoyment of training, potentially leading to improved fitness outcomes with regular consumption.

**Acknowledgments:** This study was funded by EHPLabs. The authors declare no conflicts of interest.

**Trial Registration:** ClinicalTrials.gov Identifier: NCT05194475

**Dead Bodybuilders Speaking from the Heart: A Case Series Analysis of Autopsy Reports of Bodybuilders who Died Prematurely Between 2010 and 2022**

 

Dillon Darrow^a^, Rick Collins^b^, Daniel Gwartney^c^, Guillermo Escalante^a^

^a^Department of Kinesiology, California State University San Bernardino, San Bernardino, CA, USA; ^b^Collins Gann McCloskey & Barry PLLC, Mineola, NY, USA; ^c^Daniel Gwartney, Columbia, MO, USA

Corresponding author: gescalan@csusb.edu

**Background:** This study analyzed publicly available autopsy reports of male bodybuilders under the age of 50 who reportedly died from cardiovascular related events.

**Methods:** A general Google search with the terms ‘dead bodybuilders’ was performed on 2/10/22 yielding results to 18 websites in the first 2 pages. Each source was visited individually to identify male bodybuilders who died under age 50 from any cause over the last 12 years. Results were further filtered by those who reportedly died due to a heart attack, heart failure, other cardiovascular events (e.g. stroke, embolism, etc.), natural causes, or unknown causes where the place/date of death were available. Bodybuilders who died outside of the United States were excluded from the analysis as autopsy reports were unobtainable. In total, 14 bodybuilders were identified, and members of our research team contacted individual county coroner’s offices to request full autopsies.

**Results:** A total of 6 reports were available for review and analysis. They had the following means (± SD): Age = 36 ± 7.1 years; height 1.82 ± 0.02 m; weight = 103.8 ± 5.3 kg; BMI = 31.6 ± 2.3 m/kg2; weight of heart = 575 ± 134.4 g; and left ventricular thickness (n = 3) = 16.3 ± 3.5 mm. Prevalence of left ventricular hypertrophy was 100%, chamber dilation was 33%, atherosclerosis (n = 5) was 80%, positive toxicology report for illicit drugs (n = 5) was 60%, positive for anabolic steroids was 67%, and any drugs found in their possession was 100%. Causes of death reported by coroners included heart disease, steroid-induced cardiomyopathy, sudden cardiac dysrhythmia, and left ventricular hypertrophy.

**Conclusions:** The bodybuilders analyzed had a mean heart weight that is 73.7% heavier than the reference mean (575 g vs 332 g). Similarly, 100% of the autopsies reported left ventricular hypertrophy with a mean thickness of 16.3 ± 3.5 mm; this is 125% thicker than normative data for men. While abuse of AAS for prolonged periods of time may contribute to some of the cardiac abnormalities present in these bodybuilders, it should be noted that cardiac hypertrophy, including left ventricular hypertrophy, has also been reported in drug-free strength athletes. Each autopsy report included cardiovascular abnormalities within the cause of death. Association does not mean causation, but nonetheless bodybuilders should be aware of potential contributing risks with AAS abuse.

**Acknowledgments:** Appreciation to Cristina Amador for research assistance.

**Comparisons of the Apple Watch and a Metabolic Cart during VO2 Max Testing in Physically Active Adults**

Flavia Rusterholz^a^, Corey A. Peacock^a^, Andrew Rodriquez^a^, Victoria Ortiz^a^

^a^Department of Health and Human Performance, Nova Southeastern University, Ft. Lauderdale FL, USA

Corresponding author: cpeacock@nova.edu

**Background:** Previous research demonstrated that the wrist-worn Apple Watch device was an accurate and reliable instrument of measuring heart rate during different physical testing. However, findings also demonstrated that the Apple Watch is not a reliable measurement for the assessment of energy expenditure during different physical testing. There is a lack of research examining the newest Apple Watch (Series 7) during VO2 max testing while using the metabolic cart (Trueone 2400) as the standard of measurement. Therefore, we proposed to explore the accuracy of maximal energy expenditure and maximum heart rate between the Apple Watch and the metabolic cart during a maximal aerobic capacity test.

**Methods:** 22 physically active adults (23.8 ± 4.0 years, 175.2 ± 10.4 cm, 73.8 ± 16.3 kg, 42.1 ± 8.3 mL/kg/min−1) completed the study. Subject height and weight were recorded using a stadiometer scale. The subjects were equipped with an Apple Watch Series 7, a polar heart rate monitor and o metabolic cart (ParvoMedics, Utah, USA). After the subjects provided consent, they were instructed to run a VO2 max test on the treadmill using the Bruce protocol. All subjects were monitored for maximal energy expenditure (MEE) and maximum heart rate (MHR) throughout the maximal aerobic capacity test. The collected data was analyzed via correlations analysis (SPSS 28). The study was approved by the University’s Institutional Review Board.

**Results:** All descriptive data including age, height, weight, and VO2 Max was calculated for the subjects. A paired samples t-test was conducted to compare the MEE and MHR using the Parvo metabolic cart with Polar function (PARVO) and the Apple Watch Series 7 (AW7) during a VO2 max test. There was not a significant difference in MEE between PARVO (M = 109.6, SD = 41.7) and AW7 (M = 98.7, SD = 24.4) conditions; t(21) = 1.5, p = 0.153. Additionally, there was not a significant difference in MHR between PARVO (M = 186.2, SD = 16.2) and AW7 (M = 189.3, SD = 8.5) conditions; t(21) = −0.9, p = 0.379.

**Conclusions:** The primary aim of this study was to gain a better understanding of the precision of the AW7 during VO2 Max testing, as it is one of the most popular wrist-worn devices to monitor physical activity. Contrary to our initial hypothesis, there appeared to be no difference between PARVO and AW7 during a maximal aerobic capacity test. The RER (≥ 1.1) indicated a true maximum test for the subject population.

**Binge Drinking: Implications for Female Resistance Trained Athletes**

Chester M. Sokolowski^a^, Jeong-Su Kim^a^, Sarah N. Perch^a^, Hunter J. Mitchell^a^, Jennifer L. Steiner^a^, Walter R. Boot^a^, and Michael J. Ormsbee^a^

^a^Florida State University, Tallahassee, FL, USA

Corresponding author: csokolowski@fsu.edu

**Background:** It is well established that resistance training (RT) can positively impact musculoskeletal and metabolic health. Contrarily, binge drinking (BD) can have harmful effects on musculoskeletal and metabolic health. The relationship among RT, BD, and various performance and health-related outcomes requires more attention given the high level of BD during the ages of 18-25 years. The aim of the present study was to determine how RT and BD impact musculoskeletal health, physical performance, and insulin sensitivity in young adult females.

**Methods:** Young adult females (age: 22.2 ± 2.5 years; BMI: 23.2 ± 3.0 kg/m2) were split into 4 groups: 1) sedentary low alcohol consumers (SL; n = 12), 2) sedentary binge drinkers (SB; n = 12), 3) resistance trained low alcohol consumers (RTL; n = 12), and 4) resistance trained binge drinkers (RTB; n = 12). To be classified as sedentary, women performed exercise ≤2 days per week for the past 2 years. To be classified as resistance trained, women performed RT ≥4 times per week for the past 2 years. To be classified as a low alcohol consumer, women consumed ≤1 alcoholic drink per week for the past year. To be classified as a binge drinker, women binge drank at least once per week for the past year. Binge drinking was defined as consuming ≥4 alcoholic drinks in about 2 hours. Women were assessed for musculoskeletal composition via dual-energy x-ray absorptiometry scan and ultrasonography. Physical performance was assessed through chair stand power, vertical jump, squat jump, broad jump, knee extensor torque, grip strength, and push-ups. Insulin sensitivity was assessed via an oral glucose tolerance test (OGTT) using capillary blood glucose. Values are reported as mean plus/minus standard deviation.

**Results:** Compared to sedentary females, resistance trained females had a lower body fat percentage (RTL = 29.3 ± 4.3%; RTB = 29.0 ± 3.7%; SL = 37.2 ± 5.3%; SB = 34.8 ± 3.5%; p < .05). RTL had more fat-free mass than SL and SB (RTL = 48.3 ± 5.8 kg; SL = 38.1 ± 6.6 kg; SB = 40.4 ± 6.1 kg; both p < .05). There were no differences between RTL and RTB on fat-free mass, fat mass, percent body fat, bone mineral density (BMD), muscle quality, or physical performance tests, and both groups trended to outperform or significantly outperformed SL and SB. RTL did have a lower fasting blood glucose than RTB (88.9 ± 12.1 mg/dL and 103.2 ± 10.2 mg/dL, respectively; p = .008), and RTL had lower blood glucose at 120 minutes of the OGTT than all groups (RTL = 103.8 ± 19.8 mg/dL; RTB = 137.6 ± 21.5 mg/dL; SL = 148.5 ± 44.6 mg/dL; SB = 139.3 ± 14.5 mg/dL; all p < .05).

**Conclusions:** These data suggest chronic BD does not deleteriously impact body composition, BMD, muscle quality, or physical performance in young adult females who participate in RT at least 4 times per week. However, even in young adult females who participate in habitual RT and have comparable body composition to low alcohol consumers, chronic BD does impair insulin sensitivity.

**Acknowledgments:** This study was supported by a Florida State University Doctoral Grant.

**Quality of Life and Early Sport Specialization Among Retired Collegiate and Professional Tennis Players**

Ecaterina Vasenin^a^, Jeffrey R. Stout^a^, David H. Fukud^a^

^a^Physiology of Work and Exercise Response Laboratory, School of Kinesiology and Physical Therapy, Institute of Exercise Physiology and Rehabilitation Science, University of Central Florida, Orlando, FL, USA

Corresponding author: david.fukuda@ucf.edu

**Background:** Early sport specialization has been associated with higher rates of injuries sustained by athletes during their professional/collegiate careers, which might negatively influence their quality-of-life following retirement from the professional/collegiate sport. The purpose of this study is to examine the relationships between early specialization in the sport of tennis and health outcomes following retirement from a collegiate/professional sport.

**Methods:** Participants were recruited via social media postings, newsletters, and contacts at tennis organizations. The survey was conducted via Qualtrics and included responses from 224 former tennis athletes. Sixty-six responses were removed from all analyses due to incomplete survey responses and/or not meeting the inclusion criteria. Thus, data from 158 retired athletes were considered. Basic demographic and injury information were collected along with the age at tennis specialization and two questionnaires, The Oslo Sports Trauma Research Center Questionnaire on Health Problems (OSTRC), and Healthy Days Measure Questionnaire (HRQOL). The OSTRC Questionnaire recorded the magnitude, symptoms and consequences of overuse injuries and illnesses that participants experienced in the last 7 days resulting in a current injury/illness severity score. Participants were divided into two low (scores of 0-50) and high (scores of 51-100) OSTRC groups with higher scores representing greater severity. The HRQOL Questionnaire measured physical and mental health preconceptions (e.g. energy level, social support, and socioeconomic status) specifically focusing on the sum of both physical and mentally unhealthy days experienced over the past 30 days. Participants were divided into high (scores of 0-15) and low (scores of 16-30) HRQOL groups.

**Results:** Significant differences (F1,138 = 9.476, p <.001) in specialization age between the low (11.9 ±4.459) and high (9.3 ±3.725) OSTRC groups were found after covarying for the current age. No difference (F1,138 = 0.056, p <.813) was shown among the high (10.3 ±4.197) and low (11.5 ±4.471) HRQOL groups for the specialization age after covarying for the current age. A weak positive correlation was identified between HRQOL and OSTRC scores (r = .238), while a weak positive correlation was shown for specialization age and OSTRC scores (r = −.215).

**Conclusions:** Retired tennis players with low injury/illness severity scores specialized in tennis later than those with high injury/illness severity scores, while no differences in specialization age were noted when the sample was separated into HRQOL groups. Despite there being a weak positive correlation between OSTRC and HRQOL scores only OSTRC appears to be related to specialization age.

**Efficacy of a Microalgae Extract Combined with Natural Guarana on Cognitive Performance of Gamers II: Sternberg Task Test**

Jonathan Maury^b^, Megan Leonard^a^, Broderick Dickerson^a^, Drew Gonzalez^a^, Jacob Kendra^a^, Tori Jenkins^a^, Kay Nottingham^a^, Choongsung Yoo^a^, Dante Xing^a^, Joungbo Ko^a^, Rémi Pradelles^a^, Ryan Sowinski^a^, Christopher J. Rasmussen^a^, Richard B. Kreider, FISSN^a*^.

^a^Exercise & Sport Nutrition Laboratory, Human Clinical Research Facility, Texas A&M University, College Station, TX 77843, USA; ^b^Microphyt, Research & Development Department, Baillargues, Mudaison, FR

Corresponding author: rbkreider@tamu.edu

**Background:** Competitive gaming requires visual selective attention, short-term memory or task switching, and an ability to sustain a high level of energy over time. Fucoxanthin is a major carotenoid, found in specific microalgae varieties like Phaeodactylum triconutum that has been reported to possess neuroprotective and nootropic effects through its anti-inflammatory and antioxidant activities on different signaling pathways like Nrf2-ARE. The purpose of this study was to evaluate whether acute and 30-day supplementation of a microalgae extract from Phaeodactylum triconortum with Guarana would affect cognitive function of gamers.

**Methods:** In a double-blind, placebo-controlled manner, 51 male and 10 female experienced gamers (21.7 ±4 years, 73.0 ±13 kg, 24.2 ±3.6 kg/m2) were randomly assigned to ingest a placebo (PL); low-dose (LD) of GamePhyt™ (MicroPhyt, Baillargues, FR) containing 440 mg/day of Phaeodactylum tricornutum extract including 1% Fucoxanthin + 440 mg/day of guarana, or high-dose (HD) of GamePhyt™ containing 2 × 440 mg/day of Phaeodactylum tricornutum extract including 1% Fucoxanthin + 440 mg/day of guarana for 30-days. Participants refrained from consuming atypical amounts of stimulants, food, and supplements that may affect cognition during the study. Acute (single dose) cognitive function tests were administered on Day 0 prior to supplementation, 15-min post-supplementation, and after the participants played their most competitive video game for 60-minutes. Participants continued supplementation for 30-days and then repeated pre-supplementation and post-gaming cognitive function tests. The battery of cognitive function tests included the Sternberg Task test (STT) which involves presenting participants with visual stimuli one at a time with the participant identifying them as either present or absent within sequences of at 3, 6, 9, 12, 15, or 18-second intervals. In order to prevent rehearsal, the participants were instructed to count backward in threes and fours to a specific random number until they saw a red light appear on the computer screen. This test measures short-term/working memory involving cognitive control processes, using reaction time and accuracy. Data were analyzed by General Linear Model (GLM) univariate analyses with repeated measures using weight as a covariate and mean and percent changes from baseline with 95% confidence intervals.

**Results:** Results revealed that acute LD and HD ingestion reduced pre-game and post-game reaction times with 2 letter length tasks and that post-gaming present reaction times were faster with HD as the complexity of tasks increased. There was also evidence that 30-days of HD ingestion promoted faster present reaction times in 4 letter tasks.

**Figure 1.**
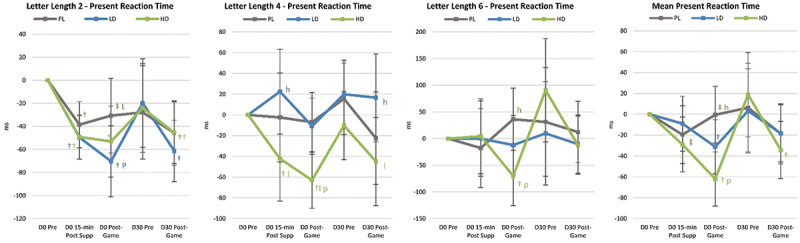
Chang in Sternberg Task Test. Data are means and 95% confidence intervals. Changes from baselind are show as † (p<0.05 change from baseline) and ‡ (p<0.05 to p<0.10 trends from baseline). Small case letters indicate p<0.05 differences from placebo (pl), low dose (ld), or high dose (hl) whileupper-case letters (PL, LD, HD) indicate trends (p<0.0-5 to p<01.10).

**Conclusions:** Results provide some evidence that acute and chronic supplementation with a microalgae extract from Phaeodactylum triconutum with Guarana can affect short-term/working memory involving cognitive control processes, using reaction time and accuracy. There is evidence that acute LD and HD supplementation improved post-game present reaction time with HD supplementation having more consistent results as the complexity of letter length challenges increased.

**Acknowledgments:** This study was funded by MicroPhyt (Baillargues, FR) as a fee-for-service project to the Human Clinical Research Facility at Texas A&M University and conducted by the Exercise & Sport Nutrition Lab.

**Efficacy of a Microalgae Extract Combined with Natural Guarana on Cognitive Performance of Gamers I: Card Sorting Task Test**

Megan Leonard^a^, Jonathan Maury^b^, Broderick Dickerson^c^, Drew Gonzalez^a^, Jacob Kendra^a^, Tori Jenkins^a^, Kay Nottingham^a^, Choongsung Yoo^a^, Dante Xing^a^, Joungbo Ko^a^, Rémi Pradelles^b^, Ryan Sowinski^a^, Christopher J. Rasmussen^a^, Richard B. Kreider, FISSN^a*^

^a^Exercise & Sport Nutrition Laboratory, Human Clinical Research Facility, Texas A&M University, College Station, TX, USA; ^b^Microphyt, Research & Development Department, Baillargues, Mudaison, FR

Corresponding author: rbkreider@tamu.edu

**Background:** Competitive gaming requires visual selective attention, short-term memory or task switching, and an ability to sustain a high level of energy over time. Fucoxanthin is a major carotenoid, found in specific microalgae varieties like Phaeodactylum triconutum that has been reported to possess neuroprotective and nootropic effects through its anti-inflammatory and antioxidant activities on different signaling pathways like Nrf2-ARE. The purpose of this study was to evaluate whether acute and 30-day supplementation of a microalgae extract from Phaeodactylum triconortum with Guarana would affect cognitive function of gamers.

**Methods:** In a double-blind, placebo-controlled manner, 51 male and 10 female experienced gamers (21.7 ±4 years, 73.0 ±13 kg, 24.2 ±3.6 kg/m2) were randomly assigned to ingest a placebo (PL); low-dose (LD) of GamePhyt™ (MicroPhyt, Baillargues, FR) containing 440 mg/day of Phaeodactylum tricornutum extract including 1% Fucoxanthin + 440 mg/day of guarana, or high-dose (HD) of GamePhyt™ containing 2 × 440 mg/day of Phaeodactylum tricornutum extract including 1% Fucoxanthin + 440 mg/day of guarana for 30-days. Participants refrained from consuming atypical amounts of stimulants, food, and supplements that may affect cognition during the study. Acute (single dose) cognitive function tests were administered on Day 0 prior to supplementation, 15-min post-supplementation, and after the participants played their most competitive video game for 60-minutes. Participants continued supplementation for 30-days and then repeated pre-supplementation and post-gaming cognitive function tests. The battery of cognitive function tests included the Berg-Wisconsin Card Sorting Task test (BCST). The BCST involves participants being presented with visual stimuli (i.e. pictures of playing cards) with instructions to sort the cards by matching colors and/or designs. The test assesses reaction time and accuracy in measuring reasoning, learning, executive control, attention shifting by assessing the inability to shift set (i.e. display flexibility in the face of changing schedules of reinforcement), and impulsiveness. Data were analyzed by General Linear Model (GLM) univariate analyses with repeated measures using weight as a covariate and mean and percent changes from baseline with 95% confidence intervals.

**Results:** Results are shown in Figure 1. Results revealed that errors decreased significantly in PL but not LD or HD. There was also some evidence that participants ingesting the HD treatment had greater correct responses than LD and that HD supplementation significantly reduced perseveration errors (PEBL) and perseveration errors with PAR rules, whereas the LD had higher errors than PL.

**Figure 1.**
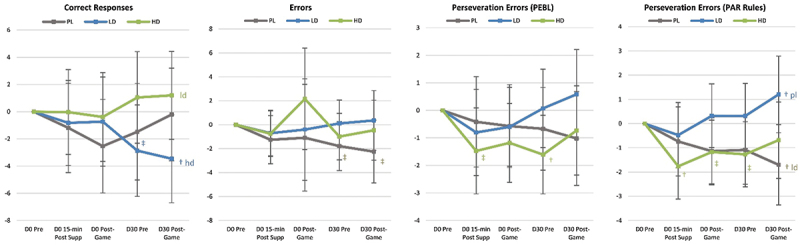
Changes in Berg-Washington Card Sorting Task Tests. Data are means and 95% confidence intervals. Changes from baselind are show as † (p<0.05 change from baseline) and ‡ (p<0.05 to p<0.10 trends from baseline). Small case letters indicate p<0.05 differences from placebo (pl), low dose (ld), or high dose (hl) whileupper-case letters (PL, LD, HD) indicate trends (p<0.0-5 to p<01.10).

**Conclusions:** Results provide some evidence that acute and chronic ingestion of microalgae extract from Phaeodactylum triconutum with Guarana can affect reasoning, learning, executive control, and attention shifting by enhancing flexibility in the face of changing schedules of reinforcement, and impulsiveness. While some acute effects were noted, the greatest impact appeared to be after 30-days.

**Acknowledgments:** This study was funded by MicroPhyt (Baillargues, FR) as a fee-for-service project to the Human Clinical Research Facility at Texas A&M University and conducted by the Exercise & Sport Nutrition Lab.

**A Pilot Study to Determine the Bioavailability of Orally Ingested Beta-Aminoisobutyric Acid (BAIBA) Supplement**

Joesi M. Krieger^a^, Logan S. Orr^a^, Anthony M. Hagele^a^, Kylie E. Gross^a^, Connor J. Gaige^a^, Kyle L. Sunderland^a^, Petey W. Mumford^a^, Chad M. Kerksick^a^

^a^Exercise and Performance Nutrition Laboratory, College of Science, Technology, and Health, Lindenwood University, St. Charles, MO, USA

Corresponding author: ckerksick@lindenwood.edu

**Background:** L-beta-amino isobutyric acid (L-BAIBA) is a myokine produced in skeletal muscle during exercise and has been shown to impact carbohydrate and fat metabolism in both animals and humans. This study was designed to determine the rate and extent to which L-BAIBA appeared in human plasma after oral ingestion of a single 250 mg (B250), 500 mg (B500), and 1,500 mg (B1500) dose of L-BAIBA and 1,500 mg (V1500) dose of L-valine.

**Methods:** In a randomized, double-blind, placebo-controlled, crossover fashion, 12 males and females (M/F = 6/6; 24 ±5 yrs; 173.6 ±12.0 cm; 72.3 ±11.3 kg; 21.0 ±7.0 % fat) completed a single-dose supplementation protocol of placebo (PLA), B250, B500, B1500, and V1500. Participants fasted overnight (8–10 hours) and consumed their dose with 8-12 fluid ounces of cold water. Venous blood samples were collected 0, 30, 60, 90, 120, 180, 240 and 300 minutes (min) after ingestion and analyzed for L-BAIBA. Complete blood counts and comprehensive metabolic panels were analyzed 0 and 300 minutes after ingestion. Peak concentration (CMax) and area under the curve (AUC) were calculated for all variables.

**Results:** Baseline L-BAIBA levels were not different between conditions (p = 0.46). The observed AUC for B1500 (30,513 ±9190 µM•300 min) was significantly higher than B500 (11,087 ±3378 µM•300 min, p <0.001), B250 (7081 ±2535 µM•300 min, p <0.001), V1500 (2837 ±2107 µM•300 min, p <0.001), and PLA (2836 ±2061 µM•300 min, p <0.001). Similarly, L-BAIBA CMax for B1500 (278.1 ±52.1 µM) was significantly higher than all other supplement conditions: B500 (95.4 ±33.5 µM, p <0.001), B250 (63.3 ±61.1 µM, p <0.001), V1500 (10.1 ±7.2 µM, p <0.001), PLA (11.0 ±7.1 µM, p = 0.001). AUC and CMax for B500 was significantly higher than B250 (p <0.001), V1500 (p <0.001), and PLA (p <0.001). L-BAIBA AUC for B250 was significantly higher than V1500 (p <0.001) and PLA (p <0.001). No clinically significant changes in blood-based markers of health or adverse events were observed across the study protocol.

**Conclusions:** L-BAIBA doses of 1500 mg, 500 mg, and 250 mg produced significantly greater concentrations of plasma L-BAIBA across a five-hour measurement window when compared to a 1500 mg dose of valine or a placebo. Follow-up efficacy studies on resting and exercise metabolism should be completed to assess the impact of L-BAIBA supplementation in normal weight and overweight individuals.

**Acknowledgments:** This study was funded by NNB Nutrition.

**Sleep Quality, Social Jet Lag and Cardiovascular Risk in Young Adults**

Daniel Coimbra Amorim^a^, Sabrina Amabili Marinho Teles^b^

^a^Coimbra Academy, LTDA, CE, Brazil^b^Coimbra Academy, LTDA, CE, Brazil

Corresponding author: danielcoimbrapaciente@gmail.com

**Background:** The aim of the article is to associate sleep quality and cardiovascular risk factors in university students. In recent years, the incidence of cardiovascular diseases (CVD) has been associated with lifestyle, such as diet, physical activity, stress and sleep. The deprivation of the sleep is an important point to understanding how this can increase the risk of CVD, especially in young adults, who are more affected by sleep quality.

**Methods:** This is an observational study with 98 university students from public and private institutions in Fortaleza. Sociodemographic, economic, lifestyle data, food frequency questionnaire, family history, anthropometric data, blood pressure, biochemical parameters, and sleep questionnaires (Pittsburgh Sleep Quality Index and Munich Chronotype Questionnaire – MCTQ) were collected.

**Results:** The results showed that excess of weight was associated with worst sleep quality scores (15,7 +5,0, p <0.05), higher levels of CPR (ρ = 0.437, p <0.05) and %HbA1c (ρ = 0.377, p <0.05). It was found a significant association between worse sleep quality scores and BMI (ρ = 0.205, p <0.05), triglycerides (ρ = 0.202, p <0.05) and CRP (ρ = 0.302, p <0.05). No significant associations were found between sleep quality and blood pressure, waist circumference, total cholesterol, fasting glucose, HDL-c and LDL-c.

**Conclusions:** It was concluded there is a close association between sleep quality and biochemical changes related to cardiovascular disease, being overweight an aggravating factor.

**Acknowledgments:** No conflict of interest

**Cordyceps Militaris Supplementation: Effect of Dosage on Skeletal Muscle Performance**

Michael J. Webster^a^, Benjamin R. Davis^a,b^, Tijana Simovic^a,c^, Chandler R. Bridges^a,d^, Liliana I. Rentería^e^, Shiloah A. Kviatkovksy^e^, Casey E. Greenwalt^e^, Michael J. Ormsbee^e^

^a^Valdosta State University, Valdosta, GA, USA^b^Kent State University, Kent, OH, USA^c^Virginia Commonwealth University, Richmond, VA, USA^d^Andrews Research and Education Foundation, Gulf Breeze, FL, USA^e^Florida State University, Tallahassee, FL, USACorresponding author: MJWebster@Valdosta.edu

**Background:** Cordyceps have been used as an herbal Chinese medicine for centuries. While there are a variety of cordyceps species, recently there has been an increased attention to the *Cordyceps militaris* (CM) species. CM has been shown to have a very high cordycepin content which has been suggested to have a very potent antioxidant action. There are relatively few studies investigating CM and exercise; however, a CM blend has been demonstrated to significantly delay fatigue and reduce oxidative stress in swimming mice, and to increase peak oxygen uptake in recreationally active individuals. Considering the antioxidant and anti-fatigue properties attributed to CM, the aim of this study was to investigate the effect of three different dosages of CM supplementation for seven days on skeletal muscle peak torque and work output in a recreationally active population.

**Methods:** Recreationally active males and females volunteered to participate in the study which employed a randomized, double-blind, placebo-controlled design. All participants were supplemented for 7 full days with either a placebo (cellulose) or CM dosage of 1-, 2-, or 4-grams·d−1. Supplements were provided in identically colored gelatin capsules. Participants were requested to continue their normal dietary habits through the duration of the study. A Biodex Isokinetic Dynamometer was utilized to assess maximal muscular torque and work output of the biceps brachii of the non-dominant arm on day-0 (Pre) and after 7-d (Post) supplementation. Participant performed each of the following assessments: 1) Isometric maximum (IMM), 2) Isokinetic three repetition maximum (IK3RM) at 120°·s−1, and 3) Isokinetic twenty repetition maximum (IK20RM) 120°·s−1. Data are reported as mean ± SE and statistical significance set at p <.05).

**Results:** 33 participants (n = 20 males and 13 females; age: 22.5 ± 0.5 y; height: 1.74 ± 0.02 m; weight: 78.21 ± 3.22 kg; body fat: 22.2 ± 1.40%) completed the study (n in each group: 0-g∙d−1 = 7, 1-g·d−1 = 8, 2-g·d−1 = 10, 4-g·d−1 = 8). Repeated measures ANOVA revealed no statistically significant differences between groups for IMM peak torque (p >.05), IK3RM peak torque or work (p >.05), or IK20RM work (p >.05).

**Conclusions:** While CM has purported antioxidant and anti-fatigue properties, the findings of the present study indicate that, irrespective of 7-d dosage, CM does not significantly impact maximal isometric and isokinetic peak torque and/or work output. Further study of the impact of CM supplementation during different types and/or intensities of exercise, as well as muscle recovery, are warranted.

**Acknowledgments:** None of the authors has any real or apparent conflicts of interest. This project was financially supported via a VSU Faculty Research Seed Grant and the *Cordyceps militaris* was generously provided by NAMMEX WWW.NAMMEX.COM

**Effect of Oral Creatine Supplementation on Physical Performance and Quality of Life in a Subject with Post-polio Syndrome**

Greg E Popovich^a^

^a^School of Exercise Science & Athletic Training, West Virginia Wesleyan College, Buckhannon, WV, USA

Corresponding author: popovich.g@wvwc.edu

**Background:** Creatine has been proposed as a potential adjuvant therapy for many types of neuromuscular disease, including post-polio syndrome (PPS). PPS, like other neuromuscular conditions, is characterized by low endogenous creatine levels. This case report is unique with regard to creatine supplementation for PPS in that it describes (a) a relatively extended creatine supplementation regimen; (b) a bilateral comparison of muscle function to measure potential differences in magnitude of effect between asymmetrically affected limbs in a given individual; and (c) an attempt to determine if creatine’s ergogenic effects extend beyond the laboratory setting to impact quality of life.

**Methods:** The subject was a 44-year-old female vegetarian. Intervention began 10 days status-post acute flare-up of PPS symptoms with no concurrent therapies nor changes in diet or activity for the duration of the 30-day trial. The subject consumed 5 g servings of supplemental creatine as follows: 10 g daily for 10 days and 5 g daily for an additional 20 days (= 200 g total supplemental creatine). Muscle performance measures were performed at days 0, 10, and 30 and included isometric grip dynamometry and isokinetic lower extremity dynamometry. Health-related quality of life (HRQL) was assessed at the same time points using the Short Form 36 Health Survey (SF-36).

**Results:** The subject reported 100% adherence to the supplementation regimen. The patient did not report any increase in muscle cramping or fasciculations during the intervention period. There was no meaningful change in the patients’ bilateral grip strength. Lower extremity isokinetic test results revealed an approximately 15% performance increase in both knee flexion and extension on the unaffected side, but no such change was observed in the symptomatic limb. The patient’s SF-36 did not indicate improvement in HRQL.

**Conclusions:** This case report suggests that creatine supplementation may be well-tolerated in post-polio patients without exacerbation of muscular cramping and fasciculations. Despite a substantial rationale for creatine supplementation in this specific case owing to several predictors of low creatine stores (i.e. neuromuscular disease in a female vegetarian), there was no evidence of improvement in any of the qualitative or quantitative measures with the exception of typical isokinetic improvement in the unaffected lower extremity. The latter suggests that the subject was not merely a non-responder, but rather that the symptomatic versus asymptomatic limbs responded differently to creatine supplementation.

**Weight Loss in Professional Mixed Martial Artists 24 Hours Prior to Official Weigh-In**

Gabriel J. Sanders^a^, Anthony Ricci^b^, Charles Stull^c^, Duncan French^c^, Jose Antonio^b^, Corey A. Peacock^b^

^a^Department of Kinesiology, Northern Kentucky University, Highland Heights, KY, USA^b^Department of Health and Human Performance, Nova Southeastern University, Davie, FL, USA^c^UFC Performance Institute, Las Vegas, NV, USA

Corresponding author: cpeacock@nova.edu

**Background:** Previous research has shown that professional mixed martial artists (MMA) utilize a variety of strategies to manage and lose weight prior to officially weighing in for competition. Although there is much literature demonstrating weight loss and methods, minimal research exists analyzing how much weight professional MMA lose 24 hours prior to the official weigh-in. Therefore, the purpose of the current study is to compare professional MMA weight 24 hours prior versus official weigh-in.

**Methods:** 1034 professional MMA fighters (30.1 ± 3.9 yrs.; 176.8 ± 9.1 cm) competing for the Ultimate Fighting Championship between 2020 and 2022 were used for the study. The athletes reported to the assigned work-out rooms 24 hours prior to official weigh-ins. Weight was obtained using a commission calibrated digital scale. The following day, an official weigh-in took place where weight was obtained utilizing the official commission managed beam scale. Paired Samples T Tests were utilized, and significance was set at P ≤ 0.05.

**Results:** There is a significant (P <.001) weight difference within 24 hours prior to official weigh-in (Table 1).

**Table 1.** Weigh-in vs fight weight changes.

	24 Hours Pre (kg)	Official Weigh-In (kg)	% Change	P-Value
N = 1034:	75.2 ± 15.6	71.7 ± 15.6	−4.8 ± 2.6	P <.001

M±SD, *Significance set at P ≤ 0.05

**Conclusions:** MMA athletes decrease body weight significantly 24 hours prior to official weigh-ins. Based on this data, it appears athletes average a weight loss of nearly 5% prior to weigh-ins. Further data is being analyzed to better explain weight loss strategies in professional MMA.

**Weigh-In versus Fight-Weight in Professional Mixed Martial Artists**

Corey A. Peacock^a^, Gabriel J. Sanders^b^, Anthony Ricci^a^, Charles Stull^c^, Duncan French^c^, Jose Antonio^a^

^a^Department of Health and Human Performance, Nova Southeastern University, Davie, FL, USA^b^Department of Kinesiology, Northern Kentucky University, Highland Heights, KY, USA^c^UFC Performance Institute, Las Vegas, NV, USA

Corresponding author: cpeacock@nova.edu

**Background:** Previous research has demonstrated that professional mixed martial artists (MMA) employ a variety of weight manipulation strategies in order to compete at a given weight-class. Although there is much literature demonstrating weight manipulation methods, minimal research exists analyzing how much weight professional MMA gain between the official weigh-in and competition. Therefore, the purpose of the current study is to compare the official weigh-in and fight-weight in professional MMA.

**Methods:** 1047 professional MMA fighters (30.1 ± 3.9 yrs.; 176.8 ± 9.1 cm) competing for the Ultimate Fighting Championship between 2020 and 2022 were used for the study. The athletes reported to the arena for an official weigh-in (24-36 hours prior to competition). An official weight was obtained utilizing the commission managed beam scale. The following day, athletes return to the arena for competition and weight is obtained using a commission calibrated digital scale. Paired Samples T Tests were utilized, and significance was set at P ≤ 0.05.

**Results:** There is a significant (P <.001) difference between weigh-in and fight-weight in professional MMA (Table 1).

**Table 1.** Weigh-in vs fight weight changes.

	Official Weigh-In (kg)	Fight-Weight	% Change	P-Value
N = 1047:	73.0 ± 16.8	79.8 ± 16.8	9.8 ± 4.0	P <.001

M±SD, *Significance set at P ≤ 0.05

**Conclusions:** MMA athletes increase body weight significantly following official weigh-ins. Based on this data, it appears athletes average a weight gain of nearly 10% between official weigh-in and competition. Further data is being analyzed to better explain rehydration strategies in professional MMA.

**Is Your Home Body Fat Scale Lying to You? Assessing the Longitudinal Validity of 15 Bioelectrical Impedance Analysis Devices**

Madelin R. Siedler^a^, Christian Rodriguez^a^, Matthew T. Stratton^a^, Patrick S. Harty^a^, Dale S. Keith^a^, Jacob J. Green^a^, Jake R. Boykin^a^, Sarah J. White^a^, Abegale D. Williams^a^, Brielle DeHaven^a^, & Grant M. Tinsley^a^

^a^Kinesiology and Sport Management, Texas Tech University, Lubbock, TX, USA

Corresponding author: grant.tinsley@ttu.edu

**Background:** Bioelectrical impedance analysis (BIA) is a popular method of body composition assessment used in clinical, field, consumer, and research contexts. A wide variety of consumer-grade BIA devices is available, but the validity of these devices for tracking changes in body composition over time is unclear.

**Methods:** Thirty-seven healthy participants (16 females and 21 males; mean±SD age: 28.6 ±8.2 years; height: 168.7 ±8.6 cm; weight: 72.1 ±14.5 kg) were included in this analysis. Participants visited the laboratory on two occasions scheduled 12-16 weeks apart. At each visit, the body fat percentage (BFP) was recorded from 15 BIA devices: 14 consumer-grade devices and one research-grade device. These models included 10 foot-to-foot devices, four octapolar (hand-to-foot, bilateral) devices, and one hand-to-hand device. Participants were not specifically instructed to change any aspect of their habitual lifestyle, but were told that they could lose, gain, or maintain weight between visits. The change in BFP values between visits as estimated by each of the 15 BIA devices was compared to the changes observed via a laboratory four-compartment (4C) criterion model. The 4C model included body mass from a calibrated scale, body volume from air displacement plethysmography, bone mineral content from dual-energy x-ray absorptiometry, and total body water from bioelectrical impedance spectroscopy. Longitudinal validity was assessed using Lin’s concordance correlation coefficient (CCC), constant error (CE), and the slope and statistical significance of the linear regression line on the Bland-Altman plot.

**Results:** The mean change in the 4C model was +0.1 ±2.9% body fat, with a range from −5.0% to +7.2% across individuals. The mean change across the 15 BIA devices ranged from −0.2 ±2.3% to +1.4 ±3.6%. Across devices, Lin’s CCC ranged from 0.38 to 0.78, CE ranged from −0.3% to 1.3%, and the Bland-Altman slope ranged from −0.96 to 0.25. The Bland-Altman slope was significantly different from zero in the seven (47%) devices with the greatest negative proportional bias. Across all metrics, octapolar models generally performed among the top half of devices, whereas a subset of foot-to-foot devices consistently demonstrated the poorest performance.

**Conclusions:** Commercially available BIA devices vary in terms of their ability to detect changes in BFP over time. However, some models exhibited longitudinal validity comparable to that of the research-grade device within our sample. Thus, select models within this class of devices may potentially be useful for the tracking of body composition changes over time, particularly within consumer and field contexts.

**Acknowledgments:** The authors declare no sponsors or relevant conflicts of interest.

**The Effects of Bioimpedance Spectroscopy Electrode Distance Placement on Body Composition and Total Body Water Estimates in Recreationally Trained Participants**

Andrew Andraos^a^, Michael Torres^a^, Daniela Ornelas^a^, Jessica Heredia^a^, Alexandra Khartabil^a^, Mariesha Islas^a^, Grant Tinsley^b^, Guillermo Escalante^a^

^a^Department of Kinesiology, California State University San Bernardino, San Bernardino, CA, USA^b^Department of Kinesiology & Sport Management, Texas Tech University, Lubbock, TX, USA

Corresponding author: gescalan@csusb.edu

**Background:** This study investigated the effects of bioimpedance spectroscopy electrode distance placement on estimates of total body water (TBW) and body fat percentage (BF).

**Methods:** Eighteen resistance-trained males (n = 6) and females (n = 12) (Age: 26.7 ± 7.7 years; 166.7 ± 9.1 cm; 68.8 ± 15.1 kg) were recruited. Participants arrived to the lab in the morning following best standardized practice protocols (fasted and non-exercised for 12 hours). Participants were placed in supine position on a table and two electrodes were placed on the right midline of the ulnar styloid process and the distal electrode was placed 5 cm below; the other two electrodes were placed between the medial and lateral malleoli and the distal electrode was placed 5 cm below. Two measurements for BF and TBW were collected. After the first two measurements, electrodes were placed an additional 0.5 cm above and below the original locations and re-measured for TBW and BF. Paired sample T-Tests were used to determine if the TBW and BF estimates were different between the different electrode placements (5 cm-1 vs 5 cm-2, 6 cm-1 vs 6 cm-2, Mean 5 cm vs Mean 6 cm).

**Results:** BIS TBW 5 cm-1 (37.58 ± 6.96 L) versus BIS TBW 5 cm-2 (37.53 ± 6.95 L) was not statistically significant (p = 0.102). BIS TBW 6 cm-1 (38.72 ± 7.34 L) vs BIS TBW 6 cm-2 (38.73 ± 7.33 L) was not statistically significant (p = 0.469). However, there was a statistically significant difference (p < 0.001) between BIS TBW 5 cm mean (37.55 ± 6.96 L) vs BIS TWB 6 cm mean (38.72 ± 7.34 L). Similarly, BIS BF 5 cm-1 (23.90 ± 9.34 %BF) vs BIS BF 5 cm-2 (23.89 ± 9.35 %BF) cm was not statistically significant (p = 0.341). BIS BF 6 cm-1 (21.53 ± 9.74 %BF) vs BIS BF 6 cm-2 (21.53 ± 9.74 %BF) was not statistically significant (p = 0.493). However, there was a statistically significant difference between BIS BF 5 cm mean (23.89 ± 9.34 %BF) vs BIS BF 6 cm mean (21.53 ± 9.74 %BF).

**Conclusions:** Improper placement of electrodes (0.5 cm from original position) resulted in statistically significant differences in TBW and BF. Technical errors can result in inaccurate measurements of TBW and BF and can lead to inaccurate reporting of the results from exercise and/or nutrition interventions.

**A Comparison of Body Composition Measurements with Best Practice Pre-test Conditions and Controlled Non-best Practice Pre-test Conditions using DXA, BIS, Skinfolds, US, and the 4C model in Recreationally Trained Males and Females**

Michael Torres^a^, Jessica Heredia^b^, Alexandra Khartabil^b^, Andrew Andraos^a^, Daniela Ornelas^a^, Mariesha Islas^a^, Jacob Echols^a^, Grant Tinsley^c^, Guillermo Escalante^a^

^a^Department of Kinesiology, California State University San Bernardino, San Bernardino CA, USA^b^Department of Kinesiology, California State University Fullerton, Fullerton CA, USA^c^Department of Kinesiology & Sport Management, Texas Tech University, Lubbock, TX, USA

Corresponding author: gescalan@csusb.edu

**Background:** The study compared body composition measurements with best practice pretest conditions and controlled non-best practice pretest conditions using dual x-ray absorptiometry (DXA), bioimpedance spectroscopy (BIS), skinfolds (SF), a-mode ultrasound (US), and the four- compartment model (4C) model in recreationally trained males and females.

**Methods:** Eighteen resistance-trained males (n = 6) and females (n = 12) (Age: 26.7 ± 7.7 years; 166.7 ± 9.1 cm; 68.8 ± 15.1 kg) were recruited. On day 0, participants logged all activities including food, fluid, and exercise. Participants arrived at the lab on day 1, the next morning, following best-standardized practice protocols (fasted and non-exercised for 12 hours). Height, weight, and specific gravity of urine were measured. Participants were measured with 4 separate methods: DXA, BIS, SF, and US. Participants continued to log their activity and returned to the lab 4-7 hours later to repeat the same testing protocol in a non-best practice state. Continuing to log their activity, individuals did not report to the lab for testing on day 2, but were instructed to repeat their logged activities from day 0. Individuals reported back to the lab for re-testing on day 3, repeating the procedures/activities/food log from day 1. Separate one-way repeated measures ANOVAs were used to determine if the BF% estimates were different between time points of Day 1 AM vs Day 1 PM, Day 3 AM vs Day 3 PM, Day 1 AM vs Day 3 AM, and Day 1 PM vs Day 3 PM.

**Results:** There were no significant differences in BF% between the testing times for the BIS (p = 0.223), US (p = 0.060), SF (p = 0.375), DXA (p = 0.066), and 4C (p = 0.397). However, there was a significant difference in TBW (p = 0.011) between the four-time points as measured by BIS. Post hoc tests revealed these differences were between Day 1 AM (37.1 ± 1.7 L) vs Day 1 PM (38.0 ± 1.7 L), p = 0.012 and Day 1 PM (38.0 ± 1.7 L) vs Day 3 AM (37.1 ± 1.7 L), p = 0.004.

**Conclusions:** Contrary to previous studies, body composition measurements were not significantly affected by following an unstandardized best practice protocol when compared to testing in a standardized best practice protocol.

**The Effects of Foot wear on Joint Kinematics and Muscle Electromyographical Activity During the Back Squat: a Case Study**

Venkata Naga Pradeep Ambati^a^, Jacob Echols^a^, Andrew Andraos^a^, Michael Torres^a^, Daniela Ornelas^a^, Alexandra Khartabil^a^, Dillon Darrow^a^,Gustavo Lua^a^, Rodolfo Mejia^a^, Guillermo Escalante^a^

^a^Department of Kinesiology, California State University San Bernardino, San Bernardino, CA, USA

Corresponding author: gescalan@csusb.edu

**Background:** This case study investigated the effects of foot wear on joint kinematics and muscle electromyographical (EMG) activity during the back squat.

**Methods:** One resistance-trained male (Age: 26; height = 167.6 cm; weight = 80 kg) with 6 years of weightlifting experience was recruited. EMG electrodes were placed on the right vastus lateralis (VL), rectus femoris (RF), semitendinosus (ST), gluteus maximus (GM), and lumbar erector spinae (ES). Motion sensors were also placed on the occiput, inferior to C7, L1, sacrum, and right femur/lower shank/foot. A Noraxon EMG/myomotion system (Noraxon, Phoenix, AZ) was used to capture/analyze the data. After electrode placement/calibration, maximum isometric voluntary contraction (MVIC) for the RF, VL, ST, GM, and ES were measured. The participant then performed 2 submaximal sets of back squats before loading the bar to 80% of their self-reported 1 RM. In random order, the participant performed 3 full range of motion back squats with running shoes (RS), weightlifting shoes (WS), and barefoot (BF) with 5 minutes of rest between conditions. Range of motion (ROM) for the hip, knee, ankle, and trunk lean were measured along with EMG activity of the RF, VL, ST, GM, and ES. Data is presented as mean degrees of ROM and % MVIC for each muscle group for the 3 squat repetitions during the eccentric phase of the back squat.

**Results:** All results are presented in the following order: BF, RS, and WL, respectively. Hip flexion was 93°, 78.9°, and 84.7° for WT. Hip abduction was 12.3°, 38.5°, and 29.3°. Knee flexion was 77.3°, 78.9°, and 75.1°. Knee abduction was −9.2°, −3.2°, and −5.9°. Ankle dorsiflexion was 29.7°, 24.3°, and 27.3°. Trunk lean was 37.9°, 36.8°, and 34.9°. EMG for the RF was 129.2, 84.3, and 83.0 %MVIC. EMG for the VL was 37.6, 37.5, and 34.3 %MVIC. EMG for the ST was 82.2, 38.4, and 39.9 %MVIC. EMG for the GM was 70.2, 66.9, and 69.1 %MVIC. EMG for the ES was 21.9, 19.2, and 21.0 %MVIC.

**Conclusions:** Type of foot wear or squatting barefoot appears to alter joint kinematics and muscle EMG activity. More trunk lean, less hip abduction, and less knee abduction (more knee adduction) is apparent in the BF condition as compared to wearing shoes. More EMG activity of the RF and ST is apparent in the BF condition as compared to wearing shoes.

**Collagen Peptide Supplementation Improves Mental Component Scores of the VR-12 in Active Adults**

Shiloah A. Kviatkovsky^a,b,c^, Robert C. Hickner^a^, Stephanie D. Gipson^a,b,d^, Hannah E. Cabre^a,b,e^, Brett R. Hanna^a,b^, Haylee G. Colannino^a,b^, Kathryn E. O’Connor^a,b^, Anna S. Hayward^f^ and Michael J. Ormsbee^a,b^

^a^Department of Nutrition and Integrative Physiology, Florida State University, Tallahassee, FL, USA^b^Institute of Sports Sciences and Medicine, Florida State University, Tallahassee, FL, USA^c^Center for Aging and Longevity, Geriatrics, University of Arkansas for Medical Sciences, Little Rock, AR, USA^d^Faculty of Kinesiology & Physical Education, University of Toronto, Toronto, ON, Canada^e^Department of Sport Science, Applied Physiology Lab, The University of North Carolina at Chapel Hill, Capel Hill, NC, USA^f^Florida State University School of Medicine, Tallahassee, FL, USA

Corresponding author: skviatkovsky@uams.edu

**Background:** Chronic pain afflicts 19% of the U.S. population, with increasing incidence in active and aging populations. Pain can limit physical activity and activities of daily living, resulting in declined mental and social health. Pharmacological interventions have negative side effects, necessitating alternative therapies. Currently, nutritional interventions for pain target inflammation or joint health, but few influence both. Collagen, the most abundant protein in the human body and major constituent of the extra cellular matrix, is such a nutraceutical. While there are reports in short term studies of reductions in pain in response to collagen peptide (CP) supplementation, there have been no investigations of the effect of long-term CP supplementation on mental health related quality of life in healthy middle-aged active populations.

**Methods:** To determine the effects of daily CP consumption over 9 months on the Veterans Rand 12 (VR-12) mental components score (MCS) in active adults, participants (N = 41) were randomized into 3 groups in this double-blind study: 20 g/d (n = 13; male = 7), 10 g/d (n = 13; male = 5) of CP supplementation, or placebo (n = 15; male = 8). Participants were assessed at baseline, 3 months, 6 months, and 9 months. Mixed model ANOVAs assessed group by time interactions in VR-12 scores across all four time points. Significant interactions were further assessed via simple repeated measures ANOVA using Bonferroni corrections. Increasing values in VR-12 scores reflect improvements in MCS.

**Results:** A significant interaction between CP dose, across time points (baseline, 3 months, 6 months, and 9 months) in MCS was observed, F(6, 111) = 2.685, p = .027, partial η2 = .127, from baseline to 9 months. Follow-up within group one-way repeated measures ANOVA revealed a significant increase in MCS scores over time in the 10 g/d group, F(3, 36) = 5.371, p = .017, partial η2 = .309, with significant increases from baseline to 3 months, M = −9.806, SE = 3.00, p = .04. No significant main effects for time on MCS was observed within the 20 g/d, F(3, 33) = .975, p = .381, partial η2 = .081, or the placebo groups, F(3, 42) = 3.255, p = .031, partial η2 = .189, when using a Bonferroni correction (p = .017).

**Conclusions:** Daily CP intake of 10 g/day for 3 months improved MCS, which remained to the 9-month study endpoint. These findings suggest CP supplementation may play a role in improving mental health related quality of life in middle-age lifelong physically active adults.

**Acknowledgments:** PB Leiner, part of Tessenderlo Group, funded this study. The ISSM graduate and undergraduate student interns.

**Neuromuscular Function During Isometric Deadlifts with Conventional Versus Hexagonal Barbells**

Megan E. Bodden^a^, Jenna M. Bloch^a^, Karen L. Starnes^a^, Ryan M. Girts^a^, Matt S. Stock^a^

^a^University of Central Florida, Orlando, FL, USA

Corresponding author: matt.stock@ucf.edu

**Background:** The deadlift is a popular compound movement that allows lifters seeking to improve strength and power to load large muscle groups. Deadlifts and deadlift-based assessments are becoming common in sport performance and rehabilitation settings but have not been well studied. A common assumption is that since the hexagonal barbell shifts the external load posteriorly to be in line with the center of mass, the motion will cause greater activity of the quadriceps muscle group and decrease the stress on the erector spinae compared to a conventional barbell deadlift. As such, our primary objective was to compare peak force, the rate of force development (RFD), and muscle excitation with surface electromyography (EMG) during isometric deadlift pulls in resistance-trained participants with a conventional barbell versus a hexagonal barbell.

**Methods:** Ten healthy males (mean ± SD age = 26 ± 4 years, BMI = 25.1 ± 3.1 kg/m2) and ten healthy females (age = 21 ± 3 years, BMI = 22.8 ± 2.5 kg/m2) participated. Each participant performed three maximal, isometric deadlift pulls while standing on a force plate using a conventional barbell and a hexagonal barbell. The order in which each barbell was used was randomized. Bipolar surface EMG signals were recorded from the upper trapezius, external oblique, erector spinae, vastus lateralis, and biceps femoris. Peak force normalized to mass, RFD at 25, 50, and 75 ms intervals, and the amplitude (root-mean-square) of the EMG signals served as dependent variables.

**Results:** The difference in peak force when using the two barbells was trivial (p = 0.725, d = 0.080). However, there was a significant barbell x RFD interval interaction (F = 7.66, p = 0.006), with RFD75 being greater for the conventional barbell versus the hexagonal barbell (p = 0.041). Muscle excitation showed a significant barbell x muscle interaction (F = 9.64, p < 0.001). Vastus lateralis and upper trapezius excitation was higher for the hexagonal barbell compared to the conventional barbell. Of note, we observed no sex differences for any of our analyses.

**Conclusions:** In resistance trained adults, similar levels of peak force are produced with conventional and hexagonal barbells. However, rapid force production may be optimized with a conventional barbell. The use of a hexagonal barbell may be particularly useful for maximally activating the vastus lateralis and upper trapezius. Future clinical studies with larger samples are recommended to further evaluate differences between barbells.

**The Battle of the Sexes Part II: Does Prior Eccentric Exercise Affect Sleep?**

Veronica Mekhail^a^, Cassandra Evans^a,b^, Jason Curtis PhD^a,c^, Lia Jiannine PhD^a^, Paulina Czartoryski^a^, Jose Rojas^a^, Flavia Rusterholz^a^, Leonel Vargas^a^, Juan Carlos Santana^d^, Jose Antonio PhD^a^

^a^Exercise and Sport Science, NSU Florida, Davie, FL, USA^b^Health Sciences, Rocky Mountain University of Health Professions, Provo, UT, USA^c^Exercise and Sports Science, Keiser University Flagship, West Palm Beach, FL, USA^d^Institute of Human Performance, Boca Raton, FL, USA

Corresponding author: jose.antonio@nova.edu

**Background:** The purpose of this investigation was to determine if there are any sex differences in sleep after a bout of eccentric exercise designed to induce delayed-onset muscle soreness.

**Methods:** A total of 39 exercise-trained individuals, men (n = 20) and women (n = 19) (mean ± SD: age male 29 ± 9 yr, female 25 ± 8 yr; height male 179 ±6 cm, female 163 ± 8 cm; body mass male 89.7 ± 11.9 kg, female 61.3 ± 10.7 kg; lean body mass male 89.7 ± 11.9 kg, female 45.2 ± 7.2 kg; fat mass male 17.0 ± 8.4 kg, female 16.1 ± 7.0 kg; percent body fat male18.6 ± 7.5%, female 25.7 ± 7.9%; total body water liters male 53.3 ± 6.9 L, female 33.1 ± 5.2 L) participated in this trial. On visit 1, baseline characteristics were measured and participants completed a delayed-onset muscle soreness (DOMS) protocol. Specifically, participants completed 75 repetitions of elbow flexion of the non-dominant arm at 60% 1RM. Following completion of DOMS protocol, the following was assessed via self-report: sleep duration, time to fall asleep, number of awakenings, total time awake at night, sleep quality, and alertness upon awakening. Participants were assessed at baseline (pre-DOMS) and at 24- and 48-hours post.

**Results:** The mean duration of sleep for sleep was as follows: Baseline – male 7.3 ±1.4 hours and female 7.9 ±1.9 hours, respectively (p = 0.67); 24-hours post – male 7.6 ±1.0, female 8.0 ±1.6; 48-hours post (p >0.99) – male 7.6 ±1.0, female 8.1 ±1.2 (p = 0.49). Sleep quality as a determined via an 11-point scale (0 – worst, 10 – best) was as follows: Baseline – male 6.7 ±1.5, female 6.5 ±2.5 (p = 0.99), 24-hours post – male 6.3 ±2.1, female 6.2 ±2.5 (p = 0.99); 48-hours post – male 6.2 ±1.8, female 6.3 ±1.9 (p = 0.98).

**Conclusions:** There were no sex differences following eccentric loading that produced delayed-onset muscle soreness. It is not known if such a difference would exist with different types of exercise.

**Changes in Metabolic Rate, Vital signs and Mood Responses Following Ingestion of a Commercially Available Thermogenic Supplement**

Mandy E. Parra^a^, Christine M. Florez^a^, Jessica M. Prather^a^, Amie Vargas^a^, Bella Soto^a^, Abby Harrison^a^, Dylon Miller^a^, Matthias Tinnin^a^, Grant M. Tinsley^b^, & Lem W. Taylor^a^

^a^Human Performance Laboratory, School of Exercise and Sport Science, University of Mary Hardin-Baylor, Belton, TX, USA^b^Department of Kinesiology & Sport Management, Energy Balance and Body Composition Laboratory, Texas Tech University, Lubbock, TX, USA

Corresponding author: ltaylor@umhb.edu

**Background:** Variation in ingredients and dosages are common in thermogenic dietary supplements to elicit different proposed responses; however, research is lacking in these varied formulations on the market. Therefore, the purpose of this study was to determine the effects of a high stimulant version of thermogenic supplement on resting energy expenditure (REE), heart rate (HR), blood pressure (SBP and DBP), and mood states.

**Methods:** Forty-four individuals (22 F, 22 M; age: 21.5 ±3.4 y; height: 170.1 ±10.0 cm; body mass: 79.9 ±10.8 kg) participated in this placebo-controlled, double-blind, crossover design. Participants were randomized into one of two groups (placebo [PL] versus 1 serving of OxyShred Hardcore containing 275 mg caffeine, 1.5 g acetyl L-carnitine and 250 mg of glycine propionyl l-carnitine hydrochloride [TX]). Baseline values were collected for REE, HR, SBP and DBP, mood states and reassessed at 30-, 60-, and 120- minutes post-ingestion. Data were analyzed using repeated-measures analysis of variance. Follow up for significant effects was performed using pairwise comparisons with Tukey adjustment. Statistical significance was accepted at p <0.05.

**Results:** A significant condition X time interaction was observed for REE (p <0.0001). Despite lower REE at baseline in the TX condition ([mean±SE] −5.1 ±1.2%; p <0.05), REE was higher in TX than PL at 30 min (11.1 ±1.7%), 60 min (11.2 ±2.0%), and 120 min (8.4 ±1.2%) post-ingestion (p <0.0001). Statistically significant condition X time interactions were present for alertness (p <0.05), focus (p <0.01), and concentration (p <0.001). TX resulted in increased levels of alertness at all post-ingestion time points and increased focus and concentration at 30- and 60-min time points (p <0.05). For energy and fatigue, significant condition main effects indicated higher energy and lower fatigue in TX versus PL (p <0.001 to p <0.01). Significant time and condition main effects were observed for HR, with lower HR observed in the TX condition (p <0.01). Significant condition X time interactions were observed for SBP and DBP (p <0.01) indicating blood pressure was higher following TX ingestion when compared to PL at 30 min for DBP (p <0.001) and 60 min for SBP (p <0.05) although BP remained in normal clinical levels.

**Conclusions:** These data suggest that the thermogenic supplement investigated in this trial increased REE and this increase in metabolic rate was sustained for at least 120 minutes post-ingestion. Additionally, ingestion had positive effects on the mood states alertness, focus and concentration. Ingestion acutely increased blood pressure within normal limits that returned to non-significant levels and no negative effects on HR or side effects were observed.

**Acknowledgments:** This study was funded by EHP Labs. The authors declare no conflicts of interest.

**Effects of Acute and Short-Term Montmorency Tart Cherry Supplementation on Food-induced Increases in Uric Acid and Markers of Cardiometabolic Health: A proof of concept study**

Drew Gonzalez, Kay Nottingham, Megan Leonard, Broderick Dickerson, Tori Jenkins, Jacob Kendra, Choongsung Yoo, Dante Xing, Joungbo Ko, Ryan Sowinski, Christopher J. Rasmussen, and Richard B. Kreider

Exercise & Sport Nutrition Laboratory, Human Clinical Research Facility, Texas A&M University, College Station, TX, USA

Corresponding author: rbkreider@tamu.edu

**Background:** Elevated uric acid (UA) levels contribute to metabolic conditions including gout, hyperglycemia, inflammation, and obesity. Ingestion of food and/or beverages containing high purine levels increases UA levels and can complicate the management of markers of cardiometabolic health. Athletes who experience increases in UA levels may experience severe pain and require up to 10-days of rest to recover. Ingestion of polyphenols has been reported to significantly reduce the uricemic response to ingesting a meal high in purines. We previously reported that tart cherry powder (CherryPURE®), another naturally occurring source of polyphenols, lessened exercise-induced markers of inflammation. This study examined if this source of tart cherry powder can reduce the uricemic response to ingesting a high purine-containing meal and/or markers of cardiometabolic health.

**Methods:** In a double-blind, placebo-controlled, crossover, and counterbalanced manner, 25 adults (10 women and 15 men, 40.6 ±9 years, 85.0 ±17 kg, 29.1 ±4.9 kg/m2) with mildly elevated uric acid (UA) levels UA of 5.8 ±1.3 mg/dL) participated in the study. Participants were asked to restrict ingestion of purine-containing foods for 4-days prior to testing and replicated dietary intake before each session. Participants reported to the lab in a fasted state and donated a serum and whole blood sample. Participants were then randomly assigned to consume capsules containing 960 mg of a placebo (PLA) or Tart Cherry Powder (TC, CherryPURE®, Shoreline Fruit LLC, Traverse, MI) prior to consuming one serving of dried soup (10 g of carbohydrate, 2 g protein, 1 g fat) mixed in hot water that contains purines (i.e. 1 g of disodium 5’-guanylate, 1 g of adenosine 5’-monophosphate, and 1 g of disodium 5’-inosinate). Blood samples were then obtained after 60, 120, 180, and 240-min. Participants ingested two capsules per day of the assigned supplement for 7-days and returned to the lab to repeat the experiment. Participants observed a 10–14-day washout period, replicated their diet, and reported to the lab to repeat the experiment while administered the remaining supplement. Data were analyzed by General Linear Model (GLM) analyses with repeated measures using body weight as a covariate and are presented as mean changes from baseline with 95% confidence intervals.

**Results:** No statistically significant treatment x time interaction effects were observed in UA levels on Day 0, Day 7, or combined. However, UA values were generally lower at each data point after TC ingestion, particularly after 240-min. Ingestion of TC resulted in a −4.5% and −3.6% lower change in UA area under the curve (AUC) and a −5.0% and −5.4% lower change in total area under the first moment curve (AUMC) values after Day 0 and Day 7, respectively. Additionally, Day 0 blood glucose levels decreased from baseline to 240-min after ingestion with TC (−3.6 mg/dL [−0.1, 7.3], p = 0.057, $\eta_{p}^{2}$ = 0.07) while being unchanged with PLA. These findings provide some proof-of-concept support that ingestion of TC may affect the metabolic response to ingesting a high purine meal with a small amount of carbohydrate.

**Conclusions:** Acute and 7-days of TC supplementation (960 mg) did not promote statistically significant reductions in UA in response to a high purine meal. However, TC reduced the increase in UA levels by about 5% after acute (Day 0) and 7-days of supplementation. Additionally, TC ingestion decreased the glycemic response to the high purine meal on Day 0. Additional research should evaluate the timing of TC ingestion prior to a meal (e.g. 1–2 hours before a meal), pharmacokinetic effects over 8-hours, and whether a longer period of supplementation (e.g. 8–12 weeks) may promote greater effects on cardiometabolic markers.

**Figure:**
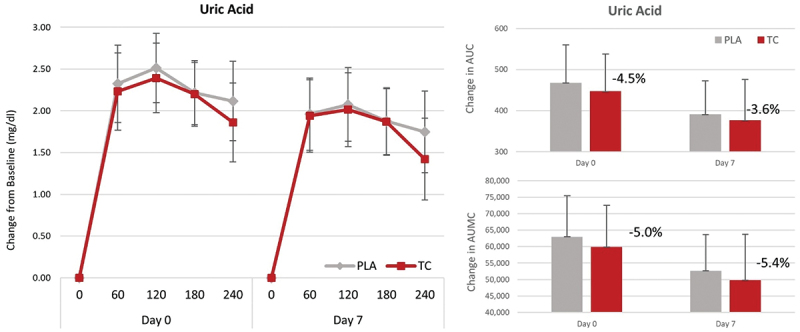

**Acknowledgments:** This study was funded by Anderson Advanced Ingredients (Irvine, CA) in collaboration with Shoreline Fruit LLC (Traverse, MI) as a fee-for-service project to the Human Clinical Research Facility at Texas A&M University and conducted by the Exercise & Sport Nutrition Lab.

**Rapidly Digestible Carbohydrate and Protein Drink May Be Needed to Optimize Heart Rate Recovery from Intense Exercise in Master Class Athletes**

Erica R. Goldstein^a^, Jeffrey R. Stout^a^, Ecaterina Vasenina^a^, David H. Fukuda^a^

^a^Physiology of Work and Exercise Response (POWER) Laboratory, Institute of Exercise Physiology and Rehabilitation Science, University of Central Florida, Orlando, FL, USA

Corresponding author: Erica.Goldstein@ucf.edu

**Background:** Heart rate recovery (HRR) is a practical and validated method for assessing cardiac autonomic recovery post-exercise. Fluid consumption within 60-min post-exercise has been shown to promote greater recovery of the autonomic nervous system compared to no fluid intake. This study examined HRR using a novel index considering total work to 3 different beverages at 1-, 2-, and 5-min post-exercise.

**Methods:** 24 male master class endurance athletes (age 49.3 ±6.6 years; height 175.8 ±4.6 cm; body mass 80.5 ±8.9 kg; body fat (%) 18.8 ±5.6; VO2peak 48.3 ±6.7 ml•kg•min−1) visited the laboratory on three separate occasions. During Visit #1, participants completed graded exercise testing (VO2peak; cycle ergometer). Familiarization (Visit #2) consisted of 5 × 4 min intervals at 70-80% of peak power output [PPO, watts] with 2 min of active recovery at 50 W, followed by a time to exhaustion test [TTE] at 90% PPO. The same high-intensity interval protocol with TTE was conducted pre-and post-beverage consumption on Visit #3. Participants were randomly assigned to receive one of three beverages during a 2-hour recovery period: PLA (electrolytes and water); CHO (1.2 g/kg bm); CHO-P coingestion (0.8 g/kg bm CHO + 0.4 g/kg bm protein). Heart rate was continuously recorded every 3 seconds. The HRR index (HRRi) was calculated as the last 3-sec peak heart rate value (beats per min; bpm) post-exercise plus the heart rate (bpm) recorded 1-, 2-, and 5-min post-exercise, divided by total work during the TTE. Differences in the HRRi between conditions were assessed via Quade’s nonparametric ANCOVA with LSD Post-Hoc comparisons if significant (p <0.05).

**Results:** There were significant differences among treatment groups at 1-min (p =.007), 2-min (p =.013), and 5-min (p =.008). Post-hoc comparisons indicated the HRRi was significantly different between PLA vs. CHO and CHO-P at 1-min (p =.003, p =.026), 2-min (p =.005, p =.029), and 5-min (p =.003, p =.025) respectively. There were no differences in posttest HRRi values between the CHO and CHO-P groups at any recovery time point.

**Conclusions:** Water plus electrolyte solution was inadequate for promoting HRRi compared to the CHO and CHO-P groups from the exhaustive exercise protocol used in this study. CHO and CHO-P appear equally effective in promoting HRR relative to total work in this group of male masters class endurance athletes. The results suggest that caloric intake in the form of rapidly digestible CHO or CHO-P during a 2-hour recovery period may be necessary to maximize HRR post exhaustive exercise.

**Readiness and its Relationship to GPS Metrics in Collegiate Soccer Players**

Maxine Furtado Mesa^a^, Michael J. Redd^a^, David H. Fukuda^a^, Jeffrey R. Stout^a^

^a^University of Central Florida, Orlando, FL, USA

Corresponding author: maxine.furtado@ucf.edu

**Background:** The purpose of this study was to investigate the relationship of readiness to performance efficiency index (effindex), distance covered per minute (d/min), distance covered at 14.99-18.99 kph (HSD), distance covered over 19 kph (Sprint), and training impulse (TRIMP) in collegiate soccer players. For the past ~25 years, coaches have used questionnaires to assess athletes’ readiness to compete and how well they are recovering. Coaches create periodized training plans to replicate different levels of match characteristics with varying levels of training load. Athlete responses to readiness questionnaires are sensitive to training load in collegiate soccer players and thus may be related to soccer performance.

**Methods:** Athletic performance and monitoring data from 20 male and 20 female collegiate soccer players during one competitive season were analyzed. Participants each wore Polar Team Pro sensors (PTPS) to record performance data (d/min, effindex, HSD, Sprint, and TRIMP) and be extracted from PTPS’s database for analysis. Participants filled out a readiness questionnaire prior to team-based activities on FitFor90’s website, fitfor90.com, to be extracted from the portal for analysis. The questionnaire consisted of six-items on a Likert scale from −3(worst) to +3(best) about current levels of fatigue, mood, stress, soreness, and the prior night’s sleep quality and quantity. Goalkeepers, athletes that experienced a season-ending injury, and/or were noncompliant with GPS wear and/or inconsistent with readiness questionnaire responses were excluded from analysis. Data was averaged weekly for analysis. Pearson product-moment correlations were used to describe relationships between readiness and performance data. Independent samples t-tests were used to compare differences in average readiness and performance data between male and female collegiate soccer players. A statistical significance level of p < .05 was set a prior for all analyses.

**Results:** Small, but significant correlations were observed between readiness and d/min (r = −0.195, p = .001; r = −0.240, p < .001), effindex (r = −0.191, p = .001; r = −0.185, p = .002) in male and female collegiate soccer players, respectively. Small, but significant correlations were observed between readiness and HSD (r = −0.165, p = .007), and readiness and Sprint (r = −0.277, p < .001) in female collegiate soccer players.

**Conclusions:** The results of this study indicate that readiness has a small inverse relationship with performance variables examined in collegiate soccer players during one competitive season. The inverse relationship may have been due to the daily undulating periodization that both the male and female soccer players underwent.

**References:**

1. Fields, JB, Lameira, DM, Short, JL, Merrigan, JM, Gallo, S, White, JB, et al. Relationship Between External Load and Self-Reported Wellness Measures Across a Men’s Collegiate Soccer Preseason. J Strength Cond Res 35, 2021.
2. Rabbani, A, Baseri, MK, Reisi, J, Clemente, FM, and Kargarfard, M. Monitoring collegiate soccer players during a congested match schedule: Heart rate variability versus subjective wellness measures. Physiol Behav 194: 527–531, 2018.

**Comparison of Hydration Status Determined by USG Compared to Plasma Osmolality in Division I Soccer Players**

Blaine S. Lints^a^, Bridget A. McFadden^a^, Emily A. Hu^a^, Harry P. Cintineo^a^, Alexa J. Chandler^a^, Gianna F. Mastrofini^a^, Shawn Arent, FISSN^a^

^a^University of South Carolina, Columbia, SC

Corresponding author: blints@e-mail.sc.edu

**Background:** Urine specific gravity (USG) is a commonly applied biomarker for rapid determination of hydration status. While USG benefits largely from convenience, it may not be as reflective of true hydration status when compared to plasma osmolality (Posm), the gold standard of hydration detection. Subsequently, decisions made regarding a team sport athlete’s readiness to play based on USG may warrant a certain degree of caution. Therefore, the purpose of this study was to compare the concordance between hydration status measured by USG and Posm in male and female Division 1 soccer players.

**Methods:** Sixty-four National Collegiate Athletic Association Division I soccer players (n = 31 women, n = 33 men; Mweight ± SE = 70.06 ± 0.90 kg; M%BF = 15.05 ± 0.94 %; MVO2max = 51.97 ± 0.76 mL/kg/min) participated in blood draws. Blood draws occurred in the morning and in a fasted state at six time points for the women and three time points for the men within throughout the season. Individual means of USG and Posm were calculated. Pearson-product moment correlations were used to assess relationships between individual means of USG and Posm. An alpha level of 0.05 was used to determine statistical significance.

**Results:** Mean Posm ± SE was 286.52 ± 0.47 mOsm/kg, while mean USG was 1.02 ± 0.00 for the women throughout the season. For the men, mean Posm was 289.39 ± 0.57 mOsm/kg, while mean USG was 1.02 ± 0.00. In both men (r = 0.05, p = 0.78) and women (r = −0.1326, p = 0.46), no statistically significant correlations were observed between individual means of USG and Posm.

**Conclusions:** These findings suggest a lack of agreement between USG and Posm when assessed over the course of a collegiate soccer season. While USG is capable of quickly providing a field-based estimate of hydration status, coaches and athletes should be mindful of potential differences when compared to Posm. In light of the fact that athletes routinely deal with significant variations in hydration status, the lack of correlation between USG and Posm suggest an athlete may be mistakenly categorized as hydrated or dehydrated prior to sport practice or competition.

**Pre-season Nutrient Intake and Energy Availability in NCAA Division III Collegiate Swimmers**

Dylan J. Klein^a^, Alaina Santacroce^a^, Victoria Montemorano^a^, Patrick McClain^a^

^a^Department of Health and Exercise Science, Rowan University, Glassboro, NJ, USA

Corresponding author: kleind@rowan.edu

**Background:** Poor dietary intakes in athletes can lead to low energy availability (LEA) and predispose for poor health and recovery in the face of vigorous training and sport. Low energy availability is defined as the difference between energy intake (EI, kcals/d) and exercise energy expenditure (EEE, kcals/d) normalized to fat-free mass (FFM, kg). A cutoff of < 30 kcals/kg FFM is currently used to indicate LEA. The competitive nature of collegiate athletics places a great demand on athletes to perform at a high level. This can potentially make it difficult for them to meet optimal EA and promote health and training adaptations. The purpose of this study was to assess the nutrient intakes, energy availability (EA) and prevalence of LEA in NCAA DIII swimmers at the start of pre-season.

**Methods:** This study was approved by the Rowan University Institutional Review Board. Fifteen male and 15 female swimmers completed dietary and body composition testing for this study. Energy intakes and relative nutrient intakes (g/kg/d) for carbohydrate, protein and fat were assessed using 3-day dietary records and analyzed using ESHA Food Processor. Exercise energy expenditures were assessed using 7-day exercise logs and the Compendium of Physical Activities. Body composition and FFM was assessed using bioelectric impedance analysis (BIA, InBody 770). Energy availability was calculated as the difference between EI and EEE, normalized to FFM (i.e. EA = (EI – EEE)/FFM).

**Results:** Energy intake was statistically significantly higher (p =0.007) in males than females during pre-season, whereas EEE was not statistically significantly different between the sexes (p =0.28). Energy availability was also not statistically significantly different between the sexes (p =0.65). Using a cutoff of <30 kcals/kg FFM, 43% (n =7 males, n =6 females) of swimmers exemplified LEA. Relative energy intakes of carbohydrate, protein and fat were not statistically significantly different between the sexes (p >0.05). However, 40% percent of males (n =6) and 20% of females (n =3) did not meet the sport recommended intake for carbohydrate (i.e. 3-10 g/kg/d), whereas 33% of both males (n =5) and females (n =5) did not meet the sport recommended intake for protein (i.e. 1.2-1.7 g/kg/d).

**Conclusions:** This study showed that EI, EEE, and EA in male and female NCAA DIII collegiate swimmers do not differ significantly at pre-season. Unfortunately, 43% of swimmers did exemplify LEA, which may contribute to poor health, training and recovery if not compensated for during the regular season. Moreover, sport recommended intakes for carbohydrate and protein were not sufficient in 20-40% of the athletes studied. These results indicate that NCAA DIII swimmers may require additional nutrition support during the regular season when barriers to optimal nutrition (e.g. increased training load, academics and travel schedules) are most burdensome relative to pre-season.

**The Effects of Pre-Sleep Feeding on Sleep Quality and Quantity in NCAA Division I Female Soccer Players**

Corrine J. Hickman^a^, Casey E. Greenwalt^a^, Liliana I. Rentería^a^, Katherine J. Schiltz^a^, Elisa Angeles^a^, Abbie Smith-Ryan^b^, Christopher Bach^c^, Matthew D. Vukovich^d^, Stacy Sims^e^, Tucker Zeleny^c^, Katie Holmes^f^, Ddavid Presby^f^, Michael J. Ormsbee, FISSN^a^

^a^Florida State University, Tallahassee, FL, USA^b^University of North Carolina, Chapel Hill, NC, USA^c^University of Nebraska-Lincoln, Lincoln, NE, USA^d^South Dakota State University, Brookings, SD, USA^e^Auckland University of Technology, Auckland, NZ^f^WHOOP, Inc., Boston, MA, USA

Corresponding author: Michael J. Ormsbee, PhD, FISSN

**Background:** Due to the high metabolic demands of sport, it is imperative that athletes appropriately support their training and recovery with adequate feeding and caloric consumption and sleep. Research indicates that pre-sleep nutrition can be used to optimize total daily protein intake and recovery, while also, acutely, improving muscle protein synthesis without influencing fat metabolism. However, little is known about the use of pre-sleep protein and the influence it has on sleep quality and quantity. The purpose of this study was to assess the effects of pre-sleep nutrition on sleep quality and quantity in elite female athletes.

**Methods:** Female soccer athletes from four Division I Universities wore WHOOP bands (WHOOP, Inc) 24 hours per day for the entire 2020-2021 competitive season to measure sleep quantity and quality. Surveys were deployed by the WHOOP App to the athletes every 3 days to collect data on pre-sleep feeding habits. The relationship between pre-sleep food consumption and sleep quantity, sleep quality, was examined. Data were de-identified and analyzed retrospectively using R Studio to facilitate the compilation of descriptive results.

**Results:** 83 female soccer players were enrolled, of which, 14 athletes provided two or more food logs and were included in the final analysis (mean ± sd: age 20.8 ± 1.4 years; height 167.9 ± 4.9 cm; weight 64.1 ± 6.9 kg; BMI 22.7 ± 0.5 kg/m2). The average pre-sleep nutritional intake was as follows: mean ± sd: kcals 330 ± 284; PRO 7.6 ± 7.3 g; FAT 12 ± 10.5 g; CHO 46.2 ± 40.5 g. Data were collected every third day during the 2020-21 season. There were no significant differences found between high (427.0 ± 220.0) vs low (144.4 ± 59.0) kcal intake on sleep duration (422.5 ± 34.9 minutes) or wake episodes (10.3 ± 0.8 episodes); high (15.6 ± 7.5 g) vs low (2.2 ± 1.3 g) PRO intake on sleep duration (432.2 ±8.9 minutes) or wake episodes (10.9 ± 0.5 episodes); high (18.8 ± 7.6 g) vs low (5.2 ± 2.6 g) fat intake on sleep duration (423 ±29.4 minutes) or wake episodes (10.8 ± 0.3 episodes); and high (66.3 ± 30.9 g) vs low (22.7 ± 9.2 g) CHO intake on sleep duration (460.1 ± 30.4 minutes) or wake episodes (10.8 ± 0.3 episodes).

**Conclusions:** Our data suggest that pre-sleep feeding in Division I female athletes does not impact sleep quantity or quality. However, the meals consumed were not calorically dense and may not have been significant enough to warrant a positive or negative response on sleep metrics. Furthermore, additional research is necessary to examine the effects of pre-sleep feeding on athletes’ sleep in a controlled setting.

**Acknowledgments:** This study was funded by WHOOP, Inc.

**Fatigue Increases Knee Joint Laxity Without Increasing ACL Size in Recreationally Active Individuals**

Katie N. Harris^a^, Ahalee C. Farrow^a^, Nigel C. Jiwan^a^, John R. Harry^a^

^a^Texas Tech University, Lubbock, TX, USA

Corresponding author: katrihar@ttu.edu

**Background:** The primary purpose of the study was to determine the effect of fatigue on knee joint laxity (KJL) and anterior cruciate ligament (ACL) size as well as to determine the relationship between KJL, ACL size, and body composition. Greater KJL and smaller ACL’s have both been identified as ACL injury risk factors. KJL is defined and measured as the passive movement of the tibia relative to the femur when a load is applied. Given the severity and prevalence of ACL injuries amongst athletes, this study was designed to gain a better understanding of the factors related to ACL size and KJL pre and post fatigue.

**Methods:** Ten recreationally active males (n = 5) and females (n = 5) (26.40 ± 5.32 years) completed a fatiguing drop jump protocol where continuous drop jumps were performed every 20 seconds until the participants could no longer achieve 80% of the averaged jump height for their initial five jumps. The height of the drop jump was individually set to 90% of the participant’s maximal countermovement jump height. ACL size and KJL of the right limb was measured pre and post fatigue. Ultrasound was used to image the ACL and the diameter of the anteromedial bundle of the ACL was quantified using ImageJ. KJL was measured using the Rolimeter knee arthrometer. Fat mass, lean mass, and bone mineral density of the lower right limb was assessed using Dual-Energy X-Ray Absorptiometry (DXA).

**Results:** Participants completed an average of 159.80 ± 95.49 drop jumps. KJL significantly increased pre (2.35 ± 1.15 mm) to post (3.28 ± 0.90 mm) fatigue, p < 0.01. In contrast, ACL size did not significantly change pre (6.72 ± 0.78 mm) to post (7.04 ± 0.86 mm) fatigue, p > 0.05. ACL size was not correlated to lean mass of the lower right limb, height, and weight, p > 0.05. Additionally, ACL size was not correlated to KJL, r(9) = 0.59, p = 0.07.

**Conclusions:** Fatigue increases KJL without changing ACL size. Since greater KJL is associated with ACL injuries, fatigue may increase the risk of sustaining an ACL injury. Understanding the factors related to ACL injury risks is the first step needed to identify at-risk individuals and provides additional knowledge to help develop methods to reduce the risk of injury. Further research is needed to understand the factors related to KJL and ACL size.

**Eating and Feeding Disorder Risk in Collegiate Endurance and Aesthetic Lean Sport Athletes**

Mackinsey K. Shahan^a^, Jeannine C. Lawrence^a^

^a^University of Alabama, Tuscaloosa, AL, USA

Corresponding author: mkshahan@crimson.ua.edu

**Background:** Lean sport athletes, including endurance (e.g. swimming and distance running) and aesthetic (e.g. gymnasts, cheerleading, and figure skating) athletes, place a high value on appearance and weight status to attain a competitive advantage. Among athletes, adequate dietary intake is essential to ensure optimal performance and reduce injury risk. However, disordered eating behaviors (DEBs) and eating disorders (EDs) are common. Additionally, these athletes may be at risk of a feeding disorder (FD). The purpose of this study was to assess the risk of EDs and FDs in a sample of collegiate lean sport athletes and compare DEBs and ED/FD risk in endurance and aesthetic-type athletes.

**Methods:** Participants were recruited from women’s lean sport teams in the United States. Females aged 18-30 years who were members of a college lean sport team were eligible. The Eating Attitudes Test (EAT-26) assessed DEBs and ED risk. FD risk was assessed using diagnostic questions from the Pica, ARFID, and Rumination Disorder Interview-ARFID self-report questionnaire (PARDI-AR-Q). Two additional questions assessed pica and rumination disorder risk based on FD definitions. Demographic data was reported as frequencies and/or mean values (± SD) as appropriate. Between-group comparisons were conducted using t-tests and Mann-Whitney U tests for data that was not normally distributed. Significance was set at <0.05.

**Results:** 211 participants were surveyed, mean age 19.7 (±1.3) years and mean BMI 23.2 (±3.8). 84.8% were white, 4.3% Asian, 1.4% African American, and 9.4% other/multiracial, with 8.5% reporting being of Hispanic/Latin/Spanish ethnicity. Participants reported having 9.5 (±4.5) years in their sport, and most spent a minimum of 11 hours per week in training or conditioning. 71.6% of participants were at risk for either an ED or FD. Among endurance athletes (n =110), 46.4% were at risk for ED, 66.4% at risk for FD, and 72.7% were at risk for either. For aesthetic athletes (n =101), 55.4% were at risk for ED, 64.4% at risk for FD, 70.3% at risk for either. Common DEBs included excessive exercise (39.1% endurance, 43.6% aesthetic) and binge eating (19.1% endurance, 15.8% aesthetic). Aesthetic athletes scored significantly higher overall on the EAT-26 test than endurance athletes (p <0.05).

**Conclusions:** Nearly three-quarters of female collegiate lean sport athletes reported being at risk for ED and/or FDs. Coaches and health professionals should be aware of the increased risk for developing DEBs, EDs, and FDs in these athletes. Improved screening and treatment resources for these disordered behaviors is critical for this population.

**Examining the Influence of Exercise on Mood Following a Stressor**

Amanda Holtzman^a^, Claudia Mendez^a^, Jozeph Cruz^a^, Gargee Pandya^a^, Jose Antonio FISSN^a^, Jonathan B. Banks^a^

^a^Nova Southeastern University, Fort Lauderdale, FL, USA

Corresponding author: Jonathan.banks@nova.edu

**Background:** Psychological stress results in a variety of negative consequences to psychological and physical health. Both aerobic exercise and mindfulness meditation have been shown to improve cognitive processes or facilitate emotional recovery from stressful exposure. This study aimed to examine the immediate ability of a brief acute bout of exercise or a mindfulness meditation induction to reduce the impact of a stressor on mood and identify the process through which these protective effects may function (improving attention or reducing mind wandering).

**Methods:** Using a repeated measures design, participants (N = 42, Female = 37, Mage = 18.34, SD = .94) completed one session per week in which they completed one of three conditions in a pseudorandomized order. In each session, participants either ran on a treadmill for 20 minutes (maintaining a perceived level of exertion between 12-14 on the Borg Scale), completed a mindfulness meditation for 15 minutes, or listened to a body relaxation meditation for 15 minutes (control). Following their intervention, participants completed a stress manipulation. Attention, mind wandering, and mood were measured before and after the intervention and stress manipulation.

**Results:** In the exercise condition, participants showed significantly less changes in mood following the intervention and the writing stressor. No significant effects of condition were observed for attention or mind wandering. Interestingly, mindfulness did not appear to significantly alter the impact of the intervention or stressor on mood change.

**Conclusions:** Findings suggest that a brief acute bout of exercise may be able to blunt the emotional impact of a subsequent stressor. It is possible that mindfulness training requires longer periods of engagement to reach the level of protective effects that exercise appears to have. Exercise does not appear to reduce the impact of stress on mood via attention or mind wandering. However, it is possible that these protective effects are exerted at a neurophysiological level that should be explored in future research and has been suggested in previous studies that our findings provide a replication of (Tartar et al., 2018).

**Influence of a Commercially Available Thermogenic Dietary Supplement on Basal Metabolic Rate, Hemodynamics, and Mood Responses in Caffeine-habituated Young Females**

Christine M. Florez^a^, Jessica M. Prather^a^, Amie Vargas^a^, Bella Soto^a^, Abby Harrison^a^, Dylon Miller^a^, Matthias Tinnin^a^, Grant M. Tinsley^b^, & Lem W. Taylor^a^

^a^Human Performance Laboratory, School of Exercise and Sport Science, University of Mary Hardin-Baylor, Belton, TX, USA^b^Energy Balance and Body Composition Laboratory, Department of Kinesiology & Sport Management, Texas Tech University, Lubbock, TX, USA

Corresponding author: ltaylor@umhb.edu

**Background:** Thermogenic supplements are popular ergogenic aids utilized by the general population to increase fat mobilization and promote weight loss. Though recent literature supports the safety of these supplements, the purpose of this study was to analyze the acute effects of a double-serving of a commercially available thermogenic supplement on resting energy expenditure (REE), clinical safety measures, and mood response in caffeine-habituated recreationally active females.

**Methods:** Twenty-two adult females (age: 20.8 ± 2.1 y; height: 165.6 ± 7.3 cm; weight: 75.2 ± 8.3 kg) participated in this placebo-controlled, double-blind, crossover design with two testing sessions separated by a 1-week washout. Participants were randomized into one of two groups (placebo [PL] versus 2 servings of OxyShred supplement, containing a total of 300 mg of caffeine and 3 g acetyl L-carnitine [TX]). Baseline values were collected for REE, blood pressure (SBP and DBP), heart rate (HR), hunger/satiety, and mood states and were repeated at 30-, 60-, and 120- minutes post-ingestion. All variables were assessed with a repeated-measures analysis of variance (ANOVA) and follow up pairwise comparisons with Tukey adjustment. Statistical significance was accepted at p <0.05.

**Results:** A significant condition by time interaction was observed for REE (p <0.0001), with follow up testing indicating higher REE in the TX group as compared to the PL group at magnitudes ranging from 11 to 12.1% at the 30-, 60-, 120- minute post-ingestion timepoints (p <0.05). HR was greater in the PL group (p <0.01) as compared to the TX group. No statistically significant differences were observed for SBP while DBP values in the TX group increased from baseline (p <0.05) and were greater than PL at the 30-minute (p <0.05); though values remained within normal limits. Subjective data revealed statistically significant treatment by time interactions (p <0.05) indicating that ingestion of TX improved alertness, concentration, and focus when compared to PL. No other differences in mood states, hunger/satiety or side effects were observed between groups.

**Conclusions:** These data suggest that acute ingestion of a thermogenic supplement increases metabolic rate and this observed increase in REE is observed for at least 120 minutes post ingestion. Although slight increases DBP occurred in the TX group, values remained within normal limits, and no differences in SBP, HR or side effects were observed.

**Acknowledgments:** This study was funded by EHP Labs. The authors declare no conflicts of interest.

**The Effect of Acute Refeeding on Weight Loss Under Hypocaloric Conditions**

Callie M Boddy^a^, Greg E Popovich^a^, Kristy D Henson^b^

^a^School of Exercise Science & Athletic Training, West Virginia Wesleyan College, Buckhannon, WV, USA^b^School of Science & Technology, Fairmont State University, Fairmont, WV, USA

Corresponding author: boddy.cm.2018@wvwc.edu

**Background:** In the context of a weight-loss program, implementation of a cheat meal or refeeding stage has been promoted as a means of ensuring unimpeded weight loss progress. Immediately following a refeeding, the body responds with a transient uptick in body weight attributed to sodium/fluid retention, glycogen, and the mass of the food. The body may respond with a rapid shedding of the extra weight a few days following the meal, and the individual may experience a new low on their weight-loss journey. The purpose of this study was to describe the relationship between an individual’s most recent or best weight and the net weight loss following acute refeeding.

**Methods:** This retrospective study involved analysis of clinical records from a local weight loss clinic. The subjects (n =84) adhered to the same nationally franchised weight-loss program. For each client, we recorded the sex, age, diabetic status, start weight, number of refeedings, weight before refeeding, whether weight was stagnant before refeeding, peak gain after refeeding, the number of days between the refeeding and weighing in, post-refeeding weight, macros of the meal (if reported), best recorded weight, and total weight lost.

**Results:** The average age of the participants was 43.9, comprised of 69 females and 15 males. The average number of cheat meals consumed was 5.78 (SD 5.56). Seventy-four individuals indicated that they did not experience a weight plateau before having a cheat meal. The average weight loss was 29 lbs (SD 20.8), and the average percent body weight decrease was −8.37% (SD 12.6). The average weight loss after a cheat meal was 0.76 lbs (SD 2.13) but the 75th percentile weight loss was 2.0 lbs. When testing for significance, there was significance between the number of cheat meals and total weight loss (p = 6.7E-20).

**Conclusions:** There is a statistically significant correlation between the number of cheat meals and weight loss. An important caveat is that those participants consuming a higher total number of weekly cheat meals have also been in a caloric deficit for a longer period of time (i.e. a greater number of weeks). After a refeeding, subjects in this study lost between 0.76 – 2.0 lbs in the ensuing week, depending upon the individual.

**Absence of Circadian Variation during a 24-hour Period in Sprint Performance using a Non-Motorized Treadmill**

Justine M. Renziehausen^a^, Amy M. Bergquist^a^, Jeffrey R. Stout^a^, and David H. Fukuda^a^

^a^University of Central Florida

Corresponding author: Justine.Renziehausen@ucf.edu

**Background:** Time of day has been shown to impact performance during the Wingate anaerobic test using a cycle ergometer. However, cycling may not be sport specific for many athletes, and running-based tests may allow for a more generalizable assessment. Therefore, the purpose of this study is to examine the effects of time of day on performance during a 30-second maximal effort sprint on a non-motorized treadmill (30nmt) with additional consideration for individual chronotype and body temperature.

**Methods:** This study followed a randomized crossover design. Participants included 26 recreationally active males (n =12) and females (n =14) between the ages of 18-35 years old (21.5 ± 2.4 years; 169.8 ± 7.3 cm; 67.1 ± 13.1 kg). Participants were asked to report to the laboratory on five occasions. On visit 1, participants completed the Morningness-Eveningness Questionnaire (MEQ) and two familiarization trials of the 30nmt. On visit 2, participants completed two additional familiarization assessments. On visits 3, 4, and 5, participants were asked to report to the laboratory for testing at 9:00am, 2:00pm, and 7:00pm within the same 24-hour period, in a randomized order. On each visit, participants completed temperature and anthropometric assessments, followed by a 5-minute standardized warm-up, 1 trial of the 30nmt, and a 5-minute cool down. Repeated measures ANOVAs with and without MEQ score as a covariate were conducted to examine differences in peak and mean power, peak and mean velocity, distance, and temperature at each time of day.

**Results:** Only two participants with an evening-type MEQ score were enrolled in the study and were not included in the final analysis. The average MEQ score was 54.231 ± 6.665, with 9 morning-types and 17 intermediate-types represented in the final sample. There was no significant difference in peak (ANOVA: p =.798; ANCOVA: p =.125) or mean (ANOVA: p =.080; ANCOVA: p =.082) power, peak (ANOVA: p =.465; ANCOVA: p =.287) or mean (ANOVA: p =.169; ANCOVA: p =.138) velocity, or distance (ANOVA: p =.266; ANCOVA: p =.166) throughout the day. Significant differences were found in tympanic temperature (ANOVA: p <.001; ANCOVA: p =.031) with the lowest values at 9:00am.

**Conclusions:** Overall, there were no significant differences in 30nmt performance throughout the 24-hour period for the current sample of morning-type and intermediate-type individuals, although temperature was significantly lower in the morning indicating circadian variation from a physiological perspective. Future research should include a larger sample with an adequate representation of all chronotypes.

**The Influence of CYP1A2 Genotypes on the Ergogenic Effects of Caffeine in Resistance Trained Females**

Jessica M. Prather^a*^, Christine M. Florez^a^, Amie Vargas^a^, Bella Soto^a^, Audrey Ross^a^, Abby Harrison^a^, Darryn Willoughby^a^, Lem W. Taylor^a^, Sydney Kutter^a^

^a^Human Performance Laboratory, University of Mary Hardin-Baylor, Belton, TX, USA

Corresponding author: jprather@mail.umhb.edu

**Background:** Caffeine is a neurostimulator that provides an ergogenic effect by binding to adenosine receptors and allowing individuals to increase perceived effort and time to exhaustion. Caffeine is metabolized in the liver primarily by the cytochrome P450 1A2 (CYP1A2) enzyme. A single nucleotide polymorphism, rs762551, determines if someone is a slow (AC/CC genotype) or a fast metabolizer (AA genotype). The role that genetics plays in caffeine metabolism and subsequent effect on exercise is not well established; therefore, this experiment investigated the impact that CYP1A2 genotypes have on the ergogenic effects of caffeine in females.

**Methods:** In this double-blind, placebo-controlled, crossover study, 36 resistance-trained and caffeine-habituated females (AA, n =20, 21.6 ±2.2 y, 163.2 ±9.6 cm, 70.3 ±15.4 kg; AC/CC, n =16, 22.3 ±3.8 y, 165.6 ±5.3 cm, 68.7 ±14.3 kg) were screened, had one-repetition maximums (1RM) determined on bench (BP) and leg press (LP), and performed a series of familiarization tests on the Wingate cycle test. Acute resistance exercise performance (muscular strength, endurance, and power) was assessed under both conditions (placebo-PL and 6 mg/kg-bw anhydrous caffeine-CAF). Menstrual cycle phase was standardized and testing sessions were conducted in the late follicular (LF) phase (approximately days 6-14). At 30 minutes post-ingestion, participants performed a 5-minute warm-up and then assessed LP 1RM, LP repetitions to failure at 70% (LPRTF) and repeated the same protocol for BP 1RM and repetitions to failure (BPRTF). Anaerobic peak (PP) and mean power (MP) were assessed using via Wingate cycle test. CYP1A2 genotype was determined by 23andMe using saliva. Data were analyzed using a two-way (condition x genotype) ANOVA with repeated measures and significance was accepted a priori at p <0.05.

**Results:** A significant condition x genotype interaction was observed for LPRTF in the CAF condition (p =0.038). Furthermore, the AA group experienced a greater increase in LPRTF compared to the AC/CC group (p =0.027). No significant main effects for condition or genotype were observed relative to LP 1RM, BP 1RM, BPRTF or Wingate MP and PP (all p >0.05).

**Conclusions:** In conclusion, the CYP1A2 AA and AC/CC groups had a similar ergogenic response in the resistance exercises, aside from leg press RTF, after the ingestion of 6 mg/kg-bw CAF. Our findings are the first to study this in a female resistance trained population, thus further research is required to understand if CYP1A2 genotype impacts the interindividual variability of metabolism rates and exercise performance.

**Acknowledgments:** This study was funded by a university Graduate Faculty Research Grant awarded. The authors declare no conflicts of interest.

**The Relationship Between Self-Reported Commercial Coffee Intake and Glycation Markers in Middle-Aged Midwestern US Adults: Preliminary Findings**

Betscakos, M.^a^, Botzman, H.^a^, Ruan, S.^a^, Lowery, L.^b^, and Scanlon, K.^a^

^a^University of Mount Union, Alliance, OH, USA^b^Abbott Nutrition, Columbus, OH, USA

**Background:** The purpose of this observational study was to investigate the relationship between advanced glycation end products (AGE), glycated hemoglobin (HbA1c), and habitual coffee consumption in adults 45-64y. AGE are proteins and lipids that become altered after exposure to sugars and HbA1c is a known marker of glucose tolerance. AGE rise over one’s lifespan and are associated with cardiovascular disease risk. The rate of accumulation is influenced by habitual consumption of processed foods, lack of physical activity and smoking. Although commercial coffees vary greatly in composition based on bean type, brew method, and added sugars, Lee et al. (2016) suggested that prediabetics who consumed at least three cups of (black) coffee daily exhibited a lower rate of disease progression than non-habituated participants. Chlorogenic acid inherent to coffee may be a mechanism, leading to beneficially altered sugar absorption and metabolism. We hypothesized free-living volunteers would exhibit an inverse relationship between self-reported coffee intake and measured HbA1c and AGE levels.

**Methods:** To measure HbA1c, mixed capillary blood (5ul) was obtained and analyzed via a PT Diagnostics HbA1c analyzer (Indianapolis, IN). Next, participants (N =31) underwent AGE measurement via ultraviolet light emittance and tissue fluorescence of the forearm (AGE-Reader, Diagnoptics, Inc. Groningen, Netherlands). Finally, using visual aids, participants estimated how many ounces of coffee they regularly consume per day and reported if they had a diagnosed metabolic condition.

**Results:** Self-reported volume of daily coffee intake (fluid oz.) positively correlated with AGE fluorescence score (0-5scale) (r =0.49;p =0.005), accounting for 24.4% of the variance in tissue glycation (r2 =0.244). Chronological age (r =0.27;p =0.137) and HbA1c (r =0.25;p =0.174) did not correlate with AGE score. Daily coffee intake was not associated with HbA1c (r =−0.02;p =0.912).

**Conclusions:** Contrary to the hypothesis, middle-aged participants exhibited a positive relationship between coffee intake and AGE, with no correlation for HbA1c. Added sugars may have overwhelmed the hypoglycemic effects of chlorogenic acid. Although these low-N, limited data increase the risk of Type II error for relationships like age and other glycation makers, the sample size was large enough to show significance between self-reported commercial coffee intake and AGE. Future interventions should include methods providing larger amounts of data such as continuous glucose monitoring, and should experimentally control for the type of coffee, use of sweeteners, and/or specific intake of chlorogenic acid.

**Acknowledgments:** Abbott Nutrition

**Effects of Peripheral Oxygen Saturation, Heart Rate, Reading Comprehension, and Emotional State in Children with and Without use of Facial Coverings**

Abigail Stack^a^, Lauren Roncone^a^, Kelsey Scanlon^a^

^a^University of Mount Union, Alliance, OH, USA

Corresponding author: scanlof@mountunion.edu

**Background:** The Covid-19 pandemic has presented unique challenges to K-12 as schoolboards and parents have concerns in making the most informed decisions regarding the extended wear of facial coverings and how it pertains to the safety of children. Face masks can cause discomfort, general irritation, and potential anxiety and distraction of students from instructional material delivered in class. The purpose of this study was to examine the effects of wearing a facial covering and the impacts on reading comprehension, heart rate (HR), peripheral oxygen saturation (SPO2), and emotional state throughout 30 minutes of regularly scheduled academic content in students’ grades K-5.

**Methods:** To assess reading comprehension, a short story was distributed (0 minutes) and read aloud to students while technicians collected HR and SPO2 via a pulse oximeter and asked students to identify their emotional state utilizing the How Am I Feeling visual scale (Buron and Curtis, 2003). Fifteen minutes later, HR and SPO2 were reassessed. After instructional time concluded (30 minutes), students were given a 4-question quiz pertaining to the story that was distributed prior while HR and SPO2 were reassessed, and students rated their emotional state. The procedure was then repeated in the opposite condition (mask v no mask) the following week at the same time of day with the same group of students.

**Results:** All participants (N =76) were recruited from the same school and data were analyzed via a one-way analysis of variance with repeated measures (ANOVA). There was no statistical significance between reading comprehension, heart rate, emotional state, nor oxygen saturation between the two conditions at any time point (p˃0.05). Furthermore, when participants were subjectively asked to answer the question: ‘Does your mask distract you from learning?’ 21.8% of the children responded with ‘yes’, 69.1% responded ‘no’, and 9.1% said, ‘I don’t know’.

**Conclusions:** There were no apparent adverse biological or cognitive effects on elementary school children while wearing a cloth face covering in the classroom for 30 consecutive minutes of instructional time within the parameters of this study. As cultures continue to make their way out of the COVID-19 pandemic, parents, scientists, and educators can take comfort in data supporting that masks do not explicitly present an obvious threat to health nor learning when worn in the classroom.

**Acknowledgments:** None

**The Effects of Pyrroloquinoline Quinone Supplementation with Endurance Exercise on Acute Gene Expression of Mitochondrial Biogenesis and Function in Untrained Males**

Paul S. Hwang^a,b^, Steven B. Machek^b,c^, Thomas D. Cardaci^b,d^, Dylan T. Wilburn^b^, Emiliya S. Suezaki^b^ & Darryn S. Willoughby FISSN^e^

^a^Department of Kinesiology, Vanguard University, Costa Mesa, CA, USA^b^Exercise & Biochemical Nutrition Laboratory, Department of Health, Human Performance, & Recreation, Robbins College of Health and Human Sciences, Baylor University, Waco, TX, USA^c^Division of Natural Sciences and Mathematics, University of the Ozarks, Clarksville, AR, USA^d^Department of Pathology, Microbiology, and Immunology, University of South Carolina School of Medicine, Columbia, SC, USA^e^School of Exercise and Sport Science, Mayborn College of Health Sciences, University of Mary Hardin-Baylor, Belton, TX, USA

Corresponding author: dwilloughby@umhb.edu

**Background:** Pyrroloquinoline quinone (PQQ) is a novel supplement physiologically involved with mitochondrial function. Similarly, aerobic exercise elicits elevations in genetic expression for mitochondrial activity. Prior published data presented evidence that the combination of PQQ supplementation with endurance training increased skeletal muscle PGC-1α protein content in untrained males, suggesting adaptations for mitochondrial biogenesis. Currently, there is no data on the effects of PQQ supplementation with endurance exercise on genetic activity for mitochondrial function in humans. Therefore, the current study investigated the acute effects of PQQ supplementation with endurance exercise on the expression of genes related to mitochondrial function in young untrained males.

**Methods:** In a randomized, double-blind, placebo-controlled design, untrained [<3 hr/wk exercise for ≥1 year prior to starting the study] males aged between 18-35 (n =23) were randomly assigned to either a PQQ (n =12) or cellulose placebo (PLC; n =11) group. Participants underwent a percutaneous muscle biopsy from the vastus lateralis using the fine needle aspiration technique at baseline, 0.5-hour post-exercise and 2-hour post-exercise. Following the baseline muscle biopsy, participants ingested their respective supplement (20 mg) prior to a VO2peak test on a stationary bike. Real-time PCR (RT-PCR) was utilized to assess gene activity, which included PGC-1α, Citrate Synthase (CS), Cytochrome C-1 (CYC-1), and Cytochrome C Oxidase (COX 4/1). Factorial 2 × 3 Supplement [Group (PQQ, Placebo)] x Time [Baseline, 0.5-Hour Post-Exercise, 2-Hour Post-Exercise] mixed methods analyses of variance (ANOVA) was conducted for all criterion variables at a significance of p <0.05.

**Results:** There was no significant supplement by time interactions for all gene targets (p >0.05). However, there was a main time effect for PGC-1α and CS activity (p =0.002). Nevertheless, relative to gene expression, the magnitude difference may not be practically significant. Overall, an acute dose of PQQ with exercise presented no meaningful differences in gene activity for mitochondrial function.

**Conclusions:** An acute dose of PQQ combined with a bout of endurance exercise may not elicit a significant fold expression in targeted genes within untrained males. The limitations of solely 2 time-points for gene expression post-exercise may have minimized possibility in observing peak elevations. Furthermore, the methodological differences corresponding to our exercise bout (VO2Peak test), and no standardization of prior dietary status beyond self-reported dietary logs may explain the lack of gene activity. Further research is warranted to ascertain any effects following acute PQQ supplementation with variations in intensities, durations, or modalities of exercise for ergogenic potential on mitochondrial function in humans.

**Fight club – Between-Sex and Within-Sex Differences in Body Composition Variables**

Jackie Kaminski^a^, Corey Peacock^a^, Chris Algieri^a^, Jose Rojas^a^, Anthony Ricci^a^, Cassandra Evans^a^, Veronica Mekhail^a^, Jose Antonio^a^

^a^Department of Health and Human Performance, Fight Science Lab, Nova Southeastern University, Davie, FL, USA

Corresponding author: jose.antonio@nova.edu

**Background:** There is a dearth of data on professional fighters particularly in the mixed martial arts. Thus, the purpose of this investigation was to describe the body composition variables on male and female professional fighters.

**Methods:** A total of 28 professional fighters participated in this investigation (n =22 male, n =6 female). The majority (68%) of the fighters competed in the Ultimate Fighting Championship (UFC) (n =19). The remaining fighters competed in various other promotions (e.g. BKFC, Bellator, Eagle FC, Valor, etc.). Body composition was assessed via dual-energy x-ray absorptiometry (DXA). Total and regional body composition was determined.

**Results:** There were significant sex differences for height, body mass, lean body mass, bone mineral content, whole body bone mineral density, Z score, regional bone mineral density (except for the head), regional percent fat (except for the head), and percent body fat; however, no differences were found for age or whole-body fat mass. In males, there was a significant relationship between lean tissue mass and bone mineral density. Furthermore, there existed within-sex differences for male fighters (i.e. left vs. right leg and arm). There were significant differences between the left and right arm for lean mass; in addition, fat mass differed between the left and right leg in males.

**Conclusions:** It is evident that profound sex differences exist vis a vis body composition. It should be noted that the bone mineral density is exceedingly high in this group of athletes. Furthermore, there tends to be asymmetry in both fat mass and lean mass in male fighters.

**The Effect of Acute Caffeine Withdrawal on Exercise Performance in Habitual Caffeine Users**

Timothy D. Griest^a^, Michael J. Saunders^a^, Christopher J. Womack^a^, Haley L. McVannel^a^, Kylie M. Moulin^a^, Cassidy A. Finley^a^, Nicholas D. Luden^a^

^a^Human Performance Laboratory, James Madison University, Harrisonburg, VA, USA

Corresponding author: ludennd@jmu.edu

**Background:** Caffeine (CAF) is widely used to enhance cognitive and physical performance. Habitual CAF use leads to physical dependance such that the absence of CAF evokes withdrawal symptoms that typically peak 20-48 h following the most recent dose. The impact of CAF withdrawal on acute exercise performance is currently unclear. Therefore, the primary aim of this project was to quantify the effects of withdrawal on performance, not only to assess the impact of withdrawal, but also to discern whether CAF ingestion elicits a net beneficial effect in habitual CAF users, or simply relieves the negative impact of withdrawal.

**Methods:** Ten recreational cyclists (age 39.1 ± 14.9 y; VO2max 54.2 ± 6.2 mL/kg/min) who were habitual CAF users (394 ± 146 mg/d) completed four trials, each consisting of peak isokinetic torque testing and a 10-km time trial (TT). On each trial day, subjects consumed either 1.5 mg/kg CAF to prevent withdrawal or placebo (PLA) 8 h before their laboratory visit, and then 6 mg/kg CAF or PLA 1 h prior to exercise. In a randomized counterbalanced design, each subject completed the following treatments: PLA 8 h pre-exercise + PLA 1 h pre-exercise (PLAW), CAF + PLA (PLAN), PLA + CAF (CAFW), CAF + CAF (CAFN).

**Results:** CAF withdrawal did not impair TT performance (PLAW, 18.86 ± 2.68 min vs. PLAN, 18.28 ± 1.62 min, p = 0.17). However, pre-exercise CAF ingestion only improved TT performance when compared to the PLAW trial (CAFN, 18.23 ± 2.10 min vs. PLAW, p = 0.014, CAFW, 18.05 ± 1.99 min vs. PLAW, p = 0.01). When withdrawal was mitigated, pre-exercise CAF did not enhance TT performance to pre-exercise PLA (PLAN vs. CAFN p = 0.636). Peak isokinetic torque (30 deg/s) was elevated in the CAFN (155.6 ± 28.9 ft-lbs) condition versus the PLAW (145.9 ± 30.7 ft-lbs) condition (p = 0.011), with no other differences between conditions.

**Conclusions:** CAF withdrawal does not directly impair exercise performance, and pre-exercise CAF only improves performance when compared to bouts in which withdrawal is being experienced, suggesting that habitual CAF users may not benefit from acute CAF supplementation unless they are experiencing the effects of CAF withdrawal.

**What is the relationship between nutritional knowledge and the fatigue and rate of perceived exertion on NCAA Division III women’s soccer players during match play?**

Laura Mason^a^, Jason Cholewa^a^, Sean Collins^a^, Nicki Favero^a^

^a^University of Lynchburg, Lynchburg, VA, USA

Corresponding author: mason_ln@lynchburg.edu

**Background:** Soccer is a high-intensity intermittent and metabolically demanding sport which requires adequate nutritional supply. Player’s time to exhaustion is correlated with muscle glycogen levels, therefore, consumption of carbohydrates pre-exercise and post-exercise is critical in order to maximize performance and recovery. NCAA Division I athletes have reported inadequate nutritional knowledge and not meeting their energy consumption needs, putting them at a higher risk of injury and decreased performance. Sports nutrition has become more popular since there is an increased awareness of the impact on performance levels. While substantial research has been done among Division I athletes little known at the Division III level in athletes on their sports nutrition knowledge. The purpose of this correlational study was to determine if a relationship is present between nutritional knowledge and rate of perceived exertion and fatigue in NCAA Division III women’s soccer players.

**Methods:** Twenty-four subjects were recruited from the University of Lynchburg Women’s Soccer team. Subjects were aged 18-22, female, and active participants of the team. A sports Nutrition Knowledge Questionnaire developed by Reilly and Maughan was administered to all subjects to complete at the beginning of study. Prior to games, subjects completed the Hooper Index to determine fatigue levels. After games, subjects completed RPE scores. Playing time was calculated per subject to calculate session-RPE. Subjects were surveyed across 8 games during the season. The relationship between nutritional knowledge and the Hooper Index and s-RPE was analyzed with Pearson’s correlation and significance was set at p <0.05.

**Results:** The average score of the Sports Nutrition Questionnaire was 52.8 ±13.7 among all subjects. The average Hooper Index score was 14.6 ±2.1 among all subjects. The average s-RPE was 276.1 ±235.7 among all players. No significant correlation (p =0.533) was found between Sports Nutrition Questionnaire and Hooper Index (r =−0.134) in all subjects. No significant correlation (p =0.686) was found between Sports Nutrition Questionnaire and s-RPE (r =0.087) in all subjects. No significant correlation (p =0.499) was found between Hooper Index and play time (r =0.145) in all subjects. Median playing time was 27 minutes, and subjects that averaged greater than 27 minutes were defined as high volume players, and those less than 27 minutes low volume players. No significant correlation (p =0.508) was found between Sports Nutrition Questionnaire and Hooper Index (r =−0.145) in subjects who played the most during the season. No significant correlation (p =0.565) was found between Sports Nutrition Questionnaire and s-RPE (r =0.127) in subjects who played the most during the season.

**Conclusions:** In conclusion, NCAA Division III women’s soccer players have inadequate sports nutrition knowledge, but there were no relationships with rating of perceived exertion and levels of fatigue. However, it should be noted that players can benefit from increasing their sports nutrition knowledge by having access to a registered dietitian focused on sports nutrition. This will allow athletes to improve their dietary behaviors in order to properly prepare them for competition and proper recovery post-competition.

**Acknowledgments:** University of Lynchburg Women’s Soccer Team for their participation in my research. My research committee for their help throughout the study.

**Resistance Exercise Intensity is Related to Gastrointestinal Symptoms and Damage in Resistance-trained Adults**

Jeremy R. Townsend^a^, Tricia L. Hart^a,b^, Natalie J. Grady^a^, Kent D. Johnson^a^, Laurel A. Littlefield^a^, Matthew J. Vergne^c^, Gabrielle Fundaro^d^

^a^Exercise and Nutrition Science Graduate Program, Lipscomb University, Nashville, TN, USA^b^Department of Nutritional Sciences, Penn State University, University Park, PA, USA^c^Department of Pharmaceutical Sciences, Lipscomb University, Nashville, TN, USA^d^Vitamin PhD Nutrition LLC, Lawrenceville, GA, USA

Corresponding author: jrtownsend@libscomb.edu

**Background:** Resistance exercise (RE) can provoke significant elevations in gastrointestinal (GI) symptoms, damage, and permeability. It is believed that high levels of intra-abdominal pressure impacts GI distress with absolute RE intensity as a primary contributor to GI issues following exercise. The purpose of this study is to investigate the association between maximal strength, relative strength, GI symptoms, and indirect markers of GI damage and permeability in resistance-trained males and females.

**Methods:** Thirty resistance-trained men [n = 15, 24.2 ± 4.0 yr, 90.9 ± 22.3 kg, 143.2 ± 28.9 kg Squat 1-Repetition Maximum (1RM)] and women (n = 15, 23.6 ± 3.9 yr, 69.8 ± 16.6 kg, 87.7 ± 20.2 kg Squat 1RM) free of any underlying GI issue or pathology completed a RE bout and a non-exercise control (CON) session in a randomized, counterbalanced design. The RE protocol utilized a load of 70% 1RM for 4 sets of 10 repetitions with a 90-second rest period between sets and a 120-second rest period between exercises (squat, seated shoulder press, deadlift, bent-over row, leg press). Blood samples were collected before exercise (PRE), immediately- (IP), 15-, 30-, and 60-minutes (60 min) post-exercise. GI symptom questionnaires were completed by participants to assess subjective upper and lower GI symptoms at PRE, IP and 60 min post-exercise with a scale of 0 (no symptom) to 9 (worst it has ever been). Blood samples were assayed to quantify small intestine damage (I-FABP) and GI permeability [Lactulose/Rhamnose (L/R) ratio] with all measures corrected for plasma volume shifts. Relationships between Total Absolute and Relative 1RM Strength (all 1RM values for each exercise summed), Total GI symptoms, blood markers of damage and permeability (I-FABP and L/R ratio) were analyzed using Pearson product moment correlations.

**Results:** Total Absolute 1RM Strength was significantly correlated to I-FABP values at IP (r = 0.559; p = 0.001), 15 min (r = 0.503; p = 0.005), and 30 min (r = 0.417; p = 0.022) while having no association to L/R Ratio. Total Relative 1RM Strength was only associated with I-FABP at IP (r = 0.385; p = 0.036) and not correlated to L/R Ratio. There were moderate correlations between Total GI Symptoms and Total Absolute 1RM Strength (r = 0.350, p = 0.058) and Total Relative 1RM Strength (r = 0.359; p = 0.051) but these relationships did not reach significance.

**Conclusions:** It appears that RE intensity is related to GI symptoms and damage (I-FABP) following exercise and is not associated with GI permeability (L/R Ratio) in resistance-trained adults.

**A Multi-ingredient Supplement Enhances Mood and Attention**

Jessica Piedra^a^, Bianka Texidor^a^, Jason Curtis PhD^b^, Cassandra Evans^c^, Veronica Mekhail^a^, Paulina Czartoryski^a^, Kunal Vani^a^, Austin Marchetta^a^, Daniela Diaz^a^, Charles Thumm^a^, Juan Carlos Santana^d^, and Jose Antonio PhD FISSN^a^

^a^Exercise and Sport Science, Nova Southeastern University, Florida, Davie, FL, USA^b^Exercise and Sports Science, Keiser University Flagship, West Palm Beach, FL, USA^c^Health Sciences, Rocky Mountain University of Health Professions, Provo, UT, USA^d^Institute of Human Performance, Boca Raton, FL, USA

Corresponding author: jose.antonio@nova.edu

**Background:** There is evidence to suggest that multi-ingredient pre-workout supplements may exert a profound effect on both mental and physical performance. Thus, the purpose of this study was to investigate the effects of a multi-ingredient pre-workout supplement (MIPS) on mental and physical performance.

**Methods:** A total of 14 exercise-trained individuals, men (n = 7) and women (n =7) (mean ± SD: age 19.9 ± 1.4 yr; height 168.2 ± 11.3 cm; body mass 68.9 ± 10.7 kg; lean body mass 54.9 ± 12.5 kg; fat mass 14.0 ± 5.5 kg; percent body fat 21.0 ± 9.0%; total body water liters 40.2 ± 9.2 L; total number of years training 6.3 ± 3.5) average hours of aerobic exercise per week 3.6 ± 3.5; average hours of resistance exercise per week 7.4 ± 4.9; other exercise per week 1.8 ± 2.8; average caffeine consumed per day 207 ± 112) completed this randomized, placebo-controlled, double-blind, counterbalanced, crossover trial. Participants consumed either a multi-ingredient pre-workout supplement (MIPS) or a placebo in a randomized, counterbalanced order. Forty-five minutes post-consumption, the following assessments were conducted: psychomotor vigilance (PVT), Profile of Mood States (POMS), vertical jump test, and heart rate (HR) and blood pressure (BP). There was a one-week washout period between assessments.

**Results:** There were statistically significant differences between the treatment and placebo for the PVT (reaction time: treatment 286 ± 28 ms, placebo 306 ± 46 ms, p = 0.0371, and POMS (i.e. vigor: treatment 15.2 ± 14.9, placebo 9.7 ± 9.6, p = 0.0403; fatigue: treatment 1.0 ± 1.1, placebo 3.3 ± 3.4, p = 0.0100). There were no significant differences between groups for the other indices of mood, false starts from the PVT, and vertical jump.

**Conclusions:** The acute consumption of a multi-ingredient pre-workout supplement produced a significant improvement in vigilance as well as measures of vigor and fatigue.

**Acknowledgments:** This study was sponsored by a grant from SHIFTED (Eugene, Oregon USA) to the ISSN.

**The Effects of a Multi-ingredient Supplement on Various Cognitive Measures**

Daniela Diaz^a^, Austin Marchetta^a^, Jason Curtis PhD^b^, Jessica Piedra^a^, Bianka Texidor^a^, Cassandra Evans^c^, Veronica Mekhail^a^, Paulina Czartoryski^a^, Kunal Vani^a^, Charles Thumm^a^, Juan Carlos Santana^d^, and Jose Antonio PhD FISSN^a^

^a^Exercise and Sport Science, Nova Southeastern University, Florida, Davie, FL, USA^b^Exercise and Sports Science, Keiser University Flagship, West Palm Beach, FL, USA^c^Health Sciences, Rocky Mountain University of Health Professions, Provo, UT, USA^d^Institute of Human Performance, Boca Raton, FL, USA

Corresponding author: jose.antonio@nova.edu

**Background:** Multi-ingredient pre-workout supplements (MIPS) have been shown to affect exercise performance. Endurance-trained runners have increased time to fatigue during sustained running at lactate threshold. Caffeine energy drink also showed improvements in performance during a 1-hour timed cycling trial. An investigation on an energy drink containing caffeine rather found that those who consumed the energy drink had less false starts during a Psychomotor Vigilance Test (PVT) versus the placebo. Thus, the purpose of this investigation was to assess whether a caffeine-containing MIPS affected various physical and neurophysiologic measures.

**Methods:** Seventeen exercise-trained men (n = 7) and women (n =10) completed this randomized, placebo-controlled, double-blind, counterbalanced, crossover trial. Participants consumed either a multi-ingredient pre-workout supplement (MIPS) or a placebo in a randomized, counterbalanced order. Forty-five minutes post-consumption and at 90 minutes post-consumption, the following assessments were conducted: psychomotor vigilance test (PVT), Profile of Mood States (POMS), Stroop test, vertical jump test, and a cold pressor test (CPT). There was a one-week washout period between assessments.

**Results:** There were significant differences in the incongruent task for the Stroop test where the treatment group was faster than the placebo group at the 45-minute post-consumption (reaction time: treatment 862 ± 160 ms, placebo 942 ± 204 ms, p = 0.0424). There were no significant differences in the Stroop test in congruent scores at either time interval or the incongruent at the 90-minute interval. There was not a significant difference between groups in the psychomotor vigilance (PVT) at either interval. There are no significant differences in either interval for the CPT, the vertical jump, or the Profile of Mood States (POMS) as well.

**Conclusions:** A multi-ingredient pre-workout supplement (MIPS) resulted in better performance on the incongruent task vs. the placebo group 45 minutes post-consumption.

**Acknowledgments:** Product and placebo were an in-kind donation from MRM (Oceanside CA USA).

**The Battle of the Sexes: Women Can Take More Pain than Men?**

Cassandra Evans^a,c^, Jason Curtis PhD^b,c^, Veronica Mekhail^c^, Lia Jiannine PhD^c^, Paulina Czartoryski^c^, Jose Rojas^c^, Flavia Rusterholz^c^, Leonel Vargas^c^, Juan Carlos Santana^d^, Jose Antonio PhD^c^

^a^Health Sciences, Rocky Mountain University of Health Professions, Provo, UT, USA^b^Exercise and Sports Science, Keiser University Flagship, West Palm Beach, FL, USA^c^Exercise and Sport Science, Nova Southeastern University, Florida, Davie, FL, USA^d^Institute of Human Performance, Boca Raton, FL, USA

Corresponding author: jose.antonio@nova.edu

**Background:** Sex differences in pain tolerance and perceived pain exists. Similar evidence suggests that the effects of exercise can vary between genders. The purpose of this investigation was to assess sex differences in skeletal muscle pain/soreness following a bout of eccentric loading of the non-dominant arm.

**Methods:** A total of 39 exercise-trained individuals, men (n = 20) and women (n =19) (mean ± SD: age male 29 ± 9 yr, female 25 ± 8 yr; height male 179 ±6 cm, female 163 ± 8 cm; body mass male 89.7 ± 11.9 kg, female 61.3 ± 10.7 kg; lean body mass male 89.7 ± 11.9 kg, female 45.2 ± 7.2 kg; fat mass male 17.0 ± 8.4 kg, female 16.1 ± 7.0 kg; percent body fat male18.6 ± 7.5%, female 25.7 ± 7.9%; total body water liters male 53.3 ± 6.9 L, female 33.1 ± 5.2 L) participated in this trial. On visit 1, baseline characteristics were measured and participants completed a delayed-onset muscle soreness (DOMS) protocol. Specifically, participants completed 75 repetitions of elbow flexion of the non-dominant arm at 60% 1RM.Following completion of DOMS protocol the following assessments were performed: self-reported feelings of soreness, soreness via algometer and muscle circumference. Participants underwent the same assessments at 24-hr, 48-hr and 72-hrs.

**Results:** Significant sex differences were reported in peak perceived soreness (48-h peak perceived soreness: males 5.1 ±4.0, females 0.8 ±3.7, p =0.0113; 72-h peak perceived soreness: males 4.4 ±4.5, −0.4 ±3.9, p =0.0033). There were no significant differences between the males and females in pain as assessed via pressure threshold (24-hrs, 48-hrs and 72-hrs) and muscle circumference (24-hrs and 48-hrs).

**Conclusions:** Women perceive less muscle pain than men following a bout of eccentric exercise of the non-dominant elbow flexor muscles.

**Blood Lactate Responses to a Stand-up (SUP) Paddling Race: a Pilot Trial**

Joseph Petruzzulli^a^, Amani Khan^a^, Cassandra Evans^a^, Veronica Mekhail^a^, Paulina Czartoryski^a^, Flavia Rusterholz^a^, Jose Antonio PhD^a^, Victoria Burgess PhD^b^

^a^Exercise and Sport Science, Nova Southeastern University, Florida, Davie, Florida, USA^b^Concordia University, Chicago, IL, USA

Corresponding author: Jose.Antonio@nova.edu

**Background:** The blood lactate response to exercise is indicative of the contribution of the glycolytic energy system (aka lactic acid energy system). As exercise intensity increases, the reliance on glycolysis also increases thus resulting in higher concentrations of blood lactate. To date, there are no studies on the blood lactate response to a stand-up paddling race. Thus, the purpose of this pilot trial was to measure blood lactate at rest and after a stand-up paddling (i.e. SUP) race in a group of well-trained paddlers.

**Methods:** A total of 8 exercise-trained stand-up paddlers, men (n = 4) and women (n =4) (mean ± SD: age 46 ± 12 yr; height 66 ± 4 in; body mass 151 ± 19 lb; BMI 24 ± 2; total number of years training 23 ± 18; total number of years paddling 5 ± 4) completed this pilot trial. Measurements of blood lactate were taken pre- and post-race (5-kilometer distance) from the surface of the subjects’ index finger (Lactate Plus Meter Nova Biomedical, Waltham MA).

**Results:** There were statistically significant differences between the pre-race lactate responses and post-race lactate responses (pre-race lactate 2.2 ± 1.5 mmol/L; post-race lactate 5.3 ± 1.8 mmol/L, p = 0.0005).

**Figure:**
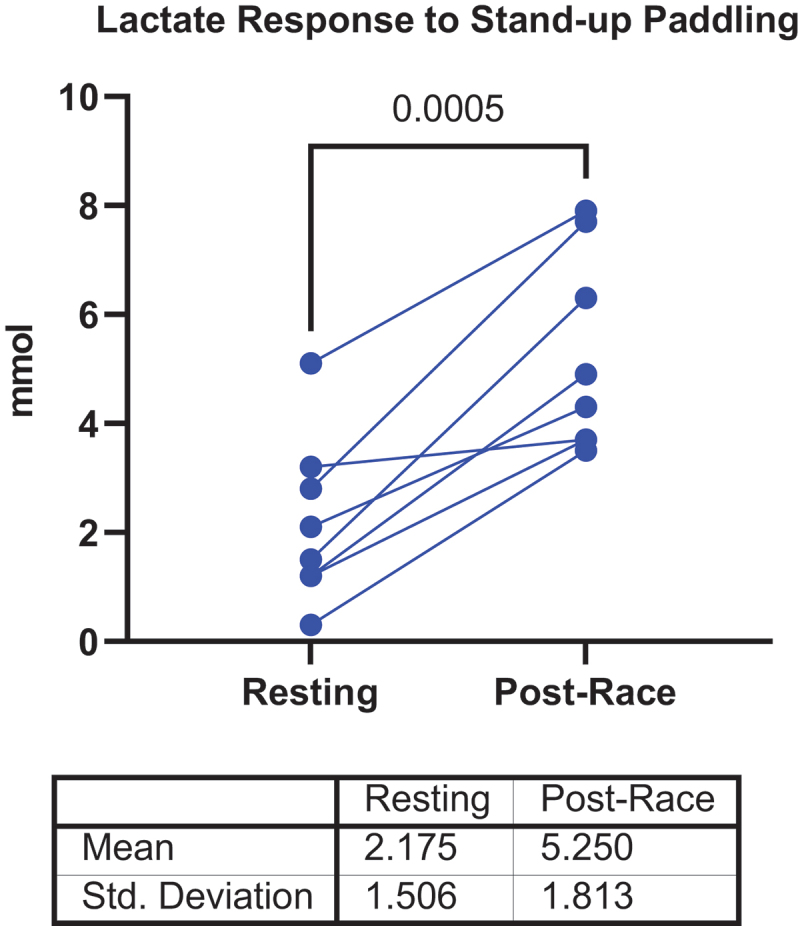

**Conclusions:** Stand-up paddling induces a significant elevation of blood lactate thus indicating a significant contribution of the lactic acid energy system (i.e. fast glycolysis) to this form of exercise.

**The Effect of Melatonin on Insomnia: Age Matters**

Minh K. Chau^a^, Kayla Thompson^a^, Karelys Montanez^a^, Spencer Hohm1, Nita Pedavalli^a^, Rossanna Villaverde^a^, Ana Fins PhD^a^, Jaime Tartar PhD FISSN^a^

^a^Psychology and Neuroscience, Nova Southeastern University, Davie, FL, USA

Corresponding author: mc4076@mynsu.nova.edu

**Background:** It has been posited that sleep or lack thereof has a tremendous impact on health and exercise performance. Individuals with sub-threshold insomnia struggle with falling and staying asleep. Previous work has shown that melatonin supplementation can be an effective treatment for inducing sleepiness and increasing sleep efficiency. This current study assessed the efficacy of a low dose melatonin spray on a population of people with sub-threshold insomnia who wake up at night and have difficulty falling back asleep.

**Methods:** This study consisted of 46 participants, n = 28 women (mean ± SD: age = 29.98 ± 12.72 years; height = 169.46 ± 10.44 cm; weight = 75.86 ± 19.50 kg,) with an Insomnia Severity Index (ISI) score between 8 – 14 (subthreshold insomnia). The study lasted 6 weeks and was a randomized, double blind, crossover design. Participants were given either a placebo or a melatonin spray (0.5 mg Melatonin/spray) and were instructed to use the spray orally (one or two sprays per use) for each awakening after falling asleep. Each participant first went through a 1-week screening, followed by 2 weeks of one treatment (melatonin or placebo), 1 washout week, then 2 weeks of the other treatment (melatonin or placebo). The following data were collected: demographic information, one night of Electroencephalogram (EEG) for sleep, actigraphy, sleep diary, Profile of Mood States (POMS) scores, Epworth Sleepiness Scale (ESS) scores, Pittsburgh Sleep Quality Index (PSQI), and the Restorative Sleep Questionnaire (RSQ) scores. Except for demographic and EEG data, which were collected during baseline week, all other measures were collected for all conditions.

**Results:** Statistical analysis showed no significant effect on melatonin sprays on all measures. However, during the screening overnight EEG, we observed a significant negative correlation between slow wave sleep (SWS) and age (r = −0.31, p < 0.05). Accordingly, we analyzed the data separately for those under 35 years old (n = 32) and those ≥35 years old (n = 14). The melatonin condition in the ≥ 35 years group showed significantly less time in bed (p = 0.017, Cohen’s d = 0.73) and less total sleep time (p = 0.021, Cohen’s d = 0.71). However, the Melatonin condition resulted in significantly less daytime sleepiness (ESS, p = 0.03, Cohen’s d = 0.64) and a trend for better sleep quality PSQI scores (p = 0.13, Cohen’s d = 0.27) compared to the placebo condition. No effect was observed for those in the <35 years group.

**Conclusions:** It is possible that melatonin spray increases SWS in those over 35 years old, which leads to a reduced sleep and time in bed and less daytime sleepiness. This may have implications regarding athletic performance- especially in older athletes. A follow-up study to explore this relationship is recommended.

**Body composition measurement differences between air displacement plethysmography and dual energy x-ray absorptiometry in men and women NCAA DI athletes**

Meghan K Magee^a,b^, Jennifer B Fields^b,c^, Andrew Jagim^b,d^, Margaret T Jones^b,e^

^a^School of Kinesiology, George Mason University, Manassas, VA, USA^b^Patriot Performance Laboratory, Frank Pettrone Center for Sports Performance, George Mason University, Fairfax, VA, USA^c^Exercise Science and Athletic Training, Springfield College, Springfield, MA, USA^d^Sports Medicine, Mayo Clinic Health System, La Crosse, WI, USA^e^Sport, Recreation, and Tourism Management, George Mason University, Fairfax, VA, USA

Corresponding author: mmagee2@gmu.edu

**Background:** Dual energy x-ray absorptiometry (DXA) is often regarded as the gold standard in body composition assessment; however, due to expense, training requirements, and associated radiation exposure, its use may not be feasible for athletic departments. Air displacement plethysmography (ADP) is a reliable, easy-to-use, and non-radiation method of body composition measurement, making it is more practical for general use. Previous literature indicates ADP may underestimate percent body fat (%BF) in lean individuals. Athletes tend to have more fat free mass (FFM) than non-athletes; therefore, ADP may underestimate %BF, which could lead to inappropriate characterization of body composition parameters. The purpose of this study was to examine the differences in %BF, FFM, and fat-free mass index (FFMI) when assessed via ADP and DXA in men and women NCAA DI collegiate athletes. A secondary purpose was to evaluate the DXA derived body volume equation compared to body volume obtained from ADP in an athletic population.

**Methods:** A total of 68 men and 123 women collegiate athletes participated. ADP and DXA measurements were taken within 48-hours of each other. For DXA testing, athletes were instructed to remove jewelry, wear clothing with no metal, and lie in the center of the DXA table with hands pronated at their side while feet turned inward with toes touching. For ADP testing, athletes were instructed to refrain from exercise, eating, and drinking for > 2 hours before testing, to remove jewelry, and to wear formfitting sports bra (women), spandex shorts, and a swim cap. Bare foot height and body mass were measured using a stadiometer and digital scale, respectively. %BF and FFM were recorded from each measurement. FFMI was calculated using the appropriate FFM value. Body volume was obtained from ADP, and also calculated using DXA values via a previously published equation. A one-way analysis of variance was used to identify differences (p ≤0.05). Effect sizes were evaluated through Cohen’s d.

**Results:** Table 1 includes descriptive information for all athletes. Differences between DXA and ADP were observed for %BF in men (mean difference = 4.5%; d = 0.92; p <0.001) and women (mean difference = 2.0%; d = 0.32; p =0.013). Further, differences in FFM were observed in women (mean difference = −5.2 kg; d = 0.43; p <0.001), as well as for FFMI in men (mean difference = −1.1 kg; d = 0.38; p =0.028) and women (mean difference = −1.8 kg; d = 0.52; p <0.001). No differences were identified between DXA and ADP for body volume.

**Table 1.** Athlete characteristics.

Sex	Men	Women
Age (years)	20.0 ±1.3	19.6 ±1.2
Height (cm)	185.2 ±10.7	172.4 ±8.0
Mass (kg)	87.3 ±16.6	73.0 ±14.8
%BF ADP	11.4 ±5.9 (0.7232)*	25.5 ±7.2 (0.6546)*
%BF DXA	15.9 ±3.6 (0.4407)	27.5 ±5.1 (0.4607)
FFM ADP (kg)	77.1 ±11.9 (1.3615)	53.8 ±9.6 (0.7615)*
FFM DXA (kg)	73.4 ±12.6 (1.4124)	48.6 ±13.9 (1.2664)
FFMI ADP (kg/m2)	22.4 ±2.7 (0.2759)*	18.0 ±2.7 (0.1969)*
FFMI DXA (kg/m2)	21.3 ±3.0 (0.3052)	16.2 ±4.1 (0.3693)
Body Volume ADP	80.5 ±16.5 (2.0330)	71.1 ±14.1 (1.3006)
Body Volume DXA	81.6 ±16.7 (2.0614)	70.8 ±14.3 (1.3182)

Values are represented as mean±standard deviations (SEE) *Indicates significant difference compared to DXA. %BF: percent body fat; ADP: air displacement plethysmography; DXA: dual energy x-ray absorptiometry; FFM: fat free mass; FFMI: fat free mass index.

**Conclusions:** Differences in body composition between measurement modalities existed in men and women athletes. ADP underestimated %BF in men and women athletes by ~4.5% and 2.0%, respectively. FFM from DXA was lower in women by 4-5 kg. While not different in men, it may be of clinical interest that FFM was ~4.0 kg lower for DXA (ES = 0.30). When calculated from DXA, FFMI was lower in men and women by 1.1 kg of FFM/m2 and 1.8 kg of FFM/m2, respectively. It is recommended that practitioners be aware of the underestimation of %BF from ADP and exercise caution when using the different modalities to track changes in body composition.

**Performance, soreness, and recovery changes in response to a high-volume dose of resistance exercise after supplementation of inactive and active *Bacillus coagulans* GBI-30, 6086**

Anthony Hagele^a^, Jessica M Moon^b^, Julia C. Blumkaitis^a^, Kayla M Ratliff^a^, Johnathan Boring^a^, Connor Gaige^a^, Logan Orr^a^, Kylie Walden^a^, Richard A Stecker^a^, Ralf Jäger FISSNc, Petey Mumford^a^, Kyle Sunderland^a^, Chad M Kerksick FISSN^a^

^a^Exercise and Performance Nutrition Laboratory, College of Science, Technology, and Health, Lindenwood University, St. Charles, MO, USA^b^School of Kinesiology, University of Central Florida, Orlando, FL, USA^c^Increnovo LLC, Milwaukee, WI, USA

Corresponding author: ckerksick@lindenwood.edu

**Background:** Exercise consisting of high-volume eccentric contractions has been known to damage muscle fibers resulting in a loss of contractile force, muscular power, and increases in soreness up to 72-hours post-exercise. Recently, probiotics have garnered interest for their ability to mitigate these changes. Supplementation with the strain *Bacillus coagulans* GBI-30, 6086 has demonstrated the potential to support improvements in performance and recovery, however, this research has been minimal. Further, inactivated probiotic strains have also amassed interest, but research into any performance modulation is lacking. This study’s purpose was to identify the impact of supplementation with inactive and active cultures of *Bacillus coagulans* GBI-30, 6086 on muscular performance, soreness, and recovery after a high-volume dose of resistance exercise.

**Methods:** 76 healthy, resistance-trained men (29.7 ±9.3 years, 178.6 ±7.5 cm, 87.4 ±14.3 kg, 20.7 ±4.5% fat) were randomly assigned in a double-blind, parallel-group fashion to supplement for 14-days with 1 billion CFU/day of either a heat-killed, inactivated culture of B. coagulans GBI-30, 6086 (INBC30, Kerry), an active culture of B. coagulans GBI-30, 6086 (BC30, Kerry), or a placebo (PLA). Participants completed 30-minutes of cycling intervals, followed by high-volume (5-6 sets of 10-12 RM) resistance exercises and 5 × 20 drop jumps. Prior to and 0-, 0.5-, 1-, 2-, 5-, 24-, 48-, and 72-hours after completing exercise, changes in muscular performance, soreness, and recovery were assessed.

**Results:** Changes in pressure-to-pain threshold (PPT) between PLA and INBC30 were different (p =0.01). Five-hours post-exercise, participants in the INBC30 group had a significantly lower PPT when compared to PLA (p =0.04). No changes in PPT between PLA and BC30 were identified (p =0.11). Further, changes in perceived recovery were identified at every timepoint (p <0.05) between PLA and BC30 with no changes identified between PLA and INBC30. All performance variables significantly decreased in response to the exercise bout (p <0.001) before returning to pre-exercise values, but no differences between groups were identified.

**Conclusions:** While performance was unaffected, BC30 supplementation, when compared to PLA, was able to improve perceptions of recovery in the hours and days following a high-volume resistance exercise bout. When compared to PLA, INBC30 reported a decreased ability to tolerate pain 5-hours after exercise.

**Acknowledgments:** This study was funded by an unrestricted grant from Kerry Foods. The authors declare no conflict of interest.

**Effects of a Thermogenic Supplement on Metabolic, Hemodynamics, and Subjective Mood State Outcomes in Caffeine-habituated Young Males**

Dylon Miller^a^, Jessica M Prather^a^, Christine M Florez^a^, Amie Vargas^a^, Bella Soto^a^, Abby Harrison^a^, Matthias Tinnin^a^, Grant M Tinsley^b^, Lem W Taylor^a^

^a^Human Performance Laboratory, School of Exercise and Sport Science, University of Mary Hardin-Baylor, Belton, TX, 76,513, USA^b^Energy Balance and Body Composition Laboratory, Department of Kinesiology & Sport Management, Texas Tech University, Lubbock, TX, 79,409, USA

Corresponding author: ltaylor@umhb.edu

**Background:** Thermogenic dietary supplements – which typically contain caffeine or other stimulants – are widely used by consumers, but safety and efficacy research is needed. The purpose of this study was to determine the effects of a specific thermogenic supplement on metabolic rate, heart rate, blood pressure and mood states.

**Methods:** Twenty-four males (179.3 ± 7.3 cm; 23.7 ±5.7y; 92.4 ±12.3 kg) who were moderate caffeine consumers (<150 mg/day) participated in this randomized, placebo-controlled crossover trial including two testing sessions separated by a 1-week washout. Participants were randomized into one of two groups (placebo [PL] versus 2 servings of OxyShred supplement, containing a total of 300 mg of caffeine and 3 g acetyl L-carnitine [TR]). Baseline measures for hemodynamics (SBP, DBP, HR), metabolic variables (REE, RQ, VCO2, VO2), indices of mood state, and hunger were assessed prior to the administration of thermogenic treatment (TR) or placebo (PL) with follow up assessments occurring at 30, 60, and 120 minutes post-ingestion. All variables were assessed with a repeated-measures analysis of variance (ANOVA) and follow up pairwise comparisons with Tukey adjustment. Statistical significance was accepted at p <0.05.

**Results:** Acute ingestion of TR resulted in a significant condition by time interaction for REE (p <0.0001) with follow up testing indicating higher REE in the TX group as compared to the PL group at magnitudes ranging from 11.5 to 13.6% at the 30-, 60-, 120- minute post-ingestion timepoints (p <0.05). No significant main effects or interactions were observed for HR, SBP, and DBP, except for a time main effect for HR (p <0.01), with follow up indicating a decrease in HR from baseline to 30 min regardless of condition. A significant interaction was found for VAS scale measures of energy (p <0.01), indicating that the ingestion of TR significantly increased perceived energy levels at 30 minutes post-ingestion. No significant interactions were observed for alertness, concentration, fatigue, focus or hunger/satiety. No differences in assessed side effects (dizziness, heart racing/skipping, shortness of breath, nervousness or blurred vision) were observed between groups.

**Conclusions:** Findings of this intervention suggest that consuming two servings of a commercially available thermogenic supplement results in a sustained increase in oxygen consumption and metabolic rate for at least a 2-hour period post consumption. The high dose of caffeine and other stimulant ingredients did not result in any adverse events or negative alterations in hemodynamic function.

**Acknowledgments:** This study was funded by EHP Labs. The authors declare no conflicts of interest.

**Efficacy of a Microalgae Extract Combined with Natural Guarana on Cognitive Performance of Gamers IV: Psychomotor Vigilance**

Jacob Kendra^a^, Megan Leonard^a^, Jonathan Maury^b^, Drew Gonzalez^a^, Broderick Dickerson^a^, Tori Jenkins^a^, Kay Nottingham^a^, Choongsung Yoo^a^, Dante Xing^a^, Joungbo Ko^a^, Rémi Pradelles^b^, Ryan Sowinski^a^, Christopher J. Rasmussen^a^, Richard B. Kreider, FISSN^a^

^a^Exercise & Sport Nutrition Laboratory, Human Clinical Research Facility, Texas A&M University, College Station, TX 77843, USA^b^Microphyt, Research & Development Department, Baillargues, Mudaison, FR

Corresponding author: rbkreider@tamu.edu

**Background:** Competitive gaming requires visual selective attention, short-term memory or task switching, and an ability to sustain a high level of energy over time. Fucoxanthin is a major carotenoid, found in specific microalgae varieties like Phaeodactylum tricornutum that has been reported to possess neuroprotective and nootropic effects through its anti-inflammatory and antioxidant activities on different signaling pathways like Nrf2-ARE. The purpose of this study was to evaluate whether acute and 30-day supplementation of a microalgae extract from Phaeodactylum tricornutum with Guarana would affect cognitive function of gamers.

**Methods:** In a double-blind, placebo-controlled manner, 51 male and 10 female experienced gamers (21.7 ±4 years, 73.0 ±13 kg, 24.2 ±3.6 kg/m2) were randomly assigned to ingest a placebo (PL); low-dose (LD) of GamePhyt™ (MicroPhyt, Baillargues, FR) containing 440 mg/day of Phaeodactylum tricornutum extract including 1% Fucoxanthin + 440 mg/day of guarana, or high-dose (HD) of GamePhyt™ containing 2 × 440 mg/day of Phaeodactylum tricornutum extract including 1% Fucoxanthin + 440 mg/day of guarana for 30-days. Participants refrained from consuming atypical amounts of stimulants, food, and supplements that may affect cognition during the study. Acute (single dose) cognitive function tests were administered on Day 0 prior to supplementation, 15-min post-supplementation, and after the participants played their most competitive video game for 60-minutes. Participants continued supplementation for 30-days and then repeated pre-supplementation and post-gaming cognitive function tests. The battery of cognitive function tests included the Psychomotor Vigilance Task Test (PVTT) which assesses sustained attention reaction times through responses to visual stimuli (as light) requiring participants to press a keyboard button in response to a randomly illuminating light on screen every few seconds. The number of times the button was not pressed, and the speed of response was measured, with sleepiness quantified as the number of lapses in attention during the test. A total of 20 trials were performed. Data were analyzed by General Linear Model (GLM) univariate analyses with repeated measures using weight as a covariate and mean and percent changes from baseline with 95% confidence intervals.

**Results:** Figure 1 presents selected analyses performed on PVTT data. Results revealed evidence that acute LD ingestion significantly reduced pre-game (Pre-G) Trial 6 reaction times. After 30-days of supplementation, reaction times in Trial 6 were faster prior to and following gaming with LD ingestion. However, no significant differences were observed in 20 Trial mean reaction time responses.

**Figure 1.**
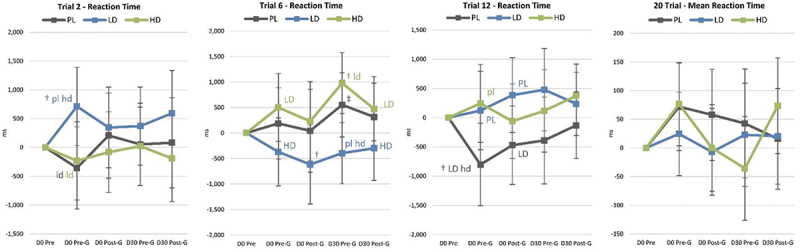
Changes in Phychomoto Vigilance Task Tests result. Data are means and 95% confidence intervals. Changes from baselind are show as † (p<0.05 change from baseline) and ‡ (p<0.05 to p<0.10 trends from baseline). Small case letters indicate p<0.05 differences from placebo (pl), low dose (ld), or high dose (hl) whileupper-case letters (PL, LD, HD) indicate trends (p<0.0-5 to p<01.10).

**Conclusions:** Results provide some evidence that acute and chronic supplementation with a microalgae extract from Phaeodactylum tricornutum with Guarana can affect sustained attention reaction times and lapses in attention.

**Acknowledgments:** This study was funded by MicroPhyt (Baillargues, FR) as a fee-for-service project to the Human Clinical Research Facility at Texas A&M University and conducted by the Exercise & Sport Nutrition Lab.

